# Methylprednisolone as Adjunct to Thrombectomy for Acute Intracranial Internal Carotid Artery Occlusion Stroke: Post Hoc Secondary Analysis of the MARVEL Randomized Clinical Trial

**DOI:** 10.1001/jamanetworkopen.2024.59945

**Published:** 2025-02-18

**Authors:** Chong Zheng, Rongtong Li, Chaoxiong Shen, Fang Guo, Daofeng Fan, Lixian Yang, Li Zhang, Anni Chen, Yangui Chen, Dongping Chen, Wenjie Zi, Changwei Guo, Thanh N. Nguyen, Gregory W. Albers, Bruce C. V. Campbell, Zhongming Qiu, Zhizhou Hu

**Affiliations:** 1 Department of Neurology, Longyan First Affiliated Hospital of Fujian Medical University, Longyan, China; 2 Department of Neurology, Xinqiao Hospital and The Second Affiliated Hospital, Army Medical University (Third Military Medical University), Chongqing, China; 3 Department of Neurology, Boston Medical Center, Boston, Massachusetts; 4 Department of Radiology, Boston Medical Center, Boston, Massachusetts; 5 Department of Neurology and Neurological Sciences, Stanford University School of Medicine, Palo Alto, California; 6 Department of Medicine and Neurology, Melbourne Brain Centre at the Royal Melbourne Hospital, University of Melbourne, Parkville, Victoria, Australia; 7 Department of Neurology, The 903rd Hospital of The Chinese People’s Liberation Army, Hangzhou, China

## Abstract

**Importance:**

Patients with acute ischemic stroke (AIS) due to intracranial internal carotid artery (ICA) occlusion often have poor functional outcomes despite undergoing endovascular thrombectomy (EVT).

**Objective:**

To investigate the effectiveness and safety associated with intravenous methylprednisolone as adjunctive treatment to EVT for patients with AIS due to intracranial ICA occlusion.

**Design, Setting, and Participants:**

This was a post hoc analysis of the MARVEL randomized, double-blind, placebo-controlled clinical trial conducted from February 9, 2022, to June 30, 2023, at 82 stroke centers across China with a 3-month follow-up. The primary trial enrolled 1680 patients with large vessel occlusion within 24 hours from last known well time in the intracranial ICA, the first segment of the middle cerebral artery (M1), or the second segment of the middle cerebral artery (M2), of whom 579 patients had intracranial ICA occlusion.

**Intervention:**

Intravenous methylprednisolone, 2 mg/kg/d (maximum dose, 160 mg) for 3 days plus EVT vs placebo plus EVT.

**Main Outcomes and Measures:**

The primary outcome was independent ambulation at 90 days, defined as a score of 0 to 3 on the modified Rankin Scale (range, 0 [no symptoms] to 6 [death]). Safety outcomes included death within 90 days, symptomatic intracranial hemorrhage (sICH) within 48 hours, and decompressive hemicraniectomy to relieve midline-shift and intracranial pressure after EVT.

**Results:**

Among 579 patients (median age, 69.0 years [IQR, 59.0-76.0 years]; 338 men [58.4%]), there were 286 patients in the methylprednisolone group and 293 patients in the placebo group. The proportion of patients who achieved 90-day independent ambulation was significantly higher in the methylprednisolone group than in the placebo group (151 of 284 [53.2%] vs 125 of 293 [42.7%]; adjusted risk ratio [RR], 1.27 [95% CI, 1.07-1.52]; *P* = .007). The incidence of sICH was lower in the methylprednisolone group than in the placebo group (26 of 277 [9.4%] vs 45 of 290 [15.5%]; adjusted RR, 0.55 [95% CI, 0.35-0.87]; *P* = .01). The rate of decompressive hemicraniectomy was lower in the methylprednisolone group compared with the placebo group (16 of 286 [5.6%] vs 29 of 293 [9.9%]; adjusted RR, 0.54 [95% CI, 0.30-0.98]; *P* = .04). No significant difference was observed in mortality between groups (methylprednisolone, 92 of 284 [32.4%] vs placebo, 111 of 239 [37.9%]; adjusted RR, 0.84 [95% CI, 0.67-1.05]; *P* = .13).

**Conclusions and Relevance:**

In this secondary analysis of a randomized clinical trial of intravenous methylprednisolone vs placebo for patients with intracranial ICA occlusion undergoing EVT, intravenous methylprednisolone was associated with improved ambulation. These findings suggest that the use of intravenous methylprednisolone as an adjunct to EVT may hold promise as a treatment option for patients with AIS due to intracranial ICA occlusion.

**Trial registration:**

ChiCTR.org.cn Identifier: ChiCTR2100051729

## Key Points

**Question:**

Is intravenous methylprednisolone as an adjunct to endovascular thrombectomy (EVT) associated with improved clinical outcomes among patients with acute ischemic stroke (AIS) due to intracranial internal carotid artery (ICA) occlusion?

**Findings:**

In this post hoc secondary analysis of a randomized clinical trial of 579 patients with AIS due to intracranial ICA occlusion who underwent EVT, intravenous methylprednisolone was associated with a higher rate of 90-day independent ambulation compared with placebo (53.2% vs 42.7%, respectively).

**Meaning:**

This study suggests that intravenous methylprednisolone as an adjunct to EVT may be a promising treatment option for patients with AIS due to ICA occlusion.

## Teaser

This post hoc secondary analysis of the MARVEL randomized clinical trial investigates the effectiveness and safety associated with intravenous methylprednisolone as adjunctive treatment to endovascular thrombectomy for patients with acute ischemic stroke due to intracranial internal carotid artery occlusion.

## Introduction

Acute ischemic stroke (AIS) is a leading cause of mortality and long-term disability worldwide.^1^ Occlusion of the intracranial internal carotid artery (ICA) is one of the most catastrophic subtypes, which accounts for 20% of patients with mild-to-moderate ischemic core stroke and nearly 40% of patients with large ischemic core stroke.^2,3,4,5,6^ Endovascular thrombectomy (EVT) has been proven to be a safe and effective treatment for patients with AIS attributable to large vessel occlusion. Although studies have shown that patients with intracranial ICA occlusion can benefit from EVT, less than 30% of patients with intracranial ICA occlusion achieve functional independence after EVT.^7,8^ In the EVT era, the priority has been shifted from whether patients should be treated to how to improve outcomes after EVT.^9,10^

Acute ischemic stroke leads to the breakdown of the blood-brain barrier (BBB), promotes angiogenic edema, and triggers the release of various inflammatory cytokines and oxygen free radicals. This inflammatory response can worsen early neurologic deficits.^11,12^ Previous studies have shown that glucocorticoids can regulate inflammation, reduce pro-inflammatory cytokine levels, alleviate the inflammatory response, and decrease apoptosis of neuronal cells in central nervous system injury.^13,14^ Methylprednisolone is a glucocorticoid and has more lipid antioxidant activity than other steroids used as an immunosuppressive agent in clinical practice to treat several conditions, including myasthenia gravis, autoimmune encephalitis, and multiple sclerosis, among many others.^15,16,17^ The Methylprednisolone as Adjunctive to Endovascular Treatment for Acute Large Vessel Occlusion (MARVEL) trial showed that methylprednisolone added to EVT did not improve the degree of overall disability.^18^ These findings suggest that a potential explanation for the primary study results is that this study included a broad range of patients with large vessel occlusion with various occlusion sites. Considering that patients with an intracranial ICA occlusion typically have a substantial thrombus burden, poor collateral circulation, large ischemic core, and catastrophic clinical prognosis, it remains unclear as to whether methylprednisolone may show differential outcomes among patients with stroke attributable to occlusion of the intracranial ICA. In this context, we performed a post hoc secondary analysis of the MARVEL trial to evaluate the association between methylprednisolone and outcomes after EVT among patients with stroke attributable to occlusion of the intracranial ICA.

## Methods

### Study Design

This was a post hoc secondary analysis of the MARVEL trial, an investigator-initiated, randomized, double-blind, placebo-controlled clinical trial conducted at 82 stroke centers across China from February 9, 2022, to June 30, 2023, with follow-up for 3 months. The trial enrolled patients with large vessel occlusion within 24 hours from last known well time located in the intracranial ICA, the first segment of the middle cerebral artery (M1), or the second segment of the middle cerebral artery (M2). The objective of the MARVEL trial was to evaluate the effectiveness and safety of adjunctive intravenous methylprednisolone vs placebo among patients with AIS secondary to large vessel occlusion undergoing EVT. The ethics committees of the Xinqiao Hospital, Army Medical University, and all participating hospitals approved this study. Written informed consent was provided by all patients or their legally authorized representative before randomization. Detailed information regarding the selection criteria, trial protocol, end point adjudication, and primary results were previously published^19^ (trial protocol and statistical analysis plan in Supplement 1). The MARVEL trial followed the Consolidated Standards of Reporting Trials (CONSORT) reporting guideline.

### Participants and Interventions

For this study, we included patients from the MARVEL trial who had intracranial ICA occlusion identified using computed tomographic angiography, magnetic resonance angiography, or digital subtraction angiography. Eligible patients in the trial were 18 years or older with a National Institutes of Health Stroke Scale (NIHSS) score of 6 or more (range, 0-42, where higher scores indicate more severe neurologic deficit) at the time of randomization and a modified Rankin Scale (mRS) score of less than 2 (range, 0-6, where 0 indicates no symptoms and 6 indicates death) before the stroke. Eligible patients were randomized to receive intravenous methylprednisolone at a dose of 2 mg/kg/d (up to a maximum dose of 160 mg) for 3 days plus EVT vs placebo plus EVT.

### Outcomes

The primary effectiveness outcome of this analysis was independent ambulation as indicated by a mRS score of 0 to 3 at 90 days. Secondary effectiveness outcomes included the NIHSS score at 5 to 7 days or at early discharge; the mRS score distribution at 90 days, the following prespecified dichotomizations of the mRS score at 90 days: 0 to 2 vs 3 to 6, and 0 or 1 (or return to prestroke mRS score) vs 2 to 6; and score on the European Quality of Life 5-Dimension visual analog scale (EQ-5D-VAS; range, 0-100, with lower scores denoting a worse quality of life) at 90 days.

Safety outcomes included death from any cause within 90 days, symptomatic intracranial hemorrhage (sICH) within 48 hours, any radiologic intracranial hemorrhage within 48 hours, and decompressive hemicraniectomy. Intracranial hemorrhage was assessed according to the modified Heidelberg bleeding classification (an increase in the NIHSS score of 4 points or an increase in the score for an NIHSS subcategory of 2 points with any intracranial hemorrhage on imaging results).^20^ Patients underwent decompressive hemicraniectomy to relieve midline-shift and intracranial pressure. Other safety variables, including the incidence of gastrointestinal bleeding within 7 days after EVT and pneumonia, were recorded.

### Statistical Analysis

Analysis of the primary outcome was based on the full analysis set population, which included patients according to their randomization assignment, with a valid mRS score assessment at 90 days. The primary outcome was adjusted using the inverse probability treatment weighting (IPTW) method (eMethods in Supplement 2). The IPTW was calculated for patients with intracranial carotid occlusion adjusted for 7 covariates: age, baseline NIHSS score, prestroke mRS score, baseline Alberta Stroke Program Early CT [Computed Tomography] Score (ASPECTS), use of intravenous thrombolysis, time from stroke onset to randomization, and cause of stroke. Inverse probability treatment weighting could reduce the imbalance in confounders between treatment groups, which has been shown as an effective approach in multiple clinical retrospective studies.^21^ The area under the curve of the logistic regression model to estimate the propensity score was 0.60, indicating that propensity score matching provided adequate discrimination between the groups. For binary outcomes, risk ratios (RRs) were calculated using modified Poisson regression.^22,23^ In addition, odds ratios calculated by the generalized linear model with binomial distribution and logit link function and risk differences calculated by the generalized linear model with binomial distribution and identity link function were also provided,^24^ and the adjusted value was estimated by the IPTW methods. The NIHSS scores and EQ-5D-VAS scores were analyzed by linear regression adjusted using the IPTW method. For distribution of mRS score at 90 days, the covariate-adjusted proportional odds regression model was used to calculate the common odds ratio (cOR) for a shift in the direction of a better outcome on the mRS.^25^ Continuous variables are expressed as median and IQR values; categorical data are presented as frequencies and proportions. The estimates of RRs, odds ratios, risk differences, cORs, β coefficients, and 95% CIs were reported. All *P* values were 2-tailed, and statistical significance was set at *P* < .05. Statistical analyses were performed using SAS, version 9.4 (SAS Institute Inc).

## Results

### Patient Characteristics 

**Figure 1.**
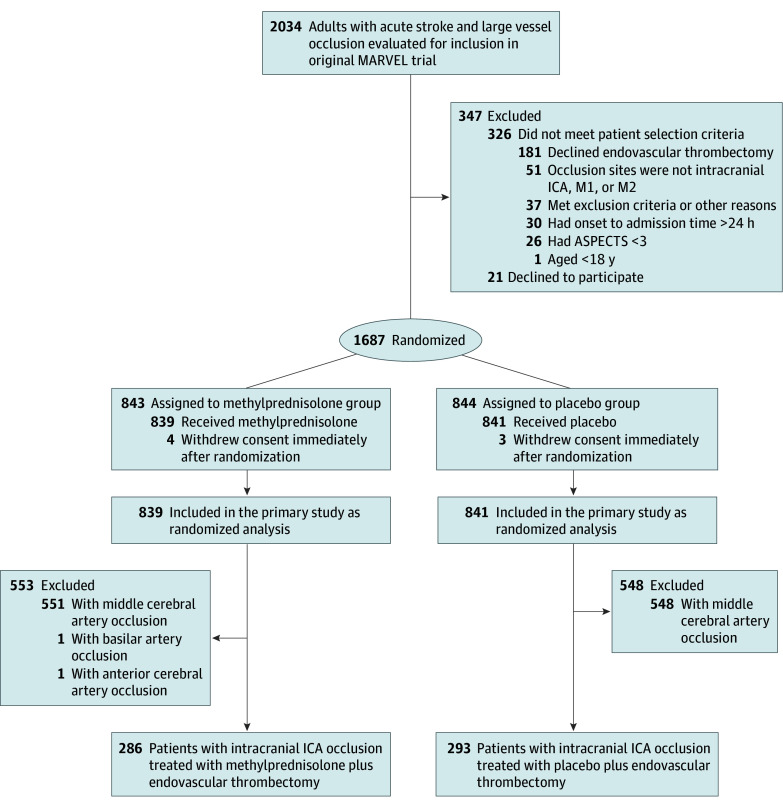
Flowchart of Patient Selection ASPECTS indicates Alberta Stroke Program Early CT [Computed Tomography] Score; ICA, internal carotid artery; MARVEL, Methylprednisolone as Adjunctive to Endovascular Treatment for Acute Large Vessel Occlusion; M1, the first segment of middle cerebral artery; and M2, the second segment of the middle cerebral artery.

There were 579 patients (median age, 69.0 years [IQR, 59.0-76.0 years]; 338 men [58.4%] and 241 women [41.6%]) with intracranial ICA occlusion included in this analysis (Figure 1 and Table 1). The median baseline NIHSS score was 19.0 (IQR, 17.0-21.0). The median baseline ASPECTS was 5.0 (IQR, 4.0-7.0). There were 286 patients in the methylprednisolone group and 293 patients in the placebo group. Compared with the placebo group, patients in the methylprednisolone group had a shorter median onset to recanalization time (421.0 minutes [IQR, 310.0-702.0 minutes] vs 480.0 minutes [IQR, 345.0-720.0 minutes]) and different stroke causes (large artery atherosclerosis, 84 of 286 patients [29.4%] vs 117 of 293 patients [39.9%]; cardioembolism, 156 of 286 patients [54.5%] vs 125 of 293 patients [42.7%]; and other and unknown causes, 46 of 286 patients [16.1%] vs 51 of 293 patients [17.4%]). Moreover, patients in the methylprednisolone group had different prestroke mRS score (score 0: 269 of 286 [94.1%] vs 284 of 293 [96.9%]; score 1: 14 of 286 [4.9%] vs 4 of 293 [1.4%]; score 2: 3 of 286 [1.0%] vs 5 of 293 [1.7%]). Other baseline variables were balanced between the methylprednisolone and placebo groups. Baseline demographic characteristics of participants included in these analyses are listed in Table 1.

**Table 1.** Baseline Demographic and Clinical Characteristics of Patients With Intracranial Internal Carotid Artery Occlusion

Characteristic	Total (N = 579)	Methylprednisolone (n = 286)	Placebo (n =293)	Standardized difference	*P* value
Age, median (IQR), y	69.0 (59.0 to 76.0)	69.0 (59.0 to 76.0)	69.0 (59.0 to 77.0)	−0.043	.62
Sex, No. (%)					
Female	241 (41.6)	128 (44.8)	113 (38.6)	0.126	.13
Male	338 (58.4)	158 (55.2)	180 (61.4)		
Medical history, No. (%)^a^					
Hypertension	342 (59.1)	164 (57.3)	178 (60.8)	−0.069	.40
Atrial fibrillation	248 (42.8)	129 (45.1)	119 (40.6)	0.091	.28
Hyperlipidemia	161 (27.8)	84 (29.4)	77 (26.3)	0.069	.41
Diabetes	105 (18.1)	53 (18.5)	52 (17.7)	0.020	.81
Coronary heart disease	115 (19.9)	58 (20.3)	57 (19.5)	0.021	.80
Cerebral ischemia	83 (14.3)	46 (16.1)	37 (12.6)	0.099	.24
Intracranial hemorrhage	9 (1.6)	6 (2.1)	3 (1.0)	0.087	.30
Transient ischemic attack	2 (0.3)	2 (0.7)	0	0.119	.15
Smoking (current)	171 (29.5)	78 (27.3)	93 (31.7)	−0.098	.24
Prestroke modified Rankin Scale score, No. (%)^b^					
0	553 (95.5)	269 (94.1)	284 (96.9)	0.077	.04
1	18 (3.1)	14 (4.9)	4 (1.4)		
2	8 (1.4)	3 (1.0)	5 (1.7)		
Glucose at hospital arrival, median (IQR), mg/dL^c^	129.7 (111.7 to 156.8)	131.5 (113.5 to 158.6) [n = 254]	126.1 (109.9 to 153.2) [n = 254]	0.128	.08
Baseline NIHSS score, median (IQR)^d^	19.0 (17.0 to 21.0)	19.0 (17.0 to 21.0)	19.0 (17.0 to 21.0)	0.003	.91
Baseline ASPECTS, median (IQR)^e^	5.0 (4.0 to 7.0)	5.0 (4.0 to 7.0)	6.0 (4.0 to 7.0)	−0.078	.35
TOAST cause, No. (%)^f^					.01
Cardioembolism	281 (48.5)	156 (54.5)	125 (42.7)	0.223	
Large artery atherosclerosis	201 (34.7)	84 (29.4)	117 (39.9)	−0.240	
Other and unknown	97 (16.8)	46 (16.1)	51 (17.4)	−0.035	
Intravenous thrombolysis, No. (%)	209 (36.1)	96 (33.6)	113 (38.6)	−0.104	.21
Time from last known well time, median (IQR), min					
To randomization	353.0 (239.0 to 598.0)	328.0 (224.0 to 597.0)	374.0 (264.0 to 599.0)	−0.053	.07
To recanalization	451.0 (328.0 to 715.0)	421.0 (310.0 to 702.0)	480.0 (345.0 to 720.0)	−0.056	.046
Time from randomization to initial treatment, median (IQR), h	8.0 (6.0 to 14.0)	8.0 (6.0 to 14.0)	8.0 (6.0 to 12.0)	0.121	.17
Blood pressure at hospital arrival, median (IQR), mm Hg					
Systolic	143.0 (124.0 to 161.0)	141.0 (123.0 to 162.0)	144.0 (124.0 to 161.0)	−0.041	.52
Diastolic	83.0 (74.0 to 94.0)	81.0 (74.0 to 92.0)	83.0 (73.0 to 94.0)	−0.068	.43

Abbreviations: ASPECTS, Alberta Stroke Program Early CT [Computed Tomography] Score; NIHSS, National Institutes of Health Stroke Scale; TOAST, Trial of ORG 10172 in Acute Stroke Treatment.

SI conversion factor: To convert glucose to millimoles per liter, multiply by 0.0555; and HbA_1c_ to proportion of total hemoglobin, multiply by 0.01.

^a^ Comorbidities based on family or patient report.

^b^ Scores on the modified Rankin Scale of functional disability range from 0 (no symptoms) to 6 (death).

^c^ Blood glucose values were missing in some patients: 32 cases were missing in the methylprednisolone group, and 39 cases were missing in the placebo group.

^d^ Scores on the NIHSS range from 0 to 42, with higher scores indicating more severe neurologic deficit.

^e^ The ASPECTS is an imaging measure of the extent of ischemic stroke. Scores range from 0 to 10, with higher scores indicating a smaller infarct core. Listed are values for the core laboratory assessment. ASPECTS data were missing for 3 patients in the methylprednisolone group and 2 patients in the placebo group.

^f^ The TOAST classification system is a widely used method for classifying ischemic stroke and transient ischemic attack. It divides ischemic stroke and transient ischemic attack into 5 subtypes based on their likely causes: large artery atherosclerosis, cardioembolism, small artery occlusion, other determined cause, and undetermined cause.

### Effectiveness Outcomes

**Figure 2.**
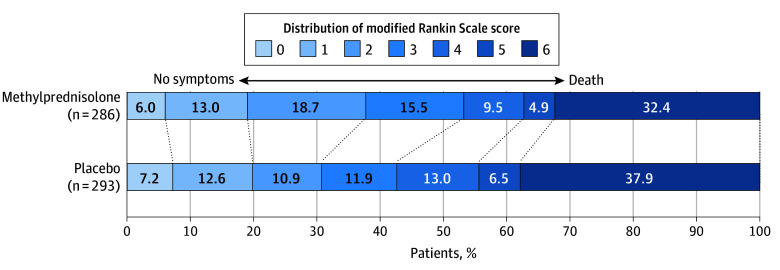
Distribution of Global Disability at 90 Days on the Modified Rankin Scale

A statistically significant heterogeneity of the treatment effect of methylprednisolone vs placebo was not found among patients with ICA occlusion (eTable 1 in Supplement 2). The proportion of patients with independent ambulation at 90 days was significantly higher among the methylprednisolone group than the placebo group (151 of 284 patients [53.2%] vs 125 of 293 patients [42.7%]; unadjusted RR, 1.25 [95% CI, 1.05-1.48]; *P* = .01 and adjusted RR, 1.27 [95% CI, 1.07-1.52]; *P* = .007) (Table 2; eTable 2 in Supplement 2; Figure 2). The median mRS score at 90 days was 3.0 (IQR, 2.0-6.0) in the methylprednisolone group and 4.0 (IQR, 2.0-6.0) in the placebo group. There was no significant difference in the distribution of 90-day mRS score between the methylprednisolone group and the placebo group, yielding an adjusted cOR of 1.14 (95% CI, 0.98-1.32; *P* = .09). The median EQ-5D-VAS score at 90 days was higher in the methylprednisolone group compared with the placebo group (42.5 [IQR, 0.0-75.0] vs 30.0 [IQR, 0.0-70.0]; adjusted β coefficient, 7.18 [95% CI, 1.18-13.18]; *P* = .02). There was no significant difference in the proportion of patients with an mRS score of 0 to 1 at 90 days and an NIHSS score at 5 to 7 days or earlier if discharged between the methylprednisolone and placebo groups.

**Table 2.** Effectiveness and Safety Outcomes of Intravenous Methylprednisolone vs Placebo Before Endovascular Thrombectomy Among Patients With Intracranial Internal Carotid Artery Occlusion

Outcome	Methylprednisolone (n = 286)	Placebo (n = 293)	Unadjusted value (95% CI)	*P* value	Adjusted value (95% CI)^a^	*P* value
Primary outcome						
mRS Score of 0-3 at 90 d, No./total No. (%)^b^	151/284 (53.2)	125/293 (42.7)	RR, 1.25 (1.05 to 1.48 )	.01	RR, 1.27 (1.07 to 1.52)	.007
Secondary outcomes^c^						
mRS Score at 90 d, median (IQR)	3.0 (2.0 to 6.0)	4.0 (2.0 to 6.0)	cOR, 1.27 (0.95 to 1.70)	.10	cOR, 1.14 (0.98 to 1.32)^d^	.09
mRS Score of 0-2 at 90 d, No./total No. (%)	107/284 (37.7)	90/293 (30.7)	RR, 1.23 (0.98 to 1.54 )	.08	RR, 1.24 ( 0.98 to 1.56)	.07
mRS Score of 0-1 at 90 d, No./total No. (%)	56/284 (19.7)	58/293 (19.8)	RR, 1.00 (0.72 to 1.38)	.98	RR, 1.02 ( 0.73 to 1.42)	.92
NIHSS score at 5-7 d or earlier if discharged, median (IQR)^e^	13.0 (5.0 to 32.0)	15.0 (6.0 to 35.0)	β, −1.75 (−4.19 to 0.68)	.13	β, −2.09 (−4.51 to 0.33)	.09
EQ-5D-VAS score at 90 d, median (IQR)^f^	42.5 (0.0 to 75.0)	30.0 (0.0 to 70.0)	β, 6.67 (0.65 to 12.68)	.03	β, 7.18 (1.18 to 13.18)	.02
Safety outcomes, No./total No. (%)						
Mortality	92/284 (32.4)	111/293 (37.9)	RR, 0.86 (0.68 to 1.07)	.17	RR, 0.84 (0.67 to 1.05)	.13
Symptomatic intracranial hemorrhage^g^	26/277 (9.4)	45/290 (15.5)	RR, 0.60 (0.38 to 0.95)	.03	RR, 0.55 (0.35 to 0.87)	.01
Any radiologic intracranial hemorrhage	113/277 (40.8)	122/290 (42.1)	RR, 0.97 (0.80 to 1.18)	.76	RR, 0.94 (0.77 to 1.15)	.55
Decompressive hemicraniectomy	16/286 (5.6)	29/293 (9.9)	RR, 0.57 (0.31 to 1.02)	.06	RR, 0.54 (0.30 to 0.98)	.04
Pneumonia	139/286 (48.6)	177/293 (60.4)	RR, 0.80 (0.69 to 0.94)	.005	RR, 0.80 (0.69 to 0.93)	.004
Gastrointestinal bleeding within 7 d after EVT, No./total No. (%)	15/286 (5.2)	20/293 (6.8)	RR, 0.77 (0.40 to 1.47)	.43	RR, 0.73 (0.38 to 1.42)	.36

Abbreviations: cOR, common odds ratio; EQ-5D-VAS, European Quality of Life 5-Dimension visual analog score; EVT, endovascular thrombectomy; mRS, modified Rankin Scale; NIHSS, National Institutes of Health Stroke Scale; RR, risk ratio.

^a^ Adjusted values were adjusted for age, baseline NIHSS score, prestroke mRS score, baseline Alberta Stroke Program Early CT [Computed Tomography] Score (ASPECTS), use of intravenous thrombolysis, time from onset to randomization, and cause of stroke, using the inverse probability of treatment weighting method. The RR and β coefficient was adjusted by the inverse probability treatment weighting method.

^b^ The mRS of functional disability ranges from 0 (no symptoms) to 6 (death). Data were missing for 2 patients in the methylprednisolone group.

^c^ The widths of the 95% CIs for the secondary outcomes were not adjusted for multiple comparisons.

^d^ The cOR indicated that the probability of the mRS score in the methyprednisolone group was lower than in the placebo group. Treatment estimation was adjusted for age, baseline NIHSS score, prestroke mRS, baseline Alberta Stroke Program Early CT [Computed Tomography] Score (ASPECTS), use of intravenous thrombolysis, time from onset to randomization, and cause of stroke.

^e^ Scores on the NIHSS range from 0 to 42, with higher values reflecting more severe neurologic impairment.

^f^ EQ-5D-VAS is a continuous scale measure of self-reported quality of life. Scores range from 0 to 100, with 0 indicating the worst possible quality of life and 100 to the best possible quality of life.

^g^ Symptomatic intracranial hemorrhage was defined according to the Heidelberg bleeding classification (an increase in the NIHSS score of 4 points or an increase in the score for an NIHSS subcategory of 2 points with any intracranial hemorrhage on imaging results).

### Safety Outcomes

No significant difference was observed in mortality between the methylprednisolone group and the placebo group (92 of 284 [32.4%] vs 111 of 239 [37.9%]), yielding an unadjusted RR of 0.86 (95% CI, 0.68-1.07; *P* = .17) and an adjusted RR of 0.84 (95% CI, 0.67-1.05; *P* = .13) (Table 2). The proportion of patients with sICH was significantly lower in the methylprednisolone group than the placebo group (26 of 277 [9.4%] vs 45 of 290 [15.5%]; adjusted RR, 0.55 [95% CI, 0.35-0.87]; *P* = .01). However, no significant difference was observed in the methylprednisolone group vs the placebo group in any radiologic intracranial hemorrhage (113 of 277 [40.8%] vs 122 of 290 [42.1%]; adjusted RR, 0.94 [95% CI, 0.77-1.15]; *P* = .55). Patients in the methylprednisolone group had a significantly lower rate of decompressive hemicraniectomy compared with those in the placebo group (16 of 286 [5.6%] vs 29 of 293 [9.9%]; adjusted RR, 0.54 [95% CI, 0.30-0.98]; *P* = .04). Pneumonia occurred in 139 of 286 patients (48.6%) in the methylprednisolone group and 177 of 293 patients (60.4%) in the placebo group (adjusted RR, 0.80 [95% CI, 0.69-0.93]; *P* = .004). There were no significant differences in gastrointestinal bleeding within 7 days after EVT between the methylprednisolone and placebo groups (15 of 286 [5.2%] vs 20 of 293 [6.8%]; adjusted RR, 0.73 [95% CI, 0.38-1.42]; *P* = .36).

## Discussion

In this post hoc secondary analysis of the MARVEL trial, we assessed the association between methylprednisolone as adjunct to EVT and independent ambulation at 90 days among patients with intracranial ICA occlusion. Our study revealed that methylprednisolone treatment was associated with a higher rate of independent ambulation. In addition, methylprednisolone treatment was significantly associated with a lower rate of sICH within 48 hours and decompressive hemicraniectomy compared with placebo. However, no significant difference was detected in the incidence of mortality at 90 days between the groups.

The intracranial ICA is a major blood vessel that supplies blood to the anterior circulation and is the most proximal artery of the anterior circulation. The diameter of the intracranial ICA vessel is larger than that of the middle cerebral artery, and intracranial ICA occlusions usually have a greater burden of thrombus. As an occlusion of the intracranial ICA can block flow to both the middle cerebral and anterior cerebral artery, these patients may present with a higher severity of neurologic deficit with higher a NIHSS score and larger-sized infarct core volume.^26^ Moreover, extension of thrombus into the ipsilateral M1 segment or first segment of the anterior cerebral artery (A1) can impair compensatory supply from leptomeningeal collaterals.^27^ Thrombus extension increases the difficulty of EVT and the number of retrieval attempts, while an increased number of recanalization attempts is associated with a worse outcome.^28,29^ Therefore, cerebral infarctions with intracranial ICA occlusion usually manifest with greater severity of neurologic deficit and poorer outcome.^30^

Due to the risk of infectious complications, the use of conventional or large doses of methylprednisolone is currently recommended to be avoided for patients with ischemic stroke.^31^ However, a clinical study indicated that patients with AIS improved their level of consciousness after glucocorticoid administration.^32^ Moreover, another study of patients with AIS found that glucocorticoid resistance was associated with poor functional outcomes after stroke.^33^ Animal studies showed that glucocorticoids can significantly reduce the levels of proinflammatory cytokines, thereby attenuating a chronic inflammatory response.^14^ Our subgroup analysis of the MARVEL trial found that, among patients with ICA occlusion undergoing EVT, methylprednisolone was associated with favorable clinical outcomes and improved independent ambulation (mRS score of 0-3) at 90 days, which indicates that the patient may be able to achieve independent living.^34^ This finding suggests that methylprednisolone treatment could be associated with an increased proportion of patients with an outcome of ambulation without depending on another person. The results may be explained by the presence of larger ischemic areas and greater severity of BBB breakdown for patients with intracranial ICA occlusion. The view would be compatible with the notion that corticosteroids may be beneficial for high-risk patients with large infarcts and vasogenic edema.

Symptomatic intracranial hemorrhage is a common but catastrophic complication after EVT in patients with intracranial ICA occlusion, with an incidence of approximately 8.5% in Spain, and is independently associated with neurologic deterioration and early death.^35,36^ Symptomatic intracranial hemorrhage after EVT is considered to be associated with acute reperfusion injury of the BBB.^37^ Our results suggest that methylprednisolone treatment was significantly associated with a lower rate of sICH within 48 hours among patients with intracranial ICA occlusion. This lower rate of sICH could be due to the action of glucocorticoids in minimizing BBB damage. Animal model research shows that corticosteroids enhance the integrity of the BBB and curtail its permeability by preserving the tight junctions between endothelial cells.^38,39^ Cerebral edema secondary to large hemispheric infarcts is well established to be associated with poor outcome or death, particularly malignant cerebral edema.^40^ Decompressive hemicraniectomy in patients with malignant cerebral edema increases the chance of a favorable outcome.^41,42^ In our analysis, we found that patients treated in the methylprednisolone group had a significantly lower rate of decompressive hemicraniectomy compared with the placebo group. The results of this analysis suggest that methylprednisolone may be associated with a reduction in brain tissue edema and the occurrence of malignant brain edema. The protective mechanisms by which glucocorticoids reduce hemorrhagic transformation and cerebral edema progression due to cerebral infarction needs further study.

Pneumonia after stroke is associated with poor functional outcome or death.^43^ The trial’s finding indicated that intravenous methylprednisolone was associated with decreased incidence of pneumonia during hospitalizaiton. These findings are consistent with those reported by Roquilly et al,^44^ who found that a 7-day course of stress dose hydrocortisone for intubated trauma patients resulted in a lower risk of hospital-acquired pneumonia. This lower risk may be associated with the potential for low-dose glucocorticoids to exert favorable immunomodulatory effects, rather than leading to an immunosuppressive state^45^; however, the specific mechanisms require further investigation in the future.

### Limitations

This study has several limitations. First, this study is a post hoc secondary analysis of a randomized clinical trial; hence, our results are exploratory and hypothesis generating. The results were not adjusted for multiple comparisons. The sample size may not have been sufficient to test the null hypothesis of a randomized clinical trial and could have led to a type I error. Second, while the MARVEL trial randomization process was not stratified according to the occlusion site or time to randomization, we used the IPTW method to control for confounders. Third, we did not measure cerebral edema; hence, we do not know if the association of methylprednisolone with independent ambulation at 90 days was mediated by a reduction in cerebral edema. Fourth, as the MARVEL trial was conducted among a Chinese population, it is not known whether our results would be generalizable to other racial and ethnic populations. Fifth, the area under the curve value of the IPTW model was 0.60, indicating moderate discriminative ability. This could result from the balanced baseline characteristics, which, while ensuring comparability, may limit model performance. Sixth, the decision-making process for hemicraniectomy remains complex and is influenced by multiple postrandomization and postthrombectomy factors, warranting further investigation.

## Conclusions

In this post hoc secondary analysis of a randomized clinical trial of intravenous methylprednisolone vs placebo for patients with intracranial ICA occlusion undergoing EVT, we found that adjunct intravenous methylprednisolone was associated with independent ambulation at 90 days. In addition, methylprednisolone treatment was significantly associated with lower rates of sICH within 48 hours, pneumonia, and decompressive hemicraniectomy after EVT. These findings suggest that the use of intravenous methylprednisolone as an adjunct to EVT may hold promise as a treatment option for patients with AIS due to intracranial ICA occlusion.

## Notes

Supplement 1.
Trial Protocol and Statistical Analysis Plan

Supplement 2. **eMethods.** Inverse Probability of Treatment Weighting **eTable 1.** Heterogeneity of the Treatment Effect of Methylprednisolone vs Placebo in Patients With or Without Intracranial Carotid Occlusion **eTable 2.** Efficacy and Safety Outcomes of Intravenous Methylprednisolone vs Placebo Before Endovascular Thrombectomy of Patients With Intracranial Internal Carotid Artery Occlusion

Supplement 3.
Data Sharing Statement
