# Supplementary material for: Methylprednisolone as Adjunct to Thrombectomy for Acute Intracranial Internal Carotid Artery Occlusion Stroke: Post Hoc Secondary Analysis of the MARVEL Randomized Clinical Trial
Source: JAMA Netw Open. 2025 Feb 18;8(2):e2459945. doi: 10.1001/jamanetworkopen.2024.59945 (PMC11836765; doi:10.1001/jamanetworkopen.2024.59945)
Supplement: Supplement 1. — Trial Protocol and Statistical Analysis Plan [file jamanetwopen-e2459945-s001.pdf]

Supplement 1

MARVEL： Methylprednisolone for acute large vessel occlusion: a randomized double-blind, placebo-controlled trial in revascularization patients

This supplement contains the following items:

1. Original protocol (page1 to 95), final protocol (page96 to 187), summary of changes (page188 to 190).

Note: personal identifying information has been redacted from the protocol and SAP documents to comply with international privacy legislation.

Clinical study protocol

Efficacy and safety of intravenous methylprednisolone sodium succinate in acute ischemic stroke patients underwent endovascular treatment: a multicenter, randomized, double-blind trial.

Principal Investigators

Wenjie Zi, MD. and Qingwu Yang, Ph.D.

Xinqiao Hospital, Army Medical University, Chongqing, China

Prepared by:

Wenjie Zi, MD, Xinqiao Hospital, Army Medical University, Chongqing, China

Qingwu Yang, Ph.D, Xinqiao Hospital, Army Medical University, Chongqing, China

Protocol Version: 1.0

Date 2021 September 29

Table of Contents

1. Study Synopsis **8**

2. Flow chart and schedule of assessment **15**

2.1 Flow Chart 15

2.2. Schedule of Assessments 16

3. List of Abbreviations **18**

4. Background **20**

4.1 The burden of stroke 20

4.2 Treatment in acute ischemic stroke 20

4.3 Glucocorticoid in Stroke 22

5. Study Objectives **24**

6. Product characteristics of methylprednisolone **25**

6.1 Description of the investigational medicinal product 25

6.2 Indications and Usage 26

6.3 Contraindications 29

6.4 Warning 29

7. Organizational Structure **36**

7.1 Funding 37

7.2 Trials Boards and Committees 37

7.2.1 Steering Committee 37

7.2.2 Data Safety and Monitoring Board 37

7.2.3 Ethics Advisory Board 38

7.2.4 Imaging Core Laboratory 38

7.2.5 Clinical Events Committee 38

8. Design **39**

9. Population **39**

9.1 Inclusion criteria 39

9.2 Exclusion Criteria 40

9.3 Sample Size 41

10. Imaging Training 42

11. Randomization **42**

12. Blinding/Unblinding **43**

13. Study Treatment **44**

13.1 Formulation, Packaging, and Handling 44

13.2 Test Product 44

13.3 Comparator 44

13.4 Dosage, Administration, and Compliance 45

13.4.1 Methylprednisolone and its Placebo ... 45
13.4.2 Concomitant therapy ... 45
13.4.4 Drug Accountability ... 46

14. Study Schedule ... 46

14.1 Screening period (Visit V0, Randomization) ... 47
14.1.1 Visit V0 ... 47
14.1.2 Randomization (R) ... 48
14.2 Treatment period (Visits V1， V2) ... 48
14.2.1 Visit 1 ... 48
14.2.2 Visit 2 ... 49
14.3 Follow Up period (Visits V3, V4) ... 49
14.3.1 Visit 3 ... 50
14.3.3 Visit 4 ... 50

15. Endpoints of the Clinical Trial ... 50

15.1 Efficacy Analysis-Primary Endpoint ... 50
15.2 Efficacy Analysis-Secondary Endpoints ... 51
15.3 Safety Analysis-Primary Endpoint ... 51
15.3.1 Primary Safety Endpoints ... 51
15.3.2 Secondary Safety Endpoints ... 52

16. Assessment ... 52

16.1 Assessment of efficacy ... 53
16.1.1 The modified Rankin Scale (mRS) ... 53
16.1.2 The National Institutes of Health Stroke Scale (NIHSS) ... 54
16.1.3 European Quality Five Dimensions Five Level scale ... 55
16.2 Assessment of safety ... 55
16.2.1 Mortality at 90±7 days ... 55
16.2.2 Symptomatic Intracranial Haemorrhage ... 55
16.2.3 Any ICH within 48 hours ... 56
16.2.4 Proportion of patients with pneumonia ... 57
16.2.4 Proportion of patients with gastrointestinal bleeding ... 57
16.2.5 Adverse events ... 57
16.2.6 Serious Adverse Event ... 60
16.2.7 Adverse Event Recording and Follow Up ... 61
16.2.8 Serious Adverse Events Reporting ... 62

17. Study Discontinuation Criteria ... 63

17.1 Patient Discontinuation ... 63
17.2 Study Treatment Discontinuation ... 64
17.3 Study and Site Discontinuation ... 64

18. Statistical Analysis ... 65

18.1 Determination of sample size ... 65
18.2 Analysis population ... 66
18.2.1 Intention-to-Treat Population ... 66

18.2.2. Per-Protocol Population 67
18.2.3. Safety Population 68
18.2 Analysis of Efficacy–primary endpoint 68
18.3 Analysis of Efficacy – secondary endpoints 69
18.4 Safety analysis 69
18.5 Tolerability Analysis (adverse events) 70
18.5.1 Adverse Events 70
18.5.2 Serious Adverse Events 70
18.5.3 All Adverse Events 70
18.6 Adjustment for Covariates and Subgroup Analyses 71
18.7 Missing data handling 72

19. Ethical and regulatory consideration 72
19.1 General requirements and Considerations 72
19.2 Study monitoring and quality control 73
19.3 Informed Consent 74
19.4 Confidentiality 75
19.5 Liability and Insurance 75

20. Administrative procedures 75
20.1 Curriculum vitae 75
20.2 Secrecy agreement 76
20.3 Ownership of data and use of the study results 76
20.4 Protocol amendments 76

21. Data Retention 77

22. Study report and publications 77

25. Appendices 81
25.1 Appendix 1: mRS 81
25.2 Appendix 2: NIHSS 82
25.2 Appendix 3: EQ-5D 90

1. Study Synopsis

**Study Title**	MARVEL: Methylprednisolone for acute large vessel occlusion: a randomized double-blind, placebo-controlled trial in recanalization patients
**Study registry number**	**registry number:** ChiCTR2100051729
**Indication**	Acute Ischemic Stroke
**Study Centres**	About 80 stroke centres in China
**Number of Subjects**	1672 (836 subjects per group)
**Study Estimate Duration**	February 2022 until June 2023
**Study Objectives**	To test the safety and efficacy of adjunctive low-dose methylprednisolone sodium succinate therapy for the treatment of acute ischemic stroke patients within 24 hours of onset who underwent EVT.
**Study Design**	A multicentre, randomized, double-blind, placebo-controlled trial
**Study Population**	Patients with large vessels occlusive stroke within 24 hours that underwent endovascular treatment
**Inclusion Criteria**	● Age ≥ 18 years;<br>● The time from onset to randomization was within 24 hours;<br>● Anterior circulation ischemic stroke was preliminarily determined according to clinical symptoms or imaging examination;<br>● Baseline Alberta Stroke Program Early CT Score (ASPECTS) ≥ 5;<br>● Baseline National Institutes of Health Stroke Scale (NIHSS) ≥ 6<br>● Computed tomography angiography (CTA) / magnetic resonance angiography (MRA) /digital subtraction angiography (DSA) confirmed occlusion of intracranial segment of

	internal carotid artery and middle cerebral artery who decided to receive endovascular treatment.<br>Written informed consent signed by patients or their family members.
**Exclusion Criteria**	● Intracranial hemorrhage confirmed by cranial computed tomography (CT) or magnetic resonance imaging (MRI);<br>● mRS score ≥ 2 before onset;<br>● Pregnant or lactating women;<br>● Allergic to contrast agents;<br>● Allergic to glucocorticoids;<br>● Participating in other clinical trials;<br>● Systolic blood pressure > 185 mmHg or diastolic pressure > 110 mmHg, and oral antihypertensive drugs can not control;<br>● Genetic or acquired bleeding constitution, lack of anticoagulant factors; Or oral anticoagulants and INR > 1.7;<br>● Blood sugar < 2.8 mmol/L (50 mg/dl) or > 22.2 mmol/L (400 mg/dl), platelet < 90 x 10^9/L;<br>● The artery is tortuous and the thrombectomy device cannot reach the target vessel;<br>● Bleeding history (gastrointestinal and urinary tract bleeding) in recent 1 month;<br>● Chronic hemodialysis and severe renal insufficiency (glomerular filtration rate < 30 ml/min or serum creatinine > 220 umol/L [2.5 mg/dL]);<br>● Life expectancy due to any advanced disease < 6 months;<br>● Follow-up is not expected to be completed;<br>● Intracranial aneurysm and arteriovenous malformation;<br>● Brain tumors with imaging mass effect;

	Systemic infectious diseases.
**Study drug**	Methylprednisolone sodium succinate group: The box of the methylprednisolone group contains 12 bottles, each containing 40mg of methylprednisolone sodium succinate. Both the bottle and box are labelled as "Study Drug".<br><br>Placebo group: The box of the placebo group contains 12 bottles, each containing 40mg of placebo. Both the bottle and box are labelled as "Study Drug".
**Randomization**	Eligible patients will be consecutively randomized to treatment with either Methylprednisolone or Placebo group with a ratio of 1:1 by a web-based APP (Jinlingshu) on mobile phone or computer (https://jinlingshu.com/). Randomization will be stratified by participating centres permutation block size of 4.
**Treatments**	Patients will be assigned to receive either a placebo or methylprednisolone, with a dosage of 2mg/kg (based on estimated or actual weight if known, not exceeding a maximum dose of 160 mg) per day for a duration of 3 days. The initial study drug will be administered as soon as possible after randomisation. It is recommended that the initial study drug administrated before arterial access closure, but it should not be delayed more than 2 hours after arterial access closure.
**Consent**	Explicit written, signed informed consent from the subject or legally authorized representative will be obtained prior to any protocol specific procedures.
**Criteria for Evaluation-Efficacy**	Primary Efficacy Endpoint<br>● Reduced disability level (ordinal shift in mRS score) at 90 ±7 days;<br>Secondary Efficacy Endpoints<br>● Proportion of patients with mRS score 0 to 4 at 90 ±7 days;

	● Proportion of patients with mRS score 0 to 3 at 90 ±7 days;<br>● Proportion of patients with mRS score 0 to 2 at 90 ±7 days;<br>● Proportion of patients with mRS score 0 to 1 or returned to pre-stroke mobidity at 90 ±7 days;<br>● NIHSS score at 5-7 days or at early discharge;<br>Health-related quality of life [European Quality of Life Five-Dimension visual-analogue scale (EQ-5D VAS)] at 90 ±7 days.
**Criteria for Evaluation-Safety**	Primary Safety Endpoints<br>● Mortality at 90 ±7 days;<br>● Proportion of patients with symptomatic intracranial haemorrhage within 48 hours. Symptomatic intracranial haemorrhage will be adjudicated by an independent Imaging Core Laboratory according to the modified Heidelberg Bleeding Classification;<br>Secondary Safety Endpoints<br>● Proportion of patients with any radiologic intracranial haemorrhage within 48 hours;<br>● Proportion of patients with pneumonia;<br>● Proportion of patients with gastrointestinal haemorrhage within 7 days after EVT;<br>● Incidence of serious adverse events.<br>Incidence of any adverse events.
**Sample Size Calculation**	The current trial is designed detect a shift on the modified Rankin Scale score, which represents the global disability, to a lower score. The expected distribution of the modified Rankin Scale score, derived from the EVT for acute anterior circulation ischemic stroke (ACTUAL) registry and the Highly Effective Reperfusion evaluated in Multiple Endovascular Stroke Trials (HERMES) collaboration, is as follows: 7%, 20%, 16%, 11%, 13%, 8% and 25%.<br>The sample size was calculated using the cumulative proportion of patients with mRS 0-2. We assume a moderate effect of 7% absolute increase in the cumulative proportion of patients with mRS 0-2 in the intervention group, compared with controls, indicating an odds ratio

	(OR) of 1.33, which have substantially exceeds the minimal clinically important difference.10<br>In order to demonstrate the expected treatment effect with a type-1 error alpha =0.05 (two-tailed) and a power of 80% (beta = 20%). A sample size of n= 1588 patients (n=794 per treatment group) is required.<br>The intention-to-treat principle will be applied to the primary analysis and, therefore, to safeguard against dilution of the treatment effect associated with an approximate 5% non-adherence rate (due to loss to follow-up, consent withdrawal and other reasons), we initially planned to enrol n=1672 patients (n=836 per treatment group) for this study. This estimation was performed based on PASS (NCSS, LLC. Kaysville, Utah, USA) version 15.0.
**Statistical Methods**	All efficacy analyses will be conducted on data from all randomly assigned patients according to the intention-to-treat principle. All efforts will be made to minimize the amount of missing data. Sensitivity analyses based on different hypotheses about the missingness pattern of the primary outcome will be performed to test for the robustness of the primary analysis. Analyses will also be repeated according the per protocol principle.<br>Analysis of Efficacy - primary endpoint:<br>The primary efficacy endpoint is disability evaluated 90 (±7) days after randomization using the modified Rankin Scale (mRS) score. The primary treatment effect will be estimated using Common Odds Ratio or assumption free method. Covariable were age, baseline NIHSS score, pre-stroke mRS, baseline ASPECTS score, use of intravenous thrombolysis, time from onset to randomization, and occlusion location.<br>Analysis of Efficacy - Secondary endpoint:<br>Proportion of with mRS score 0 to 4 at 90 ±7 days and proportion of mRS score 0 to 3 at 90 ±7 days, proportion of patients with mRS score 0 to 2 at 90 ±7 days, proportion of mRS score 0 to 1 or return to pre-morbid mRS score at 90 days (for patients with mRS >1). Between-group

	differences will be tested using generalized linear models. The risk ratios and corresponding 95%CI will be provided.<br>NIHSS score at 5-7 days or at early discharge and Health-related quality of life (EQ-5D VAS). The results will be compared between two randomized treatment groups using GLM. The mean difference and corresponding 95%CI will be provided. Normality and variance homogeneity of residuals will be accessed graphically. The win ratio method will be used if normality assumption is seriously violated.<br>Analysis of Safety:<br>The safety analysis will be conducted on the Safety Population.<br>Mortality due to any cause at 90 ±7 days. The difference of mortality in two treatment groups will be tested using modified Poisson Regression. In addition, the Kaplan-Meier method will be used to assess the mortality. The log-rank test will be applied to compare the two treatment groups. Cox regression model will be employed to calculate the hazard ratio with 95%CI.<br>In addition to mortality due at 90 ±7 days, SICH within 48 hours is considered to be another primary safety outcome. SICH will be evaluated according to the modified Heidelberg Bleeding Classification. Besides, modified Poisson regression will be fitted to estimate the risk ratio associated with the treatment effect. The proportion of Any radiologic ICH within 48 hours between methylprednisolone and placebo groups will be tested in the same manner as SICH.<br>Proportion of patients with pneumonia and proportion of patients with gastrointestinal haemorrhage within 7 days after EVT will be explore and tested the same as SICH and any ICH.<br>Adverse events (AE) and serious adverse event (SAE) will be summarized and presented by treatment group for all patients in the Safety Population. The incidences will be compared via

	Fisher's exact test or chi-square test as appropriate.

2. Flow chart and schedule of assessment

2.1 Flow Chart

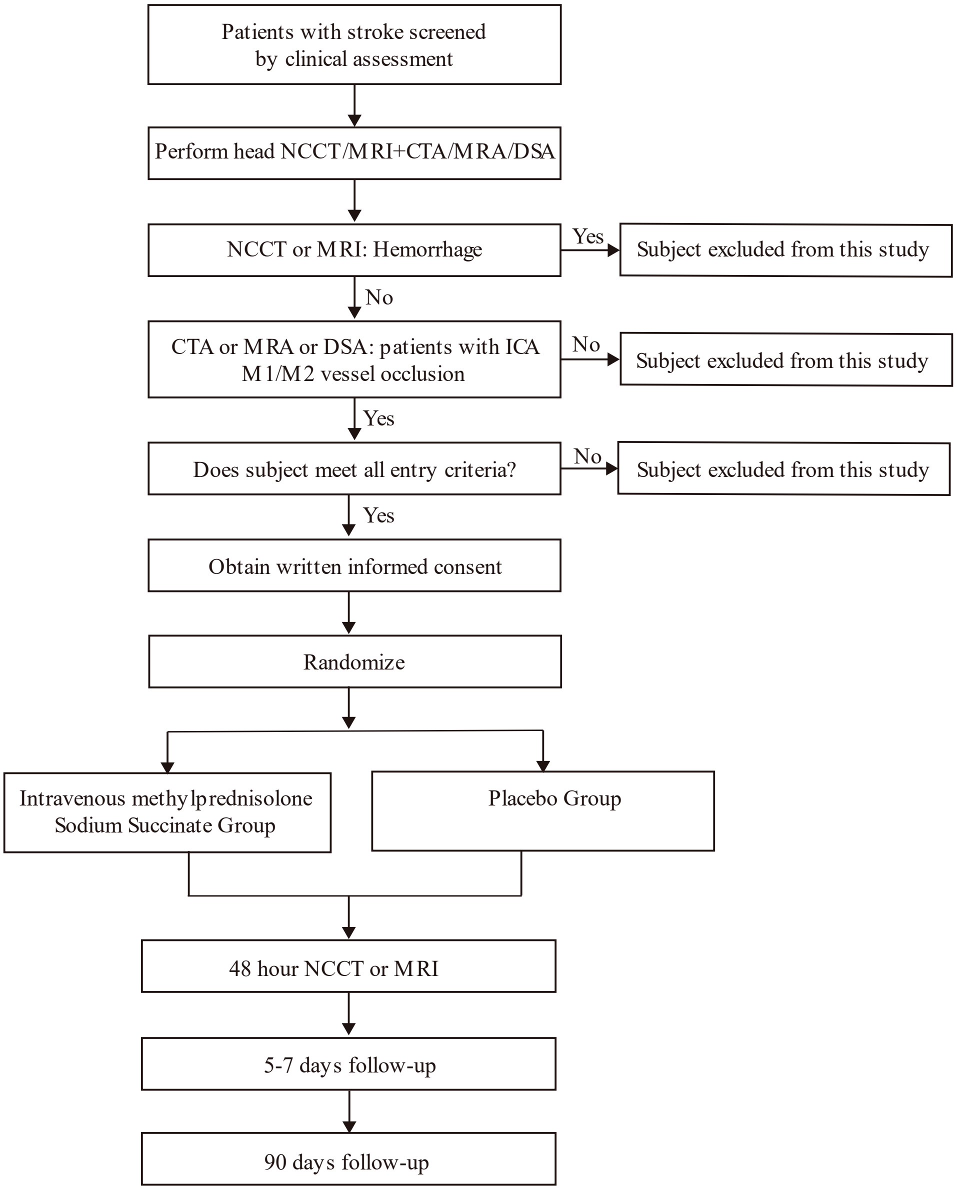

2.2. Schedule of Assessments

	**Visit (Time in hours / days from EVT =V1)**					
	Screening		Treatment		Follow Up	
	**V0**	**R**	**V1**[a] Study medication administration	**V2** (48-96h)	**V3**[b] (5-7d)	**V4** (90±7d)
Informed consent	X					
Demographic data	X					
Medical history	X					
Physical examination	X			X	X	
Pre-stroke mRS	X					
NIHSS	X				X	
mRS						X
EQ-5D						X
Previous medication	X					
Blood pressure and heart rate	X		X	X[e]	X	
Local laboratory result	X			X		
Pregnancy test[c]	X					

Brain CT/MRI plus CTA/MRA/DSA	X					
Brain CT/MRI scan[d]				X		
Inclusion / exclusion criteria	X					
Randomization		X				
Study medication			X			
Concomitant medication	X		X	X	X	X
12-lead ECG	X					
48h monitoring			X[a]			
Adverse events			X	X	X	X

V = Visit; R = Randomization; h = hour; d = day

[a] 48h monitoring in Unit or equivalent unit including repeated measurements of blood pressure, heart rate and body temperature

[b] or hospital discharge if < 5 days

[c] Mandatory for women of childbearing potential

[d] also to be performed in any case of neurological deterioration during the first 96h

[e] every hour

3. List of Abbreviations

AE	Adverse Event
AIS	Acute Ischemia Stroke
BI	Barthel Index
CRF	Case Report Form
CT	Computed Tomography
CTA	Computed Tomography Angiography
DSMB	Data Safety Monitoring Board
EAB	Ethics Advisory Board
ECG	Electrocardiogram
EQ-5D	European Quality Five-Dimension
EVT	Endovascular Therapy
GCP	Good Clinical Practice
GOS	Glasgow outcome scale
HBC	Heidelberg bleeding classification
ICH	Intracerebral Haemorrhage
ICH-GCP	International Conference on Harmonization-Good Clinical Practice
IRB	Institutional Review Board
ITT	Intention-to-Treat
IVT	Intravenous Thrombolysis
LVO	Large Vessel Occlusion
MARVEL	MARVEL: Methylprednisolone for acute large vessel occlusion: a

	randomized double-blind, placebo-controlled trial in revascularization patients
MedDRA	Medical Dictionary for Regulatory Activities
MR	Magnetic Resonance
MRA	Magnetic Resonance Angiography
mRS	modified Rankin Scale
NIHSS	National Institutes of Health Stroke Scale
NINDS	National Institute of Neurological Disorders and Stroke
PI	Principal Investigator
PP	Per-Protocol
REB	Research Ethics Board
RISS	Rapidly improving stroke symptoms
RR	Risk ratio
rt-PA	recombinant tissue Plasminogen Activator
SAB	Scientific Advisory Board
SAE	Serious Adverse Event
SAP	Statistical Analysis Plan
SICH	Symptomatic Intracerebral Haemorrhage
SITS-MOST	Safe Implementation of Thrombolysis in Stroke–Monitoring Study
SOC	System Organ Class

4. Background

4.1 The burden of stroke

Stroke remains the second-leading cause of death and the third-leading cause of disability combined in the world.[1-3] The estimated global cost of stroke is over US $721 billion (0.66% of the global GDP).[3] In the past 20 years, the burden increased substantially, with the bulk of the global stroke burden residing in lower-income and lower-middle-income countries.[3] Recently, the National Epidemiological Survey of Stroke in China (NESS-China) suggested that the current stroke prevalence (1115/100 000 [95%CI, 997–1233]) in China appears to be the highest among other low- to middle-income countries (range from 536 to 1040/100 000), but significantly lower than that observed in high-income countries (range from 2600 to 8000/100 000). [4]

4.2 Treatment in acute ischemic stroke

Stroke is normally classified as either ischemic or haemorrhagic stroke although 62.4% of cases belong to ischemic nature. Ischemic stroke is characterized by a high incidence, high disability rate, and high mortality rate, and has become the leading cause of death in China.[4] Currently, there are about 2.4 million new stroke patients and 1.1 million deaths in China each year, imposing a heavy burden on society and families.[4] Approximately 40% of acute ischemic strokes are caused acute large vessel occlusion (LVO), with anterior circulation LVO accounting for about 80% and posterior circulation LVO accounting for about 20%.[4]

Early restoration of blood flow and salvage of ischemic penumbra are the current theoretical basis of the treatment of AIS.[5,6] The current standard therapy for acute ischemic stroke including the intravenous thrombolysis and endovascular treatment.[6] Currently, the recanalization rate of acute anterior circulation LVO stroke has been improved to 80%~94.0% in the endovascular treatment era.[7]

However, the successful recanalization rates have not been completely translated into clinical benefits for AIS patients, and the proportion of patients with good outcome was less than 50%, which is clinically referred to as "Futile recanalization (FR)" or “Ineffective reperfusion”.[8,9] That is, successful recanalization of occluded vessels (defined as modified treatment in cerebral ischemia (mTICI) blood flow grade ⩾2b) does not lead to good functional outcomes (mRS score ⩾3 at 90 days). Moreover, reperfusion of the ischaemic tissue is not risk-free, often causes haemorrhagic transformation (HT), blood–brain barrier (BBB) disruption.[10-12] Besides, malignant brain oedema was a common complication that were associated with high mortality.[13,14]

Several mechanisms may contribute futile reperfusion and may be the potential treatment target for the futile reperfusion phenomenon: the non-reflow phenomenon, cerebral oedema, blood-brain barrier damage and inflammatory damage.[12]

Firstly, the "non-reflow" phenomenon refers to tissue hypoperfusion despite the timely or complete recanalization of an occluded artery.[15] This phenomenon is frequently observed in clinical practice, with a prevalence of more than half in cases of coronary

artery occlusion, and it serves as an independent predictor of adverse outcomes.[16] In the cerebral circulation, non-reflow has consistently been demonstrated in preclinical studies since first being described in 1968.[15] Non-reflow in the brain may involve post-ischemic swelling in endothelial cells, microvasculature occluded by platelet and neutrophils, interstitial oedema, and inflammatory reaction.[17] Recent experimental data further suggest that non-reflow is present during the first 24 hours after recanalization.[17] In clinical studies, the prevalence of the no-reflow phenomenon after endovascular treatment (EVT) in acute ischemic stroke was approximately 30%, and this number is expected to increase with the extension of EVT to a 24-hour time window.[17] Secondly, the endovascular treatment has a damage to BBB, and will lead to severe brain edema and haemorrhagic transformation that considerably contribute to neurological deterioration and death.[11] Thirdly, inflammatory mechanisms after stroke are now increasingly considered prime targets for stroke therapy since immune signals and their mediators can have both detrimental and beneficial effects at different stages of the disease process.[18,19]

Thus, due to the multi-faceted nature of the aforementioned mechanisms, single-target agents may not sufficiently intervene. Therefore, combinations of agents or multi-target agents may be necessary to address these mechanisms effectively.

4.3 Glucocorticoid in Stroke

In animal models, corticosteroids have shown the ability to reduce infarct size, regulate cerebral blood flow, enhance non-reflow phenomenon, stabilize the blood-brain barrier,

prevent angiogenic edema, and modulate immune response.[20-28], while none have been adopted through clinical trial success in acute ischemic stroke therapy.[29,30]

Several lessons are learned from prior studies. Firstly, those trials were performed in the era when reperfusion strategies were not well established. The pre-clinical study of corticosteroids has suggested that corticosteroid was effective in the transient middle cerebral artery occlusion(tMCAO) model but was ineffective in the permanent middle cerebral artery occlusion(pMCAO) model.[21] It suggested that reperfusion or not was an important condition for the effect of corticosteroids.[21,31-33] In past corticosteriod treatment trials, conducted before the advent of mechanical thrombectomy as a treatment option, it is likely that many patients failed to reperfuse; the candidate adjuvant treatments were tested in the more challenging setting of permanent rather than transient brain ischemia and thus failed to show benefit in clinical trials.[33] The STAIR (Stroke Treatment Academic Industry Roundtable) suggested testing the neuroprotective agents in the new endovascular treatment era when the reperfusion rate can be up to 90%. [31,34] [35] Secondly, most included patients with “presumed ischemic stroke” were treated in an era where reperfusion was not effective, and the sample size of patients were small in the previous trials. Only 8/24 published trials of corticosteroids in stroke were acceptable for further analysis, and these comprised woefully inadequate numbers of patients (only 466). Davis and Donnan suggested that steroid therapy for stroke be discarded prematurely. Thirdly, most trials used a high-dose and long duration of corticosteroid thus increasing the incidence of major gastrointestinal hemorrhage and infections. [29]

Therefore, there was a call to reignite the study of corticosteroids in stroke, especially in the reperfusion era. [35,36] One small sample size trial in China has studied the use of corticosteroids in the new endovascular treatment era and have yielded promising result that a short course and low-dose corticosteroid can improve the functional outcome, which was consistent with the preclinical study.[37] The surprising result indicated that low-dose and short-term corticosteroids may have an effect for patients with ischemic stroke in the new EVT era. A ray of hope comes to restudy the effect of corticosteroids in the EVT era. Trials conducted on patients who underwent endovascular treatment closely aligned with the primate model of transient middle cerebral artery occlusion, where the corticosteroid has demonstrated its efficacy. This is in contrast to the permanent middle cerebral artery occlusion model, where the corticosteroid has shown its benefit.[21]

As an intermediate-acting corticosteroid with its minimal side effects and its ability to effectively cross the blood-brain barrier, methylprednisolone is widely used in clinical practice. We propose a multicenter, prospective, randomized, double-blind, placebo-controlled trial to investigate the efficacy and safety of adjunctive methylprednisolone for patients underwent endovascular treatment. The main objective of this trial is to determine whether the early administration of methylprednisolone can improve the clinical outcomes of patients with acute anterior circulation large vessel occlusive stroke after successful recanalization.

5. Study Objectives

The purpose of MARVEL trial is to investigate the efficacy and safety of early adjunctive methylprednisolone in AIS patients underwent EVT in a multicenter, randomized, double-blind, placebo-controlled trial. The findings of MARVEL are likely to have a direct impact on clinical practice. In case of a positive result, this will provide evidence for an effective, safe, and affordable treatment in the new EVT era. Additionally, this trial will contribute high-quality evidence on the use of corticosteroids in stroke through a large-scale, multicenter trial, addressing existing gaps in current knowledge.

6. Product characteristics of methylprednisolone

The following is the summary of product characteristics for methylprednisolone provided by the Chongqing Lummy Pharmaceutical Co., Ltd., Chongqing, China.

6.1 Description of the investigational medicinal product

Methylprednisolone sodium succinate, is the sodium succinate ester of methylpred-nisolone, and it occurs as a white, or nearly white, odorless hygroscopic, amorphous solid. It is very soluble in water and in alcohol; it is insoluble in chloroform and is very slightly soluble in acetone.

The chemical name for methylprednisolone sodium succinate is pregna-1,4-diene-3,20dione,21-(3-carboxy-1-oxopropoxy)-11,17-dihydroxy-6-methyl-monosodium salt, (6α, 11β), and the molecular weight is 496.53. The structural formula is represented below:

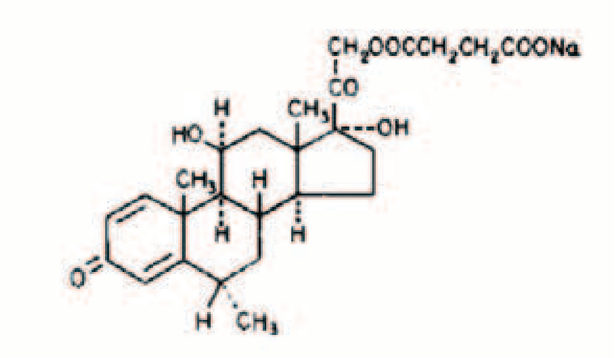

Methylprednisolone sodium succinate is soluble in water; it may be administered in a small volume of diluent and is well suited for intravenous use in situations where high blood levels of methylprednisolone are required rapidly.

CLINICAL PHARMACOLOGY

Glucocorticoids, naturally occurring and synthetic, are adrenocortical steroids that are readily absorbed from the gastrointestinal tract. Naturally occurring glucocorticoids (hydrocortisone and cortisone), which also have salt-retaining properties, are used as replacement therapy in adrenocortical deficiency states. Their synthetic analogs are primarily used for their potent anti-inflammatory effects in disorders of many organ systems. Glucocorticoids cause profound and varied metabolic effects. In addition, they modify the body's immune responses to diverse stimuli. Methylprednisolone is a potent anti-inflammatory steroid with greater anti-inflammatory potency than prednisolone and even less tendency than prednisolone to induce sodium and water retention. Methylprednisolone sodium succinate has the same metabolic and anti-inflammatory actions as methylprednisolone.

6.2 Indications and Usage

When oral therapy is not feasible, and the strength, dosage form, and route of administration of the drug reasonably lend the preparation to the treatment of the condition, the intravenous or intramuscular use of Methylprednisolone is indicated as follows:

Allergic states: Control of severe or incapacitating allergic conditions intractable to adequate trials of conventional treatment in asthma, atopic dermatitis, contact dermatitis, drug hypersensitivity reactions, perennial or seasonal allergic rhinitis, serum sickness, transfusion reactions.

Dermatologic diseases: Bullous dermatitis herpetiformis, exfoliative erythroderma, mycosis fungoides, pemphigus, severe erythema multiforme (Stevens-Johnson syndrome).

Endocrine disorders: Primary or secondary adrenocortical insufficiency (hydrocortisone or cortisone is the drug of choice; synthetic analogs may be used in conjunction with mineralocorticoids where applicable; in infancy, mineralocorticoid supplementation is of particular importance), congenital adrenal hyperplasia, hypercalcemia associated with cancer, nonsuppurative thyroiditis.

Gastrointestinal diseases: To tide the patient over a critical period of the disease in regional enteritis (systemic therapy) and ulcerative colitis. Hematologic disorders: Acquired (autoimmune) hemolytic anemia, congenital (erythroid) hypoplastic anemia (Diamond-Blackfan anemia), idiopathic thrombocytopenic purpura in adults

(intravenous administration only; intramuscular administration is contraindicated), pure red cell aplasia, selected cases of secondary thrombocytopenia.

Miscellaneous: Trichinosis with neurologic or myocardial involvement, tuberculous meningitis with subarachnoid block or impending block when used concurrently with appropriate antituberculous chemotherapy.

Neoplastic diseases: For the palliative management of leukemias and lymphomas.

Nervous System: Acute exacerbations of multiple sclerosis; cerebral edema associated with primary or metastatic brain tumor, or craniotomy.

Ophthalmic diseases: Sympathetic ophthalmia, uveitis and ocular inflammatory conditions unresponsive to topical corticosteroids. Renal diseases: To induce diuresis or remission of proteinuria in idiopathic nephrotic syndrome or that due to lupus erythematosus.

Respiratory diseases: Berylliosis, fulminating or disseminated pulmonary tuberculosis when used concurrently with appropriate antituberculous chemotherapy, idiopathic eosinophilic pneumonias, symptomatic sarcoidosis.

Rheumatic disorders: As adjunctive therapy for short-term administration (to tide the patient over an acute episode or exacerbation) in acute gouty arthritis; acute rheumatic carditis; ankylosing spondylitis; psoriatic arthritis; rheumatoid arthritis, including juvenile rheumatoid arthritis (selected cases may require low-dose maintenance therapy). For the treatment of dermatomyositis, temporal arteritis, polymyositis, and systemic lupus erythematosus.

6.3 Contraindications

The methylprednisolone sodium succinate is contraindicated:

- in systemic fungal infections and patients with known hypersensitivity to the product and its constituents.
- for intrathecal administration. Reports of severe medical events have been associated with this route of administration.

6.4 Warning

Serious Neurologic Adverse Reactions with Epidural Administration

Serious neurologic events, some resulting in death, have been reported with epidural injection of corticosteroids. Specific events reported include, but are not limited to, spinal cord infarction, paraplegia, quadriplegia, cortical blindness, and stroke. These serious neurologic events have been reported with and without use of fluoroscopy. The safety and effectiveness of epidural administration of corticosteroids have not been established, and corticosteroids are not approved for this use.

GENERAL

Increased dosage of rapidly acting corticosteroids is indicated in patients on corticosteroid therapy who are subjected to any unusual stress before, during, and after the stressful situation.

Results from one multicenter, randomized, placebo-controlled study with methylprednisolone hemisuccinate, an intravenous corticosteroid, showed an increase in early (at 2 weeks) and late (at 6 months) mortality in patients with cranial trauma

who were determined not to have other clear indications for corticosteroid treatment. High doses of systemic corticosteroids, should not be used for the treatment of traumatic brain injury.

Cardio-renal

Average and large doses of corticosteroids can cause elevation of blood pressure, salt and water retention, and increased excretion of potassium. These effects are less likely to occur with the synthetic derivatives except when used in large doses. Dietary salt restriction and potassium supplementation may be necessary. All corticosteroids increase calcium excretion. Literature reports suggest an apparent association between the use of corticosteroids and left ventricular free wall rupture after a recent myocardial infarction; therefore, therapy with corticosteroids should be used with great caution in these patients.

Endocrine

Hypothalamic-pituitary adrenal (HPA) axis suppression, Cushing's syndrome, and hyperglycemia. Monitor patients for these conditions with chronic use. Corticosteroids can produce reversible HPA axis suppression with the potential for glucocorticosteroid insufficiency after withdrawal of treatment. Drug induced secondary adrenocortical insufficiency may be minimized by gradual reduction of dosage. This type of relative insufficiency may persist for months after discontinuation of therapy; therefore, in any situation of stress occurring during that period, hormone therapy should be reinstituted.

Drug-Induced Liver Injury

Rarely, high doses of cyclically pulsed intravenous methylprednisolone (usually for the treatment of exacerbations of multiple sclerosis at doses of 1 gram/day) can induce a toxic form of acute hepatitis. The time to onset of this form of steroid-induced liver injury can be several weeks or longer. Resolution has been observed after discontinuation of treatment. However, serious liver injury can occur, sometimes resulting in acute liver failure and death. Discontinue intravenous methylprednisolone if toxic hepatitis occurs. Since recurrence has occurred after re-challenge, avoid use of high dose intravenous methylprednisolone in patients with a history of toxic hepatitis caused by methylprednisolone.

Infections

General

Patients who are on corticosteroids are more susceptible to infections than are healthy individuals. There may be decreased resistance and inability to localize infection when corticosteroids are used. Infections with any pathogen (viral, bacterial, fungal, protozoan, or helminthic) in any location of the body may be associated with the use of corticosteroids alone or in combination with other immunosuppressive agents. These infections may be mild, but can be severe and at times fatal. With increasing doses of corticosteroids, the rate of occurrence of infectious complications increases. Corticosteroids may also mask some signs of current infection. Do not use intraarticularly, intrabursally or for intratendinous administration for local effect in the presence of acute local infection. A study has failed to establish the efficacy of methylprednisolone sodium succinate in the treatment of sepsis syndrome and septic

shock. The study also suggests that treatment of these conditions with methylprednisolone sodium succinate may increase the risk of mortality in certain patients (i.e., patients with elevated serum creatinine levels or patients who develop secondary infections after methylprednisolone sodium succinate).

Fungal infections

Corticosteroids may exacerbate systemic fungal infections and therefore should not be used in the presence of such infections unless they are needed to control drug reactions. There have been cases reported in which concomitant use of amphotericin B and hydrocortisone was followed by cardiac enlargement and congestive heart failure (see CONTRAINDICATIONS and PRECAUTIONS, Drug Interactions, Amphotericin B injection and potassium-depleting agents). Special pathogens Latent disease may be activated or there may be an exacerbation of intercurrent infections due to pathogens, including those caused by Amoeba, Candida, Cryptococcus, Mycobacterium, Nocardia, Pneumocystis, Toxoplasma. It is recommended that latent amebiasis or active amebiasis be ruled out before initiating corticosteroid therapy in any patient who has spent time in the tropics or in any patient with unexplained diarrhea. Similarly, corticosteroids should be used with great care in patients with known or suspected Strongyloides (threadworm) infestation. In such patients, corticosteroid-induced immunosuppression may lead to Strongyloides hyperinfection and dissemination with widespread larval migration, often accompanied by severe enterocolitis and potentially fatal gram-negative septicemia. Corticosteroids should not be used in cerebral malaria. There is currently no evidence of benefit from

steroids in this condition.

Tuberculosis

The use of corticosteroids in active tuberculosis should be restricted to those cases of fulminating or disseminated tuberculosis in which the corticosteroid is used for the management of the disease in conjunction with appropriate antituberculous regimen. If corticosteroids are indicated in patients with latent tuberculosis or tuberculin reactivity, close observation is necessary as reactivation of the disease may occur. During prolonged corticosteroid therapy, these patients should receive chemoprophylaxis.

Vaccination

Administration of live or live, attenuated vaccines is contraindicated in patients receiving immunosuppressive doses of corticosteroids. Killed or inactivated vaccines may be administered. However, the response to such vaccines cannot be predicted. Immunization procedures may be undertaken in patients receiving corticosteroids as replacement therapy, e.g., for Addison’s disease.

Viral infections

Chicken pox and measles can have a more serious or even fatal course in pediatric and adult patients on corticosteroids. In pediatric and adult patients who have not had these diseases, particular care should be taken to avoid exposure. The contribution of the underlying disease and/or prior corticosteroid treatment to the risk is also not known. If exposed to chicken pox, prophylaxis with varicella zoster immune globulin (VZIG) may be indicated. If exposed to measles, prophylaxis with immunoglobulin

(IG) may be indicated. (See the respective package inserts for complete VZIG and IG prescribing information.) If chicken pox develops, treatment with antiviral agents should be considered.

Neurologic

Reports of severe medical events have been associated with the intrathecal route of administration (see ADVERSE REACTIONS, Gastrointestinal and Neurologic/Psychiatric).

Ophthalmic

Use of corticosteroids may produce posterior subcapsular cataracts, glaucoma with possible damage to the optic nerves, and may enhance the establishment of secondary ocular infections due to bacteria, fungi, or viruses. The use of oral corticosteroids is not recommended in the treatment of optineuritis and may lead to an increase in the risk of new episodes. Corticosteroids should be used cautiously in patients with ocular herpes simplex because of corneal perforation. Corticosteroids should not be used in active ocular herpes simplex.

PRECAUTIONS

General

This product, like many other steroid formulations, is sensitive to heat. Therefore, it should not be autoclaved when it is desirable to sterilize the exterior of the vial. The lowest possible dose of corticosteroid should be used to control the condition under treatment. When reduction in dosage is possible, the reduction should be gradual. Since complications of treatment with glucocorticoids are dependent on the size of the

dose and the duration of treatment, a risk/benefit decision must be made in each individual case as to dose and duration of treatment and as to whether daily or intermittent therapy should be used. Kaposi's sarcoma has been reported to occur in patients receiving corticosteroid therapy, most often for chronic conditions. Discontinuation of corticosteroids may result in clinical improvement.

Cardio-renal

As sodium retention with resultant edema and potassium loss may occur in patients receiving corticosteroids, these agents should be used with caution in patients with congestive heart failure, hypertension, or renal insufficiency.

Endocrine

Drug-induced secondary adrenocortical insufficiency may be minimized by gradual reduction of dosage. This type of relative insufficiency may persist for months after discontinuation of therapy; therefore, in any situation of stress occurring during that period, hormone therapy should be reinstituted. Metabolic clearance of corticosteroids is decreased in hypothyroid patients and increased in hyperthyroid patients. Changes in thyroid status of the patient may necessitate adjustment in dosage.

Gastrointestinal

Steroids should be used with caution in active or latent peptic ulcers, diverticulitis, fresh intestinal anastomoses, and nonspecific ulcerative colitis, since they may increase the risk of a perforation. Signs of peritoneal irritation following gastrointestinal perforation in patients receiving corticosteroids may be minimal or absent. There is an enhanced effect due to decreased metabolism of corticosteroids in

patients with cirrhosis.

Musculoskeletal

Corticosteroids decrease bone formation and increase bone resorption both through their effect on calcium regulation (i.e., decreasing absorption and increasing excretion) and inhibition of osteoblast function. This, together with a decrease in the protein matrix of the bone secondary to an increase in protein catabolism, and reduced sex hormone production, may lead to inhibition of bone growth in pediatric patients and the development of osteoporosis at any age. Special consideration should be given to patients at increased risk of osteoporosis (i.e., postmenopausal women) before initiating corticosteroid therapy. Local injection of a steroid into a previously infected site is not usually recommended.

Neurologic-Psychiatric

Although controlled clinical trials have shown corticosteroids to be effective in speeding the resolution of acute exacerbations of multiple sclerosis, they do not show that corticosteroids affect the ultimate outcome or natural history of the disease. The studies do show that relatively high doses of corticosteroids are necessary to demonstrate a significant effect. An acute myopathy has been observed with the use of high doses of corticosteroids, most often occurring in patients with disorders of neuromuscular transmission (e.g., myasthenia gravis), or in patients receiving concomitant therapy with neuromuscular blocking drugs (e.g., pancuronium). This acute myopathy is generalized, may involve ocular and respiratory.

7. Organizational Structure

7.1 Funding

MARVEL trial is an investigator-initiated study which is organized by the second affiliated hospital of the Third Military Medical University and will conduct in about 80 centres in China. MARVEL will be funded by the National Natural Science Foundation of China. Methylprednisolone and its placebo are manufactured and provided by Chongqing Lummy Pharmaceutical Co., Ltd., Chongqing, China. The funder had no involvement in the study design, data collection, analysis and interpretation, writing or decision to submit the paper.

7.2 Trials Boards and Committees

The following boards and institutions will assure the success of the clinical trial:

7.2.1 Steering Committee

The Steering Committee of the clinical trial will decide on the final protocol and oversee the trial.

7.2.2 Data Safety and Monitoring Board

The independent Date and Safety Monitoring Board (DSMB) will regularly monitor the safety of the trial and ensure the safety of patients at all stages of MARVEL. The independent DSMB will be composed of an experienced neurologist, a neuroradiologist, and a biostatistician, who are not participants of the MARVEL consortium and not involved in the clinical trial in any other way. The DSMB may at

any time propose to revise the clinical trial protocol or terminate the trial in case of safety concerns.

7.2.3 Ethics Advisory Board

The Ethics Advisory Board (EAB) will supervise the trial and ensure that the trial is conducted in accordance with the ICH-GCP guidelines and international and China legislation. The EAB may at any time propose to revise the clinical trial protocol or terminate the trial in case of ethical concerns.

7.2.4 Imaging Core Laboratory

Centralized imaging core laboratories will centrally blinded review all images and provide reference judgements for the definition of the per protocol population and intracranial haemorrhages. CT/MR and angiographic images will be independently reviewed by two independent central imaging core laboratories respectively. Imaging Core Laboratory will be independent to ensure it is blinded to the treatment allocation.

7.2.5 Clinical Events Committee

The Clinical Events Committee will be comprised of three expert physicians independent of the investigational sites. This committee will validate all the complications that occur over the course of the study and categorize each for severity and relatedness according to the definition in the AE section. The Clinical Events Committee can request any additional source information and images supporting the AEs to assist with the adjudication.

8. Design

MARVEL is a multicenter, randomized, double-blind, placebo-controlled trial. Patients will be randomized 1:1 to either Methylprednisolone Group or Placebo Group. The study has been registered at Chinese Clinical Trial Registry (www.chictr.org.cn, unique identifier ChiCTR2100051729).

9. Population

The patients for this trial will be recruited from AIS patients with large vessel occlusion within 24 hours from last known well.

9.1 Inclusion criteria

- Age >= 18 years;
- The time from onset to randomization was within 24 hours;
- Anterior circulation ischemic stroke was preliminarily determined according to clinical symptoms or imaging examination;
- Baseline National Institutes of Health Stroke Scale (NIHSS) ≥ 6
- Baseline Alberta Stroke Program Early CT Score (ASPECTS)>= 5;
- Computed tomography angiography (CTA) /magnetic resonance angiography (MRA) /digital subtraction angiography (DSA) confirmed occlusion of intracranial segment of internal carotid artery and middle cerebral artery, and decided to undergo endovascular therapy;

- Written informed consent signed by patients or their family members.

9.2 Exclusion Criteria

Patients meeting any of the following criteria will be excluded from study enrolment.

- Intracranial hemorrhage confirmed by cranial computed tomography (CT) or magnetic resonance imaging (MRI);
- mRS score >= 2 before onset;
- Pregnant or lactating women;
- Allergic to contrast agents;
- Allergic to glucocorticoids;
- Participating in other clinical trials;
- Systolic blood pressure > 185 mmHg or diastolic pressure > 110 mmHg, and oral antihypertensive drugs can not control;
- Genetic or acquired bleeding constitution, lack of anticoagulant factors; Or oral anticoagulants and INR > 1.7;
- Blood sugar < 2.8 mmol/L (50 mg/dl) or > 22.2 mmol/L (400 mg/dl), platelet < 90 x 10^9/L;
- The artery is tortuous so that the thrombectomy device cannot reach the target vessel;
- Bleeding history (gastrointestinal and urinary tract bleeding) in recent 1 month;

- Chronic hemodialysis and severe renal insufficiency (glomerular filtration rate < 30 ml/min or serum creatinine > 220 umol/L [2.5 mg/ dL]);
- Life expectancy due to any advanced disease < 6 months;
- Follow-up is not expected to be completed;
- Intracranial aneurysm and arteriovenous malformation;
- Brain tumors with imaging mass effect;
- Severe systemic infectious diseases.

9.3 Sample Size

The current trial is designed detect a shift on the modified Rankin Scale score, which represents the global disability, to a lower score. The expected distribution of the modified Rankin Scale score, derived from the EVT for acute anterior circulation ischemic stroke (ACTUAL) registry[38] and the Highly Effective Reperfusion evaluated in Multiple Endovascular Stroke Trials (HERMES) collaboration[7], is as follows: 7%, 20%, 16%, 11%, 13%, 8% and 25%.

The sample size was calculated using the cumulative proportion of patients with mRS 0-2. We assume a moderate effect of 7% absolute increase in the cumulative proportion of patients with mRS 0-2 in the intervention group, compared with controls, indicating an odds ratio (OR) of 1.33, which have substantially exceeds the minimal clinically important difference.[39,40] In order to demonstrate the expected

treatment effect with a type-1 error alpha =0.05 (two-tailed) and a power of 80% (beta = 20%). A sample size of n= 1588 patients (n=794 per treatment group) is required.

The intention-to-treat principle will be applied to the primary analysis and, therefore, to safeguard against dilution of the treatment effect associated with an approximate 5% non-adherence rate (due to loss to follow-up, consent withdrawal and other reasons), we initially planned to enrol n=1672 patients (n=836 per treatment group) for this study. This estimation was performed based on PASS (NCSS, LLC. Kaysville, Utah, USA) version 15.0.

10. Imaging Training

Investigators involved in image reading within the trial will participate in a standardized image reading training before study start.

11. Randomization

Randomization will be done by a web-based APP (Jinlingshu) on mobile phone or computer (https://jinlingshu.com/). The automated system will assign an appropriate set of study medication to each patient. Eligible patients are randomized to treatment with either intravenous methylprednisolone or placebo with a ratio of 1:1. Randomization will be stratified by participating centre with permutation block size of 4. Randomization will be completely concealed by having both web-based real-time allocation and identical appearance of methylprednisolone sodium succinate and placebo bottles. (All bottles will have a unique number. Subjects will be assigned a

random serial number according to the time they were enrolled, and corresponding masked medications will be provided).

The randomization list will be prepared by the independent statistical centre using SAS 9.4. It will only be sent to the data coordination centre responsible for central randomization and data management, the pharmacy centre responsible for treatment packaging.

12. Blinding/Unblinding

Both patient and investigator are blinded to treatment assignment. If there is a clinical situation that the investigator believes it is necessary to unblind for the safety of patients, the medical monitor must be immediately notified to discuss the intended unblinding. Premature breaking of the blind should be restricted to the setting where identification of the type of treatment is critical for adequate treatment of the patient. All unblinded patients will remain in the study and complete all follow-up visits.

If the site principal investigator wants to know the identity of the study drug for any other reason, he or she must call the medical monitor. Unless the circumstances are deemed necessary by site principal investigator, treatment codes should not be broken. The site principal investigator should document and make an explanation for any premature unblinding.

For the purpose of regulatory reporting, if required by the local health authorities, treatment codes could be broken for all serious, unexpected suspected adverse reactions that are deemed by the investigator to be related to study drug.

13. Study Treatment

13.1 Formulation, Packaging, and Handling

Methylprednisolone sodium succinate and its placebo are manufactured by Lummy Pharmaceutical Group Co., Ltd., Chongqing, China. All study medication will be manufactured, tested, released, and shipped according to Good Manufacturing Practice guidelines. Labelling and packaging of study medication will be conducted according to Good Clinical Practice and Good Manufacturing Practice guidelines, and any national regulatory requirements.

Methylprednisolone sodium succinate and its corresponding placebos are provided in numbered and are visually identical, except for a unique number. Methylprednisolone sodium succinate and it’s placebo will be packed in glass bottle of identical appearance. Each kit has a unique identification number and will be stored in a safe location at room temperature on the clinical site with limited access.

13.2 Test Product

Each kit of the methylprednisolone group contains l2 bottles, and each bottles contains 40mg methylprednisolone sodium succinate, labelled for “Study Drug” and “clinical trial use only”). Each kit will be stored in a safe location at room temperature (25°C with excursions permitted between 15°C−30°C) on the clinical site with restricted access.

13.3 Comparator

Each kit of the placebo group contains l2 bottles, and each bottles contains 40mg placebo, labelled for "Study Drug" and "clinical trial use only"). Each kit will be stored in a safe location at room temperature (25°C with excursions permitted between 15°C−30°C) on the clinical site with restricted access.

13.4 Dosage, Administration, and Compliance

Eligible patients will be randomly assigned a number corresponding to a blinded sealed medication kit that sent to each patient. It is recommended to start using the study drug within 15 minutes after randomization.

13.4.1 Methylprednisolone and its Placebo

Patients will be assigned to receive either a placebo or methylprednisolone, with a dosage of 2mg/kg (based on estimated or actual weight if known, not exceeding a maximum dose of 160 mg) per day for a duration of 3 days. The initial study drug will be administered as soon as possible after randomisation. It is recommended that the initial study drug administrated before arterial access closure, but it should not be delayed more than 2 hours after arterial access closure. All patients will be treated according to the current Chinese Stroke Association guidelines for clinical management of cerebrovascular disorders[41]. Risk factors such as diabetes mellitus, hypertension, hyperlipidaemia, hyperhomocysteinaemia, obesity, drinking and smoking should be managed appropriately.

13.4.2 Concomitant therapy

Administration of any other (intravenous or oral) corticosteroids (E.g.,

Hydrocortisone) is not allowed during the first 72 hours post randomization. Gastrointestinal prophylaxis and hyperglycemia treatment will be administered per standard local protocols. Any concomitant medication within one week prior to screening through the study completion/discontinuation visit should be documented in the Concomitant Medications Case Report Form.

13.4.4 Drug Accountability

The investigational sites will be provided with sufficient amounts of investigational medicinal products. The investigational medicinal products must not be used outside the study protocol. The investigator or authorized staff is obliged to acknowledge receipt of the study medication to confirm the content and shipping temperature. Any damaged shipment of study medications will be replaced. The study drug will be disposed of in accordance with the standard operating procedures at the study site, or returned to the sponsor with appropriate documents. The investigational site's method of study medication destruction must be agreed to by the Sponsor.

Before destroying any study medications, the investigational site must obtain the written authorization from the sponsor and document the destruction of study medications in an appropriate form. Accurate records of receipt, dispensation, return and disposition of all study medication during the study should be documented on the drug inventory log.

14. Study Schedule

The schedule of assessments conducted during the study is shown in Part 2 of Chapter

2. Flow Chart and Schedule of Assessments. The study includes three periods: Screening (V0), treatment (V1, V2) and Follow up (V3, V4).

14.1 Screening period (Visit V0, Randomization)

14.1.1 Visit V0

For each eligible patient, written informed consent for participation in the study will be obtained from the patient or the patient's legal representative prior to the performance of any protocol-related investigation. Once written informed consent is obtained, the results of the following standard of care assessments will be used for the study. The investigator will record the details of all patients consented to confirm eligibility and record the reasons for screening failure.

- Demographics
- Medical history
- Physical examination
- Determination of the pre-stroke mRS via interview of the patient or the patient's kin
- NIHSS score (Note: performed by an NIHSS-certified practitioner)
- Collection of information on previous medications
- Measurement of blood pressure and heart rate
- Pregnancy test (urine test, in non-menopausal women)

- Laboratory tests
- A 12-lead ECG
- Non-contrast CT scan or MRI scan and CTA, MRA, or DSA
- Concomitant medications
- Checking of inclusion and exclusion criteria

14.1.2 Randomization (R)

If all inclusion criteria are met and no exclusion criteria are present, randomization will be performed.

14.2 Treatment period (Visits V1， V2)

- After randomization, the treatment period starts, including study medication (V1) and post-treatment clinical examination (V2).

14.2.1 Visit 1

Before administration of the study medication, measurement of blood pressure and heart rate have to be performed. The study medication will be administered as specified for intravenous methylprednisolone. During the administration of study medication, the following assessments will be made:

- Measurement of blood pressure and heart rate every hour.
- Documentation of any concomitant medication.
- Assessment of Adverse Event.

- In any case of significant neurological deterioration judged by the investigator, an assessment of the neurological deficit and another non-contrast cranial CT scan or MRI scan have to be performed to determine whether intracranial haemorrhage occurs.
- The 48h monitoring should be performed in Stroke Unit or equivalent unit.

14.2.2 Visit 2

- Visit 2 comprises the clinical examination and a second non-contrast cranial CT or MRI scan which is performed to diagnose intracranial haemorrhage. Visit 2 will be done 48-96h after the administration of study medication. The following assessments will be performed:
- Physical examination
- Assessment of the neurological deficit by the NIHSS
- Measurement of blood pressure and heart rate
- Laboratory tests
- Non-contrast cranial CT or MRI scan
- Documentation of any concomitant medication
- Assessment of Adverse Event

14.3 Follow Up period (Visits V3, V4)

The Follow Up period comprises the period of the subacute stage (day 5-7 or hospital

discharge) in Visit 3， and the final follow up examination 90±7 days in Visit 4.

14.3.1 Visit 3

The following assessments will be performed:

- Physical examination
- Assessment of the neurological deficit by the NIHSS
- Measurement of blood pressure and heart rate
- Documentation of any concomitant medication
- Assessment of Adverse Event

14.3.3 Visit 4

The following assessments will be performed:

- Physical examination (If the patients return to local hospital)
- Assessment of the neurological deficit by the NIHSS
- Assessment of functional status by the mRS
- Assessment of functional health status and quality of life by the EQ-5D scale
- Documentation of any concomitant medication
- Assessment of Adverse Event

15. Endpoints of the Clinical Trial

15.1 Efficacy Analysis-Primary Endpoint

The primary outcome was the ordinal mRS score, a global measure of disability, comprises of seven grades ranging from 0 (no symptoms) to 6 (death).

15.2 Efficacy Analysis-Secondary Endpoints

- Proportion of patients with mRS score 0 to 4 at 90±7 days;
- Proportion of patients with mRS score 0 to 3 at 90±7 days;
- Proportion of patients with mRS score 0 to 2 at 90±7 days
- Proportion of patients with mRS score 0 to 1(or returned to pre-stroke modified Rankin scale score) at 90±7 days
- NIHSS score at 5-7 days after EVT or at early discharge;
- European Quality Five-Dimension scale score at 90 days.

15.3 Safety Analysis-Primary Endpoint

15.3.1 Primary Safety Endpoints

- Mortality due to any cause at 90 (±7) days;
- Proportion of patients with symptomatic intracranial haemorrhage within 48 hours after endovascular treatmen. Symptomatic intracranial haemorrhage will be adjudicated by an independent Imaging Core Laboratory and the Clinical Events Committee according to the modified Heidelberg Bleeding Classification, and is diagnosed based on a combination of: 1) clinical deterioration, 2) imaging findings, and 3) causal relatedness assessment. Clinical deterioration is defined as any of the following conditions: 1) NIHSS score increased more than 4 points than that

immediately before worsening; 2) NIHSS score increased more than 2 points in one category; 3) Deterioration led to intubation, hemicraniectomy, external ventricular drain placement or any other major interventions. The imaging criteria are presence of parenchymal hematoma type 2 (PH2), parenchymal hematoma type 1 (PH1), remote intracerebral haemorrhage (RIH), subarachnoid haemorrhage (SAH), intraventricular haemorrhage (IVH), or subdural haemorrhage. When clinical deterioration and PH2 co-occur, the event is automatically categorized as SICH. When clinical deterioration and PH1/RIH/SAH/IVH/SDH co-occur, the event is deemed SICH if it is judged that the haemorrhage contributed substantially to the clinical worsening. When clinical deterioration occurs only with haemorrhagic infarction type 1 or 2 (HI1 or HI2), the event is automatically classified as not SICH. ICH.

15.3.2 Secondary Safety Endpoints

- Proportion of patients with any radiologic intracranial haemorrhage within 48 hours after treatment;
- Proportion of patients with pneumonia;
- Proportion of patients with gastrointestinal haemorrhage within 7 days after EVT;
- Incidence of serious adverse events.
- Incidence of any adverse events.

16. Assessment

16.1 Assessment of efficacy

16.1.1 The modified Rankin Scale (mRS)

The mRS score is a valid and reliable clinician-reported measure of global disability that has been widely applied for evaluating recovery from stroke. It is a scale used to measure functional recovery (the degree of disability or dependence in daily activities) of people who have suffered a stroke[42,43]. mRS scores range from 0 to 6, with 0 indicating no residual symptoms; 5 indicating bedbound, requiring constant care; and 6 indicating death. The mRS score will be obtained at

Day 90. Premorbid mRS status will also be obtained retrospectively and reported on the 24 Hours CRF page. In this trial, we will keep video and voice recording versions of mRS score except those who die, unable, or refuse to take a video. For mRS score at Day 90, the mRS score will be assessed by two independent certified neurologists in a blinded manner with the video combined with voice record. For those who decline to participate in a video recording, the outcomes will be determined in person by site neurologists blinded to the treatment assignment, and keep a chat record with site neurologists. Disagreements are resolved by consensus.

The mRS will only be scored by those who have been trained and certified to use this scale using the table below. (See appendix 1)

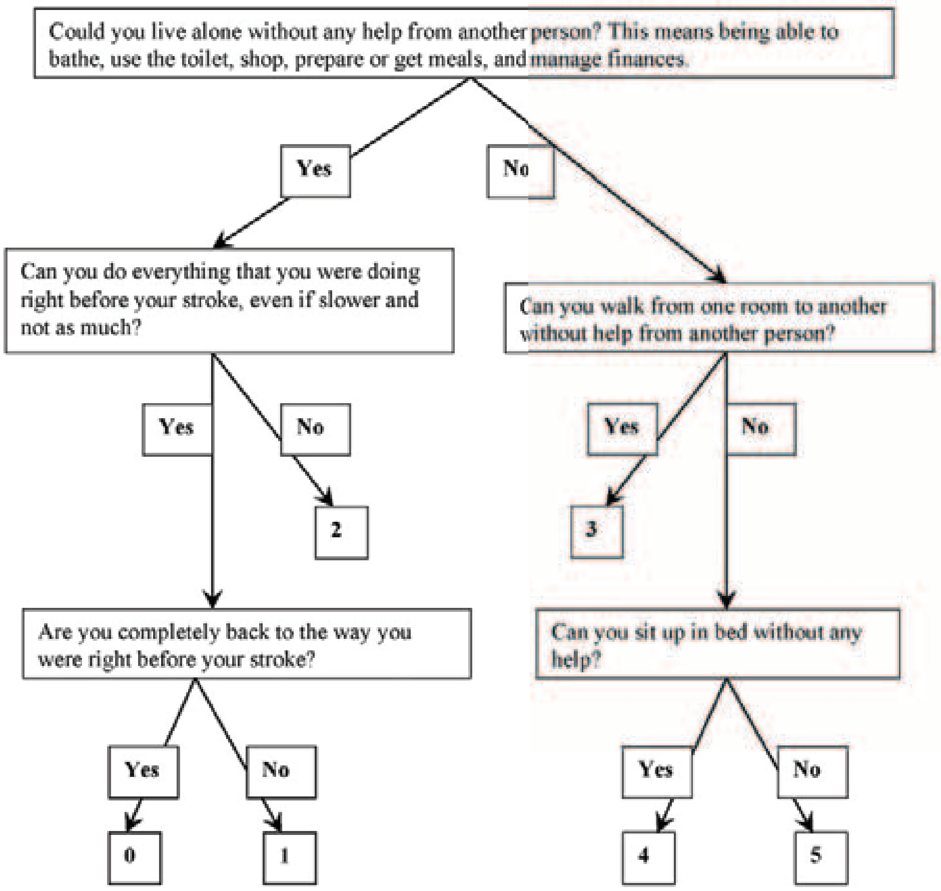

16.1.2 The National Institutes of Health Stroke Scale (NIHSS)

The NIHSS is a standardized neurological examination score that is a valid and reliable measure of disability and recovery after acute stroke[44]. Scores range from 0 to 42, with higher scores indicating more severe disability. The scale includes measures of level of consciousness, extra ocular movements, motor and sensory tests, coordination, language and speech evaluations. The NIHSS will be administered at baseline, Day 5-7 or discharge if earlier. The NIHSS will only be scored by those trained and certified in the use of this scale. In this trial, we will keep a video version

of NIHSS score except in those who die or are unable or refuse to take a video. The NIHSS score will be centrally assessed by two independent certified neurologists in a blinded manner via the video. Disagreements are resolved by consensus.(See appendix 2)

16.1.3 European Quality Five Dimensions Five Level scale

The EQ-5D-VAS is a generic instrument for describing and valuing health. The instrument is designed for self-completion, and respondents also rate their overall health on the day of the interview on a 0-100 hash-marked, vertical visual analogue scale. Patients who died will have a score of 0. The EQ-5D-VAS will be administered on Day 90±7 by those trained in the use of this scale. (See appendix 3)

16.2 Assessment of safety

16.2.1 Mortality at 90±7 days

Mortality at 90±7 days is one of the most important safety endpoints of the MARVEL trial. Mortality rates are defined as the number of deaths observed divided by the number of subjects observed over the 90-day study period.

16.2.2 Symptomatic Intracranial Haemorrhage

In addition to mortality, we will investigate SICH being the most feared complication for acute ischemic stroke. SICH within 48 hours will be adjudicated by an independent Imaging Core Laboratory and the Clinical Events Committee according to the Heidelberg Bleeding Classification.[45] SICH is diagnosed based on a

combination of: 1) clinical deterioration, 2) imaging findings, and 3) causal relatedness assessment. Clinical deterioration is defined as any of the following conditions: 1) NIHSS score increased more than 4 points than that immediately before worsening; 2) NIHSS score increased more than 2 points in one category; 3) Deterioration led to intubation, hemicraniectomy, external ventricular drain placement or any other major interventions. The imaging criteria are presence of parenchymal hematoma type 2 (PH2), parenchymal hematoma type 1 (PH1), remote intracerebral haemorrhage (RIH), subarachnoid haemorrhage (SAH), intraventricular haemorrhage (IVH), or subdural haemorrhage (SDH). When clinical deterioration and PH2 co-occur, the event is automatically categorized as SICH. When clinical deterioration and PH1/RIH/SAH/IVH/SDH co-occur, the event is deemed SICH if it is judged that the haemorrhage contributed substantially to the clinical worsening. When clinical deterioration occurs only with haemorrhagic infarction type 1 or 2 (HI1 or HI2), the event is automatically classified as not SICH.

16.2.3 Any ICH within 48 hours

The statistical analysis of differences in proportions of patients with any radiologic ICH within 48 hours between Methylprednisolone and Placebo groups will be performed.

The central reading will classify ICH according to the following intracerebral subtypes:

- Haemorrhagic infarct type 1 or type 2;

- Parenchymal hematoma type 1 or type 2;
- Remote intraparenchymal haemorrhage;
- In addition, intraventricular haemorrhage, subarachnoid haemorrhage, subdural haemorrhage, or epidural haemorrhage will be noted. ICH subtypes will be tabulated by treatment group.

16.2.4 Proportion of patients with pneumonia

Pneumonia is considered when at least 2 signs (body temperature > 38°C; leukocytosis >12 000/mL, or leukopenia <4000/mL; purulent pulmonary secretions) associated with the appearance of a new infiltrate are present or when changes occur in an existing infiltrate on chest x-ray. If necessary, the diagnosis will be confirmed by a respiratory tract sample using a quantitative culture with a predefined positive threshold of 10^4 colony-forming units per milliliter (CFU/mL) for a bronchoalveolar lavage or non bronchoscopic sample, of 10^3 CFU/mL for a protected specimen brush and of 10^6 CFU/mL for a tracheal sample. The incidence of pneumonia will be reported by local investigators.

16.2.4 Proportion of patients with gastrointestinal bleeding.

The statistical analysis of differences in proportions of patients with gastrointestinal bleeding within 7 days between Methylprednisolone and Placebo groups will be performed. Gastrointestinal bleeding will be reported by local investigators.

16.2.5 Adverse events

The safety assessments will include monitoring and recording adverse events, including serious and non-serious adverse events, measuring vital signs specified in the protocol, and other protocol specified tests that are considered to be critical to the safety evaluation of the study.

Any adverse change in health or the appearance of or worsening of any undesirable sign, symptom or medical condition occurring after enrolment into the trial will be recorded as Adverse Event whether or not it is considered to be related to the study drug. An adverse event also includes any occurrence that is a new illness; aggravated in severity of frequency from the baseline condition, abnormal results of diagnostic procedures, or a combination of the above. The assessment of severity and relationship of adverse events are determined by using the following definitions.

Table 3: Severity categorization and relationship definitions of Adverse Event.

Severity categorization		
Grade1	Mild	It is usually transient and may require only minimal treatment or therapeutic intervention. The event doesn’t generally interfere with usual activities of daily living.
Grade2	Moderate	It is usually alleviated with additional specific therapeutic intervention. The event interferes with usual activities of daily living, causing discomfort but poses no significant or permanent risk of harm to the research subject.
Grade3	Severe	It interrupts usual activities of daily living, or significantly affects clinical status, or may require intensive therapeutic intervention.

Grade4	Life-threatening	Substantial risk of dying at time of event
Grade5	Death	Death
Relationship of adverse events		
Related	A clinical event, including laboratory test abnormality, where there is a "reasonable possibility" that the serious adverse event was caused by the study medication, meaning that there is evidence or arguments to suggest a causal relationship.	
Probably	A clinical event, including laboratory test abnormality, with a reasonable time sequence to drug administration, unlikely to be attributed to concurrent disease or other drugs or chemicals, and which follows a clinically reasonable response on withdrawal.	
Possibly	A clinical event, including laboratory test abnormality, with a reasonable time sequence to drug administration, but which could also be explained by concurrent disease or other drugs or chemicals. Information on drug withdrawal may be lacking or unclear.	
Unrelated	This category is applicable to adverse events which are judged to be clearly and incontrovertibly due to extraneous causes (diseases, environment, etc.) and do not meet the criteria for drug relationship listed for the above-mentioned conditions.	

16.2.6 Serious Adverse Event

A Serious Adverse Event is any untoward medical occurrence (whether deemed to be related to investigational medication or not) that meets any of the following criteria:

- Fatal (i.e., the adverse event actually causes or leads to death)
- Life-threatening (i.e., the adverse event, in the view of the investigator, places the patient at immediate risk of death). This does not include any adverse event that, had it occurred in a more severe form or was allowed to continue, might have caused death.
- Requires or prolongs inpatient hospitalization
- Results in persistent or significant disability/incapacity (i.e., the adverse event results in substantial disruption of the patient's ability to conduct normal life functions)
- Congenital anomaly/birth defect in a neonate/infant born to a mother exposed to study drug
- Significant medical event in the investigator's judgment (e.g., may jeopardize the patient or may require medical/surgical intervention to prevent one of the outcomes listed above)

16.2.7 Adverse Event Recording and Follow Up

The investigator is responsible for evaluating and reporting any adverse events occurring during the study. The investigator will record onset, duration, intensity, any taken action, evolution/outcome, and the causality assessment for any adverse event.

Any adverse events leading to withdrawal from the study and those that persist at the end of the study must be followed up.

16.2.8 Serious Adverse Events Reporting

Although adverse events are recorded in the adverse event form of CRF special reporting requirements apply to the reporting of serious adverse events. Serious adverse events need to be reported immediately. The investigator should immediately notify the principal investigator of any serious adverse events that occurred at the trial site within 24 hours: MARVEL security desk Fax: +86 023-68774270

Serious adverse event reports will be collected centrally at the safety desk. Copies of all serious adverse event reports will be sent to the central trial management. Such preliminary reports will be followed by detailed descriptions, including anonymous copies of hospital case reports or related results, as well as other documents (if required and applicable). All serious adverse events that have not been resolved by the end of the study, or that have not been resolved upon discontinuation of the subject's participation in the study, must be followed until any of the following occurs:

- the event resolves
- the event stabilizes
- the event returns to baseline, if a baseline value is available
- the event can be attributed to agents other than the study drug or to factors unrelated to study conduct

- when it becomes unlikely that any additional information can be obtained

Any additional information known after the event has been initially reported should be sent using a new serious adverse event report form. New information will be noted on the "serious adverse event" form, by ticking the box marked "follow-up" and sending to MARVEL SAFETY DESK.

Patients who withdraw from the study treatment due to any adverse event will be followed up at least until the outcomes are determined, even if it implies that the follow-up continues after the patient has left the trial.

The investigator should assess the seriousness of the adverse events. This is based on the regulatory definitions of seriousness. The investigator should assess the causal relationship of serious adverse events. This is a clinical assessment of whether the adverse events may be related to the investigational medication. The evaluation of the expectedness is based on knowledge of adverse reactions and references.

17. Study Discontinuation Criteria

17.1 Patient Discontinuation

Patients have the right to voluntarily withdraw from the study at any time for any reason. In addition, the investigator has the right to withdraw the patient from the study at any time. Reasons for withdrawal from the study may include, but are not limited to the following:

- Patient withdraws consent at any time

- Any medical condition determined by the investigator or sponsor may endanger the patient's safety if he or she continues the study
- The investigator or sponsor determines that it is in the best interests of the patient
- Patient noncompliance, specifically defined as unwillingness to participate in a 3-month assessment of neurological status and function

Every effort should be made to obtain information about patients who withdrew from the study. The main reasons for withdrawal from the study should be recorded on the corresponding CRF. Patients who withdraw from the study will not be replaced.

17.2 Study Treatment Discontinuation

The treatment with the investigate medication should be terminated immediately if the patients experience any of the following:

- Any serious gastrointestinal bleeding.
- Uncontrollable hyperglycaemia (blood sugar >22.2 mmol/L)
- Anaphylactic reaction
- Continuation of the study drug would be detrimental to the patient’s well-being.
- Withdrawal for personal reasons.

According to the intention to treat (ITT) principle, patients who discontinue study treatment prematurely will continue in the study and be followed up to the 90th day.

17.3 Study and Site Discontinuation

The investigators has the right to terminate this study in any case of concern about the safety of patients caused by new information. The reasons for terminating the study may include, but are not limited to:

- The incidence or severity of adverse events in this or other studies indicates a potential health hazard to patients.
- Patient enrolment is unsatisfactory

The investigators have the right to close a site at any time. Reasons for closing a site may include, but are not limited to, the following:

- Excessively slow recruitment
- Poor protocol adherence
- Inaccurate or incomplete data recording
- Non-compliance with the International Conference on Harmonization guideline for Good Clinical Practice
- No study activity

18. Statistical Analysis

This study will test whether treatment of patients with acute ischemic stroke with intravenous methylprednisolone will lead to a lower mRS score at 90 days.

18.1 Determination of sample size

The current trial is designed detect a shift on the modified Rankin Scale score, which represents the global disability, to a lower score. The expected distribution of the modified Rankin Scale score, derived from the EVT for acute anterior circulation ischemic stroke (ACTUAL) registry[38] and the Highly Effective Reperfusion evaluated in Multiple Endovascular Stroke Trials (HERMES) collaboration[7], is as follows: 7%, 20%, 16%, 11%, 13%, 8% and 25%.

The sample size was calculated using the cumulative proportion of patients with mRS 0-2. We assume a moderate effect of 7% absolute increase in the cumulative proportion of patients with mRS 0-2 in the intervention group, compared with controls, indicating an odds ratio (OR) of 1.33, which have substantially exceeds the minimal clinically important difference.[39,40] In order to demonstrate the expected treatment effect with a type-1 error alpha =0.05 (two-tailed) and a power of 80% (beta = 20%). A sample size of n= 1588 patients (n=794 per treatment group) is required.

The intention-to-treat principle will be applied to the primary analysis and, therefore, to safeguard against dilution of the treatment effect associated with an approximate 5% non-adherence rate (due to loss to follow-up, consent withdrawal and other reasons), we initially planned to enrol n=1672 patients (n=836 per treatment group) for this study. This estimation was performed based on PASS (NCSS, LLC. Kaysville, Utah, USA) version 15.0.

18.2 Analysis population

18.2.1 Intention-to-Treat Population

The ITT population includes all patients randomized into the trial who were recorded as receiving any amount of study drug, even if the subject does not receive the correct treatment, or does not follow the protocol until completion[46]. The ITT population will be the primary analysis population for the efficacy endpoints and subjects will be analysed according to the treatment group to which they were assigned at randomization.

18.2.2. Per-Protocol Population

The PP population is defined as the subset of the ITT population excluding major protocol violators deemed to have the potential to affect patient outcome in terms of efficacy.

The PP population includes patients who actually received the assigned treatment and do not have major protocol violations or deviations. Major protocol violations or deviations will be identified in a blinded fashion prior to database lock. More specifically, patients with anyone of the following criteria will be excluded from the PP population. These deviations will be determined based on the medical monitors' records, as well as programmatically, using the following criteria at a minimum:

- Received but did not complete treatment with study drug, or dose of study drug administered outside recommended dose.
- Received study drug after 2 hours after arterial access closure.
- Violated inclusion or exclusion criteria.

A list of patients to be excluded from the randomized patients to create the PP-Efficacy analysis will be established and validated by the Steering Committee prior to unblinding.

18.2.3. Safety Population

The Safety Population includes all patients who received any amount of study drug. In case of violation of the randomization scheme, patients will be classified according to the treatment they actually received. Patients will be assigned to the different populations prior to unblinding of the database. Patients who withdraw informed consent immediately after randomization and are not to receive any treatment should be excluded from Safety Population.

18.2 Analysis of Efficacy–primary endpoint

The primary efficacy analysis will estimate the effect of treatment with intravenous methylprednisolone and with the placebo in acute ischemic stroke patients.

The primary efficacy outcome is the global disability defined by mRS score at 90 days post-randomization. Adjusted common odds ratio or assumption-free method will be used. The adjusted covariates include age, baseline NIHSS score, pre-stroke mRS, baseline ASPECTS score, use of intravenous thrombolysis, time from onset to randomization, and occlusion location. (Details are described in SAP Section 12.1).

We will analyse the primary outcome in the intention-to-treat (ITT) and per-protocol

(PP) population.

18.3 Analysis of Efficacy – secondary endpoints

The mRS score will be used to evaluate the proportion of patients with a score = 0 to 4 *versus* 5 or 6, 0 to 3 *versus* 4 or higher, 0 to 2 *versus* 3 or higher, 0 to 1(or return to pre stroke morbidity) *versus* 2 or higher and at day 90±7. The adjusted risk ratio will be calculated by fitting the GLM models separately.

NIHSS score at 5-7 days or at early discharge and Functional health status quality of life 90 (±7) days after randomization (EQ-5D-VAS). The EQ-5D-VAS will be compared between the methylprednisolone group and the placebo group via GLM.

Normality and variance homogeneity of residuals will be accessed graphically. The win ratio method will be used if normality assumption is seriously violated.

The significance of each test is determined at the two-sided alpha level of 0.05. The specific statistical model for analysing each of these outcome measures will be detailed in the Statistical Analysis Plan (SAP).

18.4 Safety analysis

Safety outcomes include incidence of overall mortality at 90 (±7) days after randomization, the proportion of patient with SICH within 48 hours after treatment, the proportion of patient with any ICH within 48 hours after treatment, proportion of patients with pneumonia and proportion of patients with gastrointestinal haemorrhage within 7 days after EVT. The safety analysis will be performed on the Safety

Population. The incidences of each outcome will be compared via Chi-square test or Fisher's exact test. The modified Poisson regression models will be fitted to estimate the risk-ratio associated with the treatment effect.[47] Risk ratio with 95% CI will be reported. Patients with missing outcome will not be included in the regression analysis. Additionally, Log-Rank test and the Kaplan-Meier estimates will be plotted over the observation period of 90 days for mortality. Cox regression model will be employed to calculate the hazard ratio with 95%CI.

18.5 Tolerability Analysis (adverse events)

Tolerability analyses will be performed on the Safety Population only.

18.5.1 Adverse Events

Adverse Events will be coded using the Medical Dictionary for Regulatory Activities (MedDRA) central coding dictionary.

18.5.2 Serious Adverse Events

Serious Adverse Event reconciliation will be performed by Data Management, Clinical Research, and Pharmacovigilance via data listings.

A summary of patients with serious adverse events will be presented by treatment group for all patients in the Safety Population. A data listing of SAEs will also be provided, displaying details of the event(s) captured on the CRF. The between group difference will be tested using Chi-square test or Fisher's exact test.

18.5.3 All Adverse Events

Summaries of patients with AEs by System Organ Class and Preferred Term (MedDRA) will be prepared. Each patient will be counted only once within each category (System Organ Class or Preferred Term). If a patient experiences more than one AE within a category, only the AE with the strongest relationship or the greatest intensity, as appropriate, will be included in the summaries. The between group difference will be tested using Chi-square test or Fisher's exact test. Only AEs beginning at or after the beginning of study drug administration will be included.

18.6 Adjustment for Covariates and Subgroup Analyses

In addition to the primary and secondary analyses adjusting for age, baseline NIHSS score, pre-stroke mRS, baseline ASPECTS score, the use of intravenous thrombolysis, time from onset to randomization, and occlusion location, exploratory analyses will be conducted to determine the potential roles of common baseline characteristics and assess potential heterogeneity of treatment effect across subgroups. The primary pre-specified subgroups as follows will be explored.

- age
- sex
- baseline NIHSS score
- pre-stroke mRS
- baseline ASPECTS score
- intravenous thrombolysis

- time from last known well to randomization. (mins)
- occlusion location
- Complete Reperfusion defined as Extended Thrombolysis in Cerebral Infarction grade 2c or 3
- Patients with any radiological haemorrhages
- Patients with symptomatic haemorrhages

18.7 Missing data handling

All efforts will be made to minimize the amount of missing data. However, some missing data may be inevitable due to, for example, loss to follow-up. Missing baseline covariates will be imputed using simple imputation or multiple imputation methods (Details in SAP Section 9.2.2). Sensitivity analyses based on different hypotheses about the missingness pattern of the primary outcome will be performed to test for the robustness of the primary analysis (Details in SAP Section 12.1.5).

19. Ethical and regulatory consideration

19.1 General requirements and Considerations

This study followed the ethical principles of the Helsinki Declaration. Approval of the conduct of the trial will be obtained from the Ethics Committees of all participating centres as well as from the local regulatory authorities. The trial will not start in any centre before written approval and authorization by the respective Ethics Committee and Regulatory Authority. Any subsequent protocol amendment will be submitted to

the Ethics Committee for approval. The involvement of committees in the clinical trial of MARVEL will further ensure that the subjects have the highest priority at any time. In addition to the treatment to be tested, all diagnostic procedures and treatments applied are part of standard management of acute stroke patients and will follow the national guidelines. These procedures are therefore of immediate benefit to the patients. The investigators will assure that every patient participating in the trial will receive best medical treatment.

19.2 Study monitoring and quality control

The design of the MARVEL trial has been carefully reviewed and approved by the Steering Committee before being submitted to the Ethics Committees and Regulatory Authorities for approval. In addition, the independent EAB and DSMB and an external SAB have reviewed and approved the trial protocol and will continuously monitor the conduction of the trial. These committees, composed of well-known independent experts, will ensure sufficient alertness to all ethical and safety issues.

The investigators promise to conduct the MARVEL trial in accordance with this trial protocol, International Conference on Harmonization-GCP (ICH-GCP) Guidelines and applicable regulatory requirements. The investigators agree to provide reliable data and all information required by the trial protocol in an accurate and legible manner according to the instructions provided.

The investigators of this clinical trial is responsible to the health authorities and takes all reasonable measures to ensure the correct implementation of the clinical trial

protocol in terms of ethics, clinical trial protocol compliance, and the completeness and validity of the data recorded on the CRF.

The main responsibility of the monitoring team is to help investigators to ensure that all aspects of clinical trials are highly ethical, scientific, professional and standardized. The monitoring team will regularly contact the centres through field visits, emails or phone calls, and send inspectors to assess the progress of the trial, the compliance of investigators and patients with the trial protocol, and to resolve urgent issues. During these inspection visits, the inspector will work with the on-site investigator. The main aspects of inspection and monitoring are as follows (not exclusive): patient informed consent, patient recruitment and follow-up, serious adverse event recording and reporting, study drug supply, treatment compliance of study drug group participants, study drug count, concomitant treatment and data quality.

19.3 Informed Consent

The written informed consent must be obtained from all participants in the clinical trial prior to inclusion into the study. Informed consent forms must be written to be easily understood by the participants or their legal representatives, enabling them to understand the purpose of the trial, procedures, possible benefits, potential risks, and the rights/obligations of participation. Participants have the right to withdraw from the study at any stage of the trial. Each participant must leave contact information to the investigator of the coordinating centre. At the same time, the investigator must leave

his own phone number to the participant so that the participant can find the investigator at any time.

19.4 Confidentiality

Personal data will be processed in accordance with Chinese data protection directives and regulations, relevant international legislation and good practices. Data will only be processed for the trial's purposes. The investigators encode each patient participating in the study by assigning a unique patient identification number to maintain confidentiality standards. This means that all individual patients' data will be linked to the CRF via a unique identification number throughout the trial. Individual patient's medical information will be recorded only in anonymous form. The clinical monitors may inspect source data in order to ensure the accuracy of the data recorded in the CRF.

19.5 Liability and Insurance

The study investigators provide an appropriate insurance for patients in the event of any trial related damage in accordance with applicable national laws. A certificate of insurance will be provided to the investigator of the coordinating centre in which this document is required.

20. Administrative procedures

20.1 Curriculum vitae

A latest copy of the curriculum vitae of each investigator and co-investigator will be provided to the responsible coordinating centre prior to the start of the study.

20.2 Secrecy agreement

The investigators will take all necessary measures to ensure that there is no violation of confidentiality in respect of all information accumulated, acquired or deduced in the course of the trial, other than that information to be disclosed by law.

20.3 Ownership of data and use of the study results

All materials, information and unpublished documentation supplied to the investigators, inclusive of this study, and the patient case report forms are the exclusive property of the study initiators. Therefore, the study initiators reserve the right to use the data of the present study, either in the form of case report forms, or in the form of a report, with or without comments and with or without analysis, in order to submit them to the health authorities.

20.4 Protocol amendments

Any protocol amendments will be prepared by the investigators. Protocol amendments should be submitted to the REB/IRB for approval prior to implementation in accordance with local regulatory requirements. Approval must be obtained from the REB/IRB and regulatory authorities (as locally required) before implementation of any changes, except for changes necessary to eliminate an immediate hazard to patients or changes that involve logistical or administrative aspects only

21. Data Retention

The double reviewed case report form (CRF) and imaging data will be sent to the data management group. The person in charge of the data management group will check and sign the receipt form. The CRF will be kept by the research centre after data entry is completed.

22. Study report and publications

The results of the trial will be reported to the regulatory authorities and ethics committees. The investigators will provide an annual safety report and the final report.

According to the pre-defined analysis in the clinical trial protocol, the results of the trial will be published in the appropriate journal (for manuscripts) or meeting (for abstracts).

By signing the clinical trial protocol， the investigator agrees that the results of the clinical trial can be used for publication.

The trial will be registered at the Chinese Clinical Trial Registry website.

23. ReferencesUncategorized References

1. Global, regional, and national burden of stroke and its risk factors, 1990-2019: a systematic analysis for the Global Burden of Disease Study 2019. Lancet Neurol 2021;20:795-820.

2. Diseases GBD, Injuries C. Global burden of 369 diseases and injuries in 204 countries and territories, 1990-2019: a systematic analysis for the Global Burden of Disease Study 2019. Lancet 2020;396:1204-22.

3. Lindsay MP, Norrving B, Sacco RL, et al. World Stroke Organization (WSO): Global Stroke Fact Sheet 2019. Int J Stroke 2019;14:806-17.

4. Wang W, Jiang B, Sun H, et al. Prevalence, Incidence, and Mortality of Stroke in China: Results from a Nationwide Population-Based Survey of 480 687 Adults. Circulation 2017;135:759-71.

5. Baron JC. Protecting the ischaemic penumbra as an adjunct to thrombectomy for acute stroke. Nat Rev Neurol 2018;14:325-37.

6. Powers WJ, Rabinstein AA, Ackerson T, et al. Guidelines for the Early Management of Patients With Acute Ischemic Stroke: 2019 Update to the 2018 Guidelines for the Early Management of Acute Ischemic Stroke: A Guideline for Healthcare Professionals From the American Heart Association/American Stroke Association. Stroke 2019;50:e344-e418.

7. Goyal M, Menon BK, van Zwam WH, et al. Endovascular thrombectomy after large-vessel ischaemic stroke: a meta-analysis of individual patient data from five randomised trials. LANCET 2016;387:1723-31.

8. Nie X, Pu Y, Zhang Z, Liu X, Duan W, Liu L. Futile Recanalization after Endovascular Therapy in Acute Ischemic Stroke. Biomed Res Int 2018;2018:5879548.

9. Stoll G, Pham M. Beyond recanalization — a call for action in acute stroke. Nature Reviews Neurology 2020;16:591-2.

10. Whiteley WN, Emberson J, Lees KR, et al. Risk of intracerebral haemorrhage with alteplase after acute ischaemic stroke: a secondary analysis of an individual patient data meta-analysis. Lancet Neurol 2016;15:925-33.

11. Shi ZS, Duckwiler GR, Jahan R, et al. Early Blood-Brain Barrier Disruption after Mechanical Thrombectomy in Acute Ischemic Stroke. J Neuroimaging 2018;28:283-8.

12. Conti E, Piccardi B, Sodero A, et al. Translational Stroke Research Review: Using the Mouse to Model Human Futile Recanalization and Reperfusion Injury in Ischemic Brain Tissue. Cells 2021;10:19.

13. Cheripelli BK, Huang XY, MacIsaac R, Muir KW. Interaction of Recanalization, Intracerebral Hemorrhage, and Cerebral Edema After Intravenous Thrombolysis. Stroke 2016;47:1761-7.

14. Chen XL, Huang Q, Deng QW, et al. A prediction model of brain edema after endovascular treatment in patients with acute ischemic stroke. Journal of the Neurological Sciences 2019;407.

15. Ames A, 3rd, Wright RL, Kowada M, Thurston JM, Majno G. Cerebral ischemia. II. The no-reflow phenomenon. The American journal of pathology 1968;52:437-53.

16. Caiazzo G, Musci RL, Frediani L, et al. State of the Art: No-Reflow Phenomenon. Cardiol Clin 2020;38:563-73.

17. Kloner RA, King KS, Harrington MG. No-reflow phenomenon in the heart and brain. Am J Physiol Heart Circ Physiol 2018;315:H550-H62.

18. Planas AM. Role of Immune Cells Migrating to the Ischemic Brain. Stroke 2018;49:2261-7.

19. Shi KB, Tian DC, Li ZLG, Ducruet AF, Lawton MT, Shi FD. Global brain inflammation in stroke. Lancet Neurology 2019;18:1058-66.

20. Laha RK, Dujovny M, Barrionuevo PJ, DeCastro SC, Hellstrom HR, Maroon JC. Protective effects of

methyl prednisolone and dimethyl sulfoxide in experimental middle cerebral artery embolectomy. J Neurosurg 1978;49:508-16.
21. Slivka AP, Murphy EJ. High-dose methylprednisolone treatment in experimental focal cerebral ischemia. Exp Neurol 2001;167:166-72.
22. de Courten-Myers GM, Kleinholz M, Wagner KR, Xi G, Myers RE. Efficacious experimental stroke treatment with high-dose methylprednisolone. Stroke 1994;25:487-92; discussion 93.
23. Barbosa-Coutinho LM, Hartmann A, Hossmann KA, Rommel T. Effect of dexamethasone on serum protein extravasation in experimental brain infarcts of monkey: an immunohistochemical study. Acta Neuropathol 1985;65:255-60.
24. Espinosa A, Meneses G, Chavarría A, et al. Intranasal Dexamethasone Reduces Mortality and Brain Damage in a Mouse Experimental Ischemic Stroke Model. Neurotherapeutics 2020;17:1907-18.
25. Altamentova S, Rumajogee P, Hong J, et al. Methylprednisolone Reduces Persistent Post-ischemic Inflammation in a Rat Hypoxia-Ischemia Model of Perinatal Stroke. Transl Stroke Res 2020;11:1117-36.
26. Limbourg FP, Huang Z, Plumier JC, et al. Rapid nontranscriptional activation of endothelial nitric oxide synthase mediates increased cerebral blood flow and stroke protection by corticosteroids. J Clin Invest 2002;110:1729-38.
27. Norris JW. Steroids may have a role in stroke therapy. Stroke 2004;35:228-9.
28. Nellis SH, Roberts BH, Kinney EL, Field J, Ummat A, Zelis R. Beneficial effect of dexamethasone on the "no reflow" phenomenon in canine myocardium. Cardiovasc Res 1980;14:137-41.
29. Sandercock PAG, Soane T. Corticosteroids for acute ischaemic stroke. Cochrane Database of Systematic Reviews 2011.
30. Ogun SA, Odusote KA. Effectiveness of high dose dexamethasone in the treatment of acute stroke. West African journal of medicine 2001;20:1-6.
31. Lyden P, Buchan A, Boltze J, et al. Top Priorities for Cerebroprotective Studies—A Paradigm Shift: Report From STAIR XI. Stroke 2021;52:3063-71.
32. Lapchak PA. Emerging Therapies: Pleiotropic Multi-target Drugs to Treat Stroke Victims. Translational Stroke Research 2011;2:129-35.
33. Fraser JF, Pahwa S, Maniskas M, et al. Now that the door is open: an update on ischemic stroke pharmacotherapeutics for the neurointerventionalist. Journal of Neurointerventional Surgery 2023.
34. Savitz SI, Baron JC, Fisher M, Consortium SX. Stroke Treatment Academic Industry Roundtable X: Brain Cytoprotection Therapies in the Reperfusion Era. Stroke 2019;50:1026-31.
35. Savitz SI, Baron J-C, Yenari MA, Sanossian N, Fisher M. Reconsidering Neuroprotection in the Reperfusion Era. 2017;48:3413-9.
36. Davis SM, Donnan GA. Steroids for stroke: another potential therapy discarded prematurely? Stroke 2004;35:230-1.
37. Huting ZHANG SL. Influence of neurological intervention combined with dexamethasone injection on the outcome for the patients suffered from acute ischemic stroke. Journal of Molecular Imaging 2016;39:121-4.
38. Zi W, Wang H, Yang D, et al. Clinical Effectiveness and Safety Outcomes of Endovascular Treatment for Acute Anterior Circulation Ischemic Stroke in China. Cerebrovasc Dis 2017;44:248-58.
39. Cranston JS, Kaplan BD, Saver JL. Minimal Clinically Important Difference for Safe and Simple Novel Acute Ischemic Stroke Therapies. Stroke 2017;48:2946-51.
40. Berkhemer OA, Fransen PSS, Beumer D, et al. A Randomized Trial of Intraarterial Treatment for Acute Ischemic Stroke. New England Journal of Medicine 2015;372:11-20.

41. Wang Y, Han S, Qin H, et al. Chinese Stroke Association guidelines for clinical management of cerebrovascular disorders: executive summary and 2019 update of the management of high-risk population. Stroke Vasc Neurol 2020;5:270-8.
42. Banks JL, Marotta CA. Outcomes validity and reliability of the modified Rankin scale: implications for stroke clinical trials: a literature review and synthesis. Stroke 2007;38:1091-6.
43. Quinn TJ, Dawson J, Walters MR, Lees KR. Reliability of the modified Rankin Scale: a systematic review. Stroke 2009;40:3393-5.
44. Brott T, Adams HP, Jr., Olinger CP, et al. Measurements of acute cerebral infarction: a clinical examination scale. Stroke 1989;20:864-70.
45. von Kummer R, Broderick JP, Campbell BC, et al. The Heidelberg Bleeding Classification: Classification of Bleeding Events After Ischemic Stroke and Reperfusion Therapy. Stroke 2015;46:2981-6.
46. White IR, Horton NJ, Carpenter J, Pocock SJ. Strategy for intention to treat analysis in randomised trials with missing outcome data. Bmj 2011;342:d40.
47. Zou G. A modified poisson regression approach to prospective studies with binary data. Am J Epidemiol 2004;159:702-6.

25. Appendices

25.1 Appendix 1: mRS

Subject ID ___ ___ ___ ___ ___ ___ ___ ___ ___ ___
Subject Date of Birth ___ ___/___ ___/___ ___
Hospital ID ______________________________
Date of Examination ___ ___/___ ___/___ ___

The Modified Rankin Scale (mRS)

(Use web calculator at www.modifiedrankin.com)

0 No symptoms

1 No significant disability; able to carry out all usual activities, despite some symptoms

2 Slight disability; able to look after own affairs without assistance, but unable to carry out all previous activities

3 Moderate disability; requires some help, but able to walk unassisted

4 Moderately severe disability; unable to attend to own bodily needs without assistance, and unable to walk unassisted

5 Severe disability; requires constant nursing care and attention, bedridden, incontinent

6 Dead

References:

Rankin J (May 1957). "Cerebral vascular accidents in patients over the age of 60. II. Prognosis". Scott Med J 2 (5): 200–15

Patel, N., et al. Simple and reliable determination of the modified Rankin Scale in neurosurgical and neurological patients: The mRS-9Q. *Neurosurgery*, published online in advance of print 26 July 2012

25.2 Appendix 2: NIHSS

NIHSS page 1

Subject ID ___ ___ ___ ___ ___ ___ ___ ___ ___ ___
Subject Date of Birth ___ ___/___ ___/___ ___
Hospital ID ____________________
Date of Examination ___ ___/___ ___/___ ___

NATIONAL INSTITUTES OF HEALTH STROKE SCALE (NIHSS)[1]

Interval: [ ] Baseline
[ ] 7-10 days
[ ] 1 month
[ ] 3 months
[ ] 6 months
[ ] Other ____________________

Time: ___ ___:___ ___ [ ]am [ ]pm

Person Administering Scale ____________________

Purpose:
The NIH Stroke Scale (NIHSS) is a standardized neurological examination intended to describe the neurological deficits found in large groups of stroke patients participating in treatment trials.

Administer stroke scale items in the order listed. Record performance in each category after each subscale exam. Do ***not*** go back and change scores. Follow directions provided for each exam technique. Scores should reflect what the patient does, not what the clinician thinks the patient can do. The clinician should record answers while administering the exam and work quickly. Except where indicated, the patient should not be coached (i.e., repeated requests to patient to make a special effort).

Instructions	Scale Definition	Score
**1a. Level of Consciousness:** The investigator must choose a response if a full evaluation is prevented by such obstacles as an endotracheal tube language barrier, orotracheal trauma/bandages. A 3 is scored only if the patient makes no movement (other than reflexive posturing) in response to noxious stimulation.	0 = **Alert**; Keenly responsive.<br>1 = **Not alert, but arousable** by minor stimulation to obey, answer or respond.<br>2 = **Not alert**; requires repeated stimulation to attend, or is obtunded and requires strong or painful stimulation to make movements (not stereotyped).<br>3 = **Responds only with reflex motor or autonomic effects or totally unresponsive**, flaccid, and areflexic.	____
**1b. LOC Questions:** The patient is asked the month and his/her age. The answer must be correct – there is no partial credit for being close. Aphasic and stuporous patients who do not comprehend the questions will score 2. Patients unable to speak because of endotracheal intubation, orotracheal trauma, severe dysarthria from any cause, language barrier, or any other problem not secondary to aphasia are given a 1. It is important that only the initial answer be graded and that the examiner not "help" the patient with verbal or non-verbal cues.	0 = **Answers both questions correctly.**<br>1 = **Answers one question correctly.**<br>2 = **Answers neither question correctly.**	____

[1] The National Institute of Neurological Diseases and Stroke (NINDS), National Institutes of Health (NIH), Last Revised 01 October 2003 (https://stroke.nih.gov/resources/index.htm).

NIHSS page 2

Instructions	Scale Definition	Score
**6. Motor Leg:** The limb is placed in the appropriate position: hold the leg at 30 degrees (always tested supine). Drift is scored if the leg falls before 5 seconds. The aphasic patient is encouraged using urgency in the voice and pantomime, but not noxious stimulation. Each limb is tested in turn, beginning with the non-paretic leg. Only the case of amputation or joint fusion at the hip, should the examiner record the score as untestable (UN), and clearly write the explanation for this choice.	0 = **No drift**; limb holds 90 (or 45) degrees for full 10 seconds.<br>1 = **Drift**; limb holds 90 (or 45) degrees, but drifts down before full 10 seconds; does not hit bed or other support.<br>2 = **Some effort against gravity**; limb cannot get to or maintain (if cued) 90 (or 45) degrees, drifts down to bed, but has some effort against gravity.<br>3 = **No effort against gravity**; leg falls to bed immediately.<br>4 = **No movement**.<br>UN= **Amputation** or joint fusion; explain: ____________<br>6a= **Left Arm**.<br>6b= **Right Arm**.	________<br>________
**7. Limb Ataxia:** This item is aimed at finding evidence of a unilateral cerebellar lesion. Test with eyes open. In case of visual defect, ensure testing is done in intact visual field. The finger-nose-finger and heel-shin tests are performed on both sides, and ataxia is scored only if present out of proportion to weakness. Ataxia is absent in the patient who cannot understand or is paralyzed. Only in the case of amputation or joint fusion, should the examiner record the score as untestable (UN), and clearly write the explanation for this choice. In case of blindness, test by having the patient touch nose from extended arm position.	0 = **Absent.**<br>1 = **Present in one limb**.<br>2 = **Present in two limbs**.<br>UN= **Amputation** or joint fusion; explain: ____________	________
**8. Sensory:** Sensation or grimace to pinprick when tested, or withdrawal from noxious stimulus in the obtunded or aphasic patient. Only sensory loss attributed to stroke is scored as abnormal and the examiner should test as many body areas [arms (not hands), legs, trunk, face) as needed to accurately check for hemisensory loss. A score of 2, "severe or total sensory loss," should only be given when a severe or total loss of sensation can be clearly demonstrated. Stuporous and aphasic patients will, therefore, probably score 1 or 0. The patient with brainstem stroke who has bilateral loss of sensation is scored 2. If the patient does not respond and is quadriplegic, score 2. Patients in a coma (item 1a=3) are automatically given a 2 on this item.	0 = **Normal**; no sensory loss.<br>1 = **Mild-to-moderate** sensory loss; patient feels pinprick is less sharp or is dull on the affected side, or there is a loss of superficial pain with pinprick, but patient is aware of being touched.<br>2 = **Severe to total** sensory loss; patient is not aware of being touched in the face, arm, and leg.	________
**9. Best Language:** A great deal of information about comprehension will be obtained during the preceding sections of the examination. For this scale item, the patient is asked to describe what is happening in the attached picture, to name the items on the attached naming sheet and to read from the attached list of sentences. Comprehension is judged from responses here, as well as to all of the commands in the preceding general neurological exam. If visual loss interferes with the tests, ask the patient to identify objects placed in the hand, repeat, and produce speech. The intubated patient should be asked to write. The patient in a coma (item 1a=3) will automatically score 3 on this item. The examiner must choose a score for the patient with stupor or limited cooperation, but a score of 3 should be used only if the patient is mute and follows no one-step commands.	0 = **No asphasia**; normal.<br>1 = **Mild-to-moderate aphasia**; some obvious loss of fluency or facility of comprehension, without significant limitation on ideas expressed or form of expression. Reduction of speech and/or comprehension, however, makes conversation about provided materials difficult or impossible. For example, in conversation about provided materials, examiner can identify picture or naming card content from patient's response.<br>2 = **Severe asphasia**; all communication is through fragmentary expression; great need for inference, questioning, and guessing by the listener. Range of information that can be exchanged is limited; listener carries burden of communication. Examiner cannot identify materials provided from patient response.<br>3 = **Mute, global aphasia**; no usable speech or auditory comprehension.	________

NIHSS page 3

Instructions	Scale Definition	Score
**1c. LOC Commands:** The patient is asked to open and close the eyes and then to grip and release the non-paretic hand. Substitute another one step command if the hands cannot be used. Credit is given if an unequivocal attempt is made but not completed due to weakness. If the patient does not respond to command, the task should be demonstrated to him/her (pantomime), and the result scored (i.e., follows none, one or two commands). Patients with trauma, amputation, or other physical impediments should be given suitable one-step commands. Only the first attempt is scored.	0 = **Answers both tasks correctly**.<br>1 = **Answers one task correctly**.<br>2 = **Answers neither task correctly**.	________
**2. Best Gaze**: Only horizontal eye movements will be tested. Voluntary or reflexive (oculocephalic) eye movements will be scored, but caloric testing is not done. If the patient has a conjugate deviation of the eyes that can be overcome by voluntary or reflexive activity, the score will be 1. If a patient has an isolated peripheral nerve paresis (CN III, IV or VI), score a 1. Gaze is testable in all aphasic patients. Patients with ocular trauma, bandages, pre-existing blindness, or other disorder of visual acuity or fields should be tested with reflexive movements, and a choice made by the investigator. Establishing eye contact and then moving about the patient from side to side will occasionally clarify the presence of a partial gaze palsy.	0 = **Normal.**<br>1 = **Partial gaze palsy**; gaze is abnormal in one or both eyes, but forced deviation or total gaze paresis is not present.<br>2 = **Forced deviation**, or total gaze paresis not overcome by the oculocephalic maneuver.	________
**3. Visual:** Visual fields (upper and lower quadrants) are tested by confrontation, using finger counting or visual threat, as appropriate. Patients may be encouraged, but if they look at the side of the moving fingers appropriately, this can be scored as normal. If there is unilateral blindness or enucleation, visual fields in the remaining eye are scored. Score 1 only if a clear-cut asymmetry, including quadrantanopia, is found. If patient is blind from any cause, score 3. Double simultaneous stimulation is performed at this point. If there is extinction, patient receives a 1, and the results are used to respond to item 11.	0 = **No visual loss**.<br>1 = **Partial hemianopia**.<br>2 = **Complete hemianopia**.<br>3 = **Bilateral hemianopia** (blind including cortical blindness).	________
**4. Facial Palsy:** Ask – or use pantomime to encourage – the patient to show teeth or raise eyebrows and close eyes. Score symmetry of grimace in response to noxious stimuli in the poorly responsive or non-comprehending patient. If facial trauma/bandages, orotracheal tube, tape or other physical barriers obscure the face, these should be removed to the extent possible.	0 = **Normal** symmetrical movements.<br>1 = **Minor paralysis** (flattened nasolabial fold, asymmetry on smiling).<br>2 = **Partial paralysis** (total or near-total paralysis of lower face).<br>3 = **Complete paralysis** of one or both sides (absence of facial movement in the upper and lower face	________
**5. Motor Arm:** The limb is placed in the appropriate position: extend the arms (palms down) 90 degrees (if sitting) or 45 degrees (if supine). Drift is scored if the arm falls before 10 sounds. The aphasic patient is encouraged using urgency in the voice and pantomime, but not noxious stimulation. Each limb is tested in turn, beginning with the non-paretic arm. Only in the case of amputation or joint fusion at the shoulder, should the examiner record the score as untestable (UN), and clearly write the explanation for this choice.	0 = **No drift**; limb holds 90 (or 45) degrees for full 10 seconds.<br>1 = **Drift**; limb holds 90 (or 45) degrees, but drifts down before full 10 seconds; does not hit bed or other support.<br>2 = **Some effort against gravity**; limb cannot get to or maintain (if cued) 90 (or 45) degrees, drifts down to bed, but has some effort against gravity.<br>3 = **No effort against gravity**; limb falls.<br>4 = **No movement**.<br>UN= **Amputation** or joint fusion; explain: ____________________<br>5a= **Left Arm**.<br>5b= **Right Arm**.	________<br>________

NIHSS page 4

Instructions	Scale Definition	Score
**10. Dysarthria:** If patient is thought to be normal, an adequate sample of speech must be obtained by asking patient to read or repeat words from the attached list. If the patient has severe aphasia, the clarity of articulation of spontaneous speech can be rated. Only if the patient is intubated or has other physical barriers to producing speech, should the examiner record the score as untestable (UN), and clearly write an explanation for this choice. Do not tell the patient why he or she is being tested.	0 = **Normal**.<br>1 = **Mild-to-moderate dysarthria**; patient slurs at least some words and, at worst, can be understood with some difficulty.<br>2 = **Severe dysarthria**; patient's speech is so slurred as to be unintelligible in the absence of or out of proportion to any dysphasia, or is mute/anarthric.<br>UN= **Intubated** or other physical barrier; explain: ______________________	________
**11. Extinction and Inattention (formerly Neglect):** Sufficient information to identify neglect may be obtained during the prior testing. If the patient has a severe visual loss preventing visual double simultaneous stimulation, and the cutaneous stimuli are normal, the score is normal. If the patient has aphasia but does appear to attend to both sides, the score is normal. The presence of visual spatial neglect or anosagnosia may also be taken as evidence of abnormality. Since the abnormality is scored only if present, the item is never untestable.	0 = **No abnormality**.<br>1 = **Visual, tactile, auditory, spatial, or personal inattention** or extinction to bilateral simultaneous stimulation in one of the sensory modalities.<br>2 = **Profound hemi-inattention or extinction to more than one modality**; does not recognize own hand or orients to only one side of space.	________

NIHSS page 5

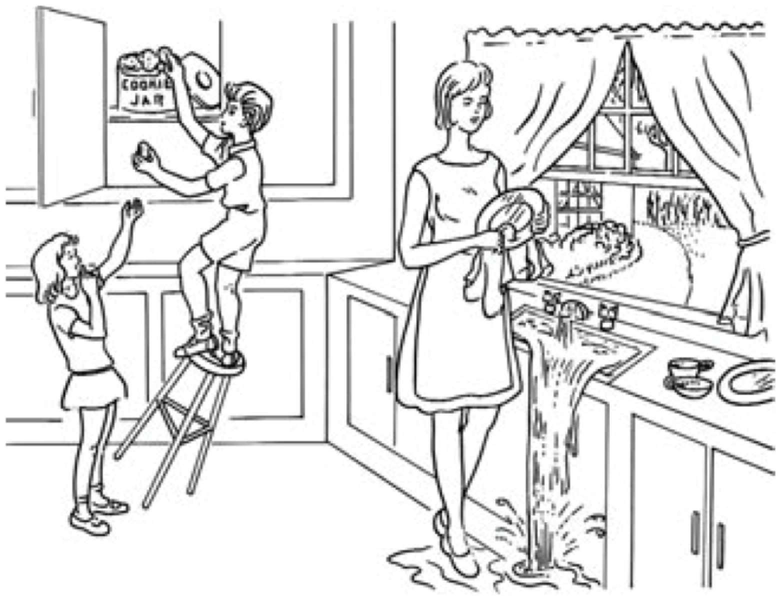

You know how.

Down to earth.

I got home from work.

Near the table in the dining room.

They heard him speak on the radio last night.

请您读出下列句子：

知道

下楼梯

回家做饭

在学校复习

发表精彩演讲

NIHSS page 6

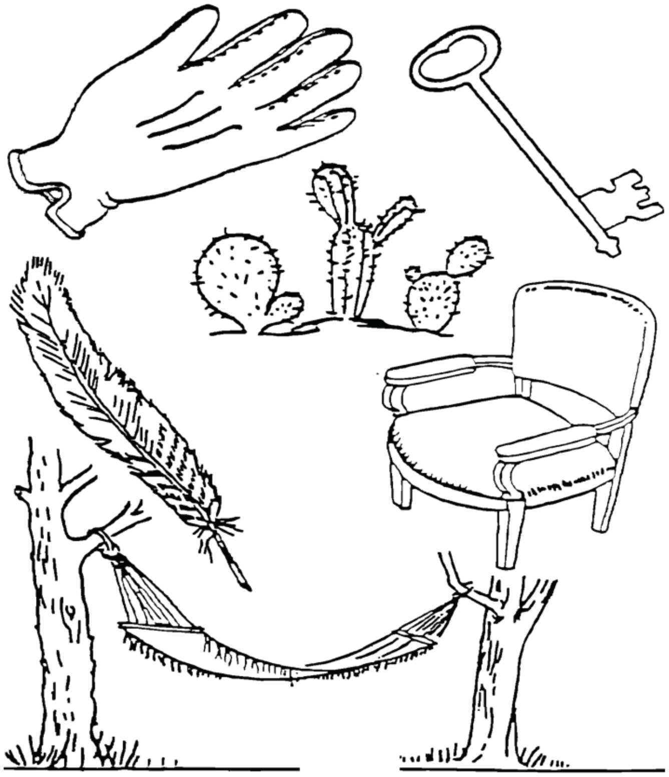

MAMA

TIP – TOP

FIFTY – FIFTY

THANKS

HUCKLEBERRY

BASEBALL PLAYER

请您读出下列单词：
妈妈
大地
飞机飞机
丝绸
按时开工
吃葡萄不吐葡萄皮

25.2 Appendix 3: EQ-5D

Subject ID ___ ___ ___ ___ ___ ___ ___ ___ ___ ___
Subject Date of Birth ___ ___/___ ___/___ ___
Hospital ID ________________________________
Date of Examination ___ ___/___ ___/___ ___

Health Questionnaire (EQ-5D-5L)

Under each heading, please tick the ONE box that best describes your health TODAY.

MOBILITY

- ☐1 I have no problems in walking about
- ☐2 I have slight problems in walking about
- ☐3 I have moderate problems in walking about
- ☐4 I have severe problems in walking about
- ☐5 I am unable to walk about

SELF-CARE

- ☐1 I have no problems washing or dressing myself
- ☐2 I have slight problems washing or dressing myself
- ☐3 I have moderate problems washing or dressing myself
- ☐4 I have severe problems washing or dressing myself
- ☐5 I am unable to wash or dress myself

USUAL ACTIVITIES *(e.g. work, study, housework, family or leisure activities)*

- ☐1 I have no problems doing my usual activities
- ☐2 I have slight problems doing my usual activities
- ☐3 I have moderate problems doing my usual activities
- ☐4 I have severe problems doing my usual activities
- ☐5 I am unable to do my usual activities

PAIN / DISCOMFORT

- ☐1 I have no pain or discomfort
- ☐2 I have slight pain or discomfort
- ☐3 I have moderate pain or discomfort
- ☐4 I have severe pain or discomfort
- ☐5 I have extreme pain or discomfort

ANXIETY / DEPRESSION

- ☐1 I am not anxious or depressed
- ☐2 I am slightly anxious or depressed
- ☐3 I am moderately anxious or depressed
- ☐4 I am severely anxious or depressed
- ☐5 I am extremely anxious or depressed

To help people say how good or bad a health state is, we have drawn a scale (rather like a thermometer) on which the best state you can imagine is marked 100 and the worst state you can imagine is marked 0.

We would like you to indicate on this scale how good or bad your own health is today, in your opinion. Please do this by drawing a line from the box below to whichever point on the scale indicates how good or bad your health state is today.

Your own health state today

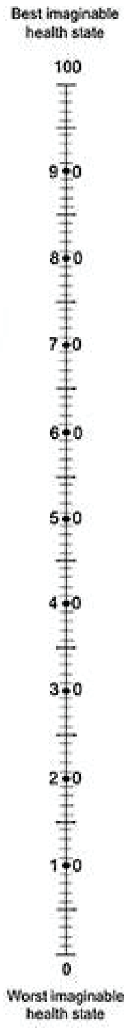

3

UK (English) © 1990 EuroQol Group EQ-5D™ is a trade mark of the EuroQol Group

Clinical study protocol

Efficacy and safety of intravenous methylprednisolone sodium succinate in acute ischemic stroke patients underwent endovascular treatment: a multicenter, randomized, double-blind trial.

Principal Investigators

Wenjie Zi, MD. and Qingwu Yang, Ph.D.

Xinqiao Hospital, Army Medical University, Chongqing, China

Prepared by:

Wenjie Zi, MD, Xinqiao Hospital, Army Medical University, Chongqing, China

Qingwu Yang, Ph.D, Xinqiao Hospital, Army Medical University, Chongqing, China

Protocol Version: 2.0

Table of Contents

1.Study Synopsis 96

2.Flow chart and schedule of assessment 106

2.1 Flow Chart 106
2.2. Schedule of Assessments 107

3.List of Abbreviations 109

4.Background 111

4.1 The burden of stroke 111
4.2 Treatment in acute ischemic stroke 111
4.3 Glucocorticoid in Stroke 113

5.Study Objectives 115

6.Product characteristics of methylprednisolone 116

6.1 Description of the investigational medicinal product 116
6.2 Indications and Usage 117
6.3 Contraindications 120
6.4 Warning 120

7.Organizational Structure 127

7.1 Funding 128
7.2 Trials Boards and Committees 128
7.2.1 Steering Committee 128
7.2.2 Data Safety and Monitoring Board 128
7.2.3 Ethics Advisory Board 129
7.2.4 Imaging Core Laboratory 129
7.2.5 Clinical Events Committee 129

8.Design 130

9.Population 130

9.1 Inclusion criteria 130
9.2 Exclusion Criteria 131
9.3 Sample Size 132
10.Imaging Training 133

11.Randomization 133

12.Blinding/Unblinding 134

13.Study Treatment 135

13.1 Formulation, Packaging, and Handling 135
13.2 Test Product 135
13.3 Comparator 135
13.4 Dosage, Administration, and Compliance 136

13.4.1 Methylprednisolone and its Placebo....136
13.4.2 Concomitant therapy....136
13.4.4 Drug Accountability....137

14.Study Schedule....137

14.1 Screening period (Visit V0, Randomization)....138
14.1.1 Visit V0....138
14.1.2 Randomization (R)....139
14.2 Treatment period (Visits V1， V2)....139
14.2.1 Visit 1....139
14.2.2 Visit 2....140
14.3 Follow Up period (Visits V3, V4)....140
14.3.1 Visit 3....141
14.3.3 Visit 4....141

15.Endpoints of the Clinical Trial....141

15.1 Efficacy Analysis-Primary Endpoint....141
15.2 Efficacy Analysis-Secondary Endpoints....142
15.3 Safety Analysis-Primary Endpoint....142
15.3.1 Primary Safety Endpoints....142
15.3.2 Secondary Safety Endpoints....143

16.Assessment....143

16.1 Assessment of efficacy....144
16.1.1 The modified Rankin Scale (mRS)....144
16.1.2 The National Institutes of Health Stroke Scale (NIHSS)....145
16.1.3 European Quality Five Dimensions Five Level scale....146
16.2 Assessment of safety....146
16.2.1 Mortality at 90±7 days....146
16.2.2 Symptomatic Intracranial Haemorrhage....146
16.2.3 Any ICH within 48 hours....147
16.2.4 Proportion of patients with pneumonia....148
16.2.4 Proportion of patients with gastrointestinal bleeding....148
16.2.5 Adverse events....148
16.2.6 Serious Adverse Event....151
16.2.7 Adverse Event Recording and Follow Up....152
16.2.8 Serious Adverse Events Reporting....153

17.Study Discontinuation Criteria....154

17.1 Patient Discontinuation....154
17.2 Study Treatment Discontinuation....155
17.3 Study and Site Discontinuation....155

18.Statistical Analysis....156

18.1 Determination of sample size....156
18.2 Analysis population....157
18.2.1 Intention-to-Treat Population....157

18.2.2. Per-Protocol Population 158
18.2.3. Safety Population 159
18.2 Analysis of Efficacy–primary endpoint 159
18.3 Analysis of Efficacy – secondary endpoints 160
18.4 Safety analysis 160
18.5 Tolerability Analysis (adverse events) 161
18.5.1 Adverse Events 161
18.5.2 Serious Adverse Events 161
18.5.3 All Adverse Events 161
18.6 Adjustment for Covariates and Subgroup Analyses 162
18.7 Missing data handling 163
19.Ethical and regulatory consideration 163
19.1 General requirements and Considerations 163
19.2 Study monitoring and quality control 164
19.3 Informed Consent 165
19.4 Confidentiality 166
19.5 Liability and Insurance 166
20.Administrative procedures 167
20.1 Curriculum vitae 167
20.2 Secrecy agreement 167
20.3 Ownership of data and use of the study results 167
20.4 Protocol amendments 167
21.Data Retention 168
22.Study report and publications 168
23.References Uncategorized References 169
24.Appendices 172
24.1 Appendix 1: mRS 172
24.2 Appendix 2: NIHSS 174
24.3 Appendix 3: EQ-5D 182

1. Study Synopsis

**Study Title**	MARVEL: Methylprednisolone for acute large vessel occlusion: a randomized double-blind, placebo-controlled trial in recanalization patients
**Study registry number**	**registry number:** ChiCTR2100051729
**Indication**	Acute Ischemic Stroke
**Study Centres**	About 80 stroke centres in China
**Number of Subjects**	1672 (836 subjects per group)
**Study Estimate Duration**	February 2022 (first patient in) until July 2023
**Study Objectives**	To test the safety and efficacy of adjunctive low-dose methylprednisolone sodium succinate therapy for the treatment of acute ischemic stroke patients within 24 hours of onset who underwent EVT.
**Study Design**	A multicentre, randomized, double-blind, placebo-controlled trial
**Study Population**	Patients with large vessels occlusive stroke within 24 hours that underwent endovascular treatment
**Inclusion Criteria**	● Age ≥ 18 years;

	● The time from onset to randomization was within 24 hours;<br>● Anterior circulation ischemic stroke was preliminarily determined according to clinical symptoms or imaging examination;<br>● Baseline Alberta Stroke Program Early CT Score (ASPECTS) ⩾ 3;<br>● Baseline National Institutes of Health Stroke Scale (NIHSS) ⩾ 6<br>● Computed tomography angiography (CTA) /magnetic resonance angiography (MRA) /digital subtraction angiography (DSA) confirmed occlusion of intracranial segment of internal carotid artery and middle cerebral artery who decided to receive endovascular treatment.<br>● Written informed consent signed by patients or their family members.
**Exclusion Criteria**	● Intracranial hemorrhage confirmed by cranial computed tomography (CT) or magnetic resonance imaging (MRI);<br>● mRS score ⩾ 2 before onset;<br>● Pregnant or lactating women;

	• Allergic to contrast agents;<br>• Allergic to glucocorticoids;<br>• Participating in other clinical trials;<br>• Systolic blood pressure > 185 mmHg or diastolic pressure > 110 mmHg, and oral antihypertensive drugs can not control;<br>• Genetic or acquired bleeding constitution, lack of anticoagulant factors; Or oral anticoagulants and INR > 1.7;<br>• Blood sugar < 2.8 mmol/L (50 mg/dl) or > 22.2 mmol/L (400 mg/dl), platelet < 90 x 10^9/L;<br>• The artery is tortuous and the thrombectomy device cannot reach the target vessel;<br>• Bleeding history (gastrointestinal and urinary tract bleeding) in recent 1 month;<br>• Chronic hemodialysis and severe renal insufficiency (glomerular filtration rate < 30 ml/min or serum creatinine > 220 umol/L [2.5 mg/ dL]);<br>• Life expectancy due to any advanced disease < 6 months;<br>• Follow-up is not expected to be completed;

	● Intracranial aneurysm and arteriovenous malformation;<br>● Brain tumors with imaging mass effect;<br>● Severe systemic infectious diseases.
**Study drug**	<u>Methylprednisolone group:</u> The box of the methylprednisolone group contains 12 bottles, each containing 40mg of methylprednisolone sodium succinate. Both the bottle and box are labelled as “Study Drug”.<br><u>Placebo group:</u> The box of the placebo group contains 12 bottles, each containing 40mg of placebo. Both the bottle and box are labelled as “Study Drug”.
**Randomization**	Eligible patients will be consecutively randomized to treatment with either Methylprednisolone or Placebo group with a ratio of 1:1 by a web-based APP (Jinlingshu) on mobile phone or computer (https://jinlingshu.com/). Randomization will be stratified by participating centres permutation block size of 4.
**Treatments**	Patients will be assigned to receive either a placebo or methylprednisolone, with a dosage of 2mg/kg (based on estimated or actual weight if known, not exceeding a maximum dose of 160 mg) per day for a duration of 3 days. The initial

	study drug will be administered as soon as possible after randomisation. It is recommended that the initial study drug administrated before arterial access closure, but it should not be delayed more than 2 hours after arterial access closure.
**Consent**	Explicit written, signed informed consent from the subject or legally authorized representative will be obtained prior to any protocol specific procedures.
**Criteria for Evaluation-Efficacy**	Primary Efficacy Endpoint<br>● Reduced disability level (ordinal shift in mRS score) at 90 ±7 days;<br>Secondary Efficacy Endpoints<br>● Proportion of patients with mRS score 0 to 4 at 90 ±7 days;<br>● Proportion of patients with mRS score 0 to 3 at 90 ±7 days;<br>● Proportion of patients with mRS score 0 to 2 at 90 ±7 days;<br>● Proportion of patients with mRS score 0 to 1 or returned to pre-stroke mobidity at 90 ±7 days;<br>● NIHSS score at 5-7 days or at early discharge;<br>● Health-related quality of life [European Quality of Life Five-Dimension visual-analogue scale (EQ-5D VAS)] at 90 ±7 days.
**Criteria for Evaluation-Safety**	Primary Safety Endpoints<br>● Mortality at 90 ±7 days;

	● Proportion of patients with symptomatic intracranial haemorrhage within 48 hours. Symptomatic intracranial haemorrhage will be adjudicated by an independent Imaging Core Laboratory according to the modified Heidelberg Bleeding Classification;<br>Secondary Safety Endpoints<br>● Proportion of patients with any radiologic intracranial haemorrhage within 48 hours;<br>● Proportion of patients with pneumonia;<br>● Proportion of patients with gastrointestinal haemorrhage within 7 days after EVT;<br>● Incidence of serious adverse events.<br>● Incidence of any adverse events.
**Sample Size Calculation**	The current trial is designed detect a shift on the modified Rankin Scale score, which represents the global disability, to a lower score. The expected distribution of the modified Rankin Scale score, derived from the EVT for acute anterior circulation ischemic stroke (ACTUAL) registry and the Highly Effective Reperfusion evaluated in Multiple Endovascular Stroke Trials (HERMES) collaboration, is as follows: 7%, 20%, 16%, 11%, 13%, 8% and 25%.

	The sample size was calculated using the cumulative proportion of patients with mRS 0-2. We assume a moderate effect of 7% absolute increase in the cumulative proportion of patients with mRS 0-2 in the intervention group, compared with controls, indicating an odds ratio (OR) of 1.33, which have substantially exceeds the minimal clinically important difference.10 In order to demonstrate the expected treatment effect with a type-1 error alpha =0.05 (two-tailed) and a power of 80% (beta = 20%). A sample size of n= 1588 patients (n=794 per treatment group) is required.<br>The intention-to-treat principle will be applied to the primary analysis and, therefore, to safeguard against dilution of the treatment effect associated with an approximate 5% non-adherence rate (due to loss to follow-up, consent withdrawal and other reasons), we initially planned to enrol n=1672 patients (n=836 per treatment group) for this study. This estimation was performed based on PASS (NCSS, LLC. Kaysville, Utah, USA) version 15.0.
**Statistical Methods**	All efficacy analyses will be conducted on data from all randomly assigned patients according to the intention-to-treat principle. All efforts will be made to minimize the amount of missing data. Sensitivity analyses based on different hypotheses

	about the missingness pattern of the primary outcome will be performed to test for the robustness of the primary analysis. Analyses will also be repeated according the per protocol principle.<br>Analysis of Efficacy - primary endpoint:<br>The primary efficacy endpoint is disability evaluated 90 (±7) days after randomization using the modified Rankin Scale (mRS) score. The primary treatment effect will be estimated as common odds ratio or using assumption-free method.<br>Covariable were age, baseline NIHSS score, pre-stroke mRS, baseline ASPECTS score, use of intravenous thrombolysis, time from onset to randomization, and occlusion location.<br>Analysis of Efficacy - Secondary endpoint:<br>Proportion of with mRS score 0 to 4 at 90 ±7 days and proportion of mRS score 0 to 3 at 90 ±7 days, proportion of patients with mRS score 0 to 2 at 90 ±7 days, proportion of mRS score 0 to 1 or return to pre-morbid mRS score at 90 days (for patients with mRS >1). Between-group differences will be tested using generalized linear models. The risk ratios and corresponding 95%CI will be provided.<br>NIHSS score at 5-7 days or at early discharge and Health-related quality of life (EQ-5D VAS). The results will be

	compared between two randomized treatment groups using GLM. The mean difference and corresponding 95%CI will be provided. Normality and variance homogeneity of residuals will be accessed graphically. The win ratio method will be used if normality assumption is seriously violated.<br>Analysis of Safety:<br>The safety analysis will be conducted on the Safety Population. Mortality due to any cause at 90 ±7 days. The difference of mortality in two treatment groups will be tested using modified Poisson Regression. In addition, the Kaplan-Meier method will be used to assess the mortality. The log-rank test will be applied to compare the two treatment groups. Cox regression model will be employed to calculate the hazard ratio with 95%CI.<br>In addition to mortality due at 90 ±7 days, SICH within 48 hours is considered to be another primary safety outcome. SICH will be evaluated according to the modified Heidelberg Bleeding Classification. Besides, modified Poisson regression will be fitted to estimate the risk ratio associated with the treatment effect. The proportion of Any radiologic ICH within 48 hours between methylprednisolone and placebo groups will be tested in the same manner as SICH.<br>Proportion of patients with pneumonia and proportion of

	patients with gastrointestinal haemorrhage within 7 days after EVT will be explore and tested the same as SICH and any ICH. Adverse events (AE) and serious adverse event (SAE) will be summarized and presented by treatment group for all patients in the Safety Population. The incidences will be compared via Fisher's exact test or chi-square test as appropriate.

2. Flow chart and schedule of assessment

2.1 Flow Chart

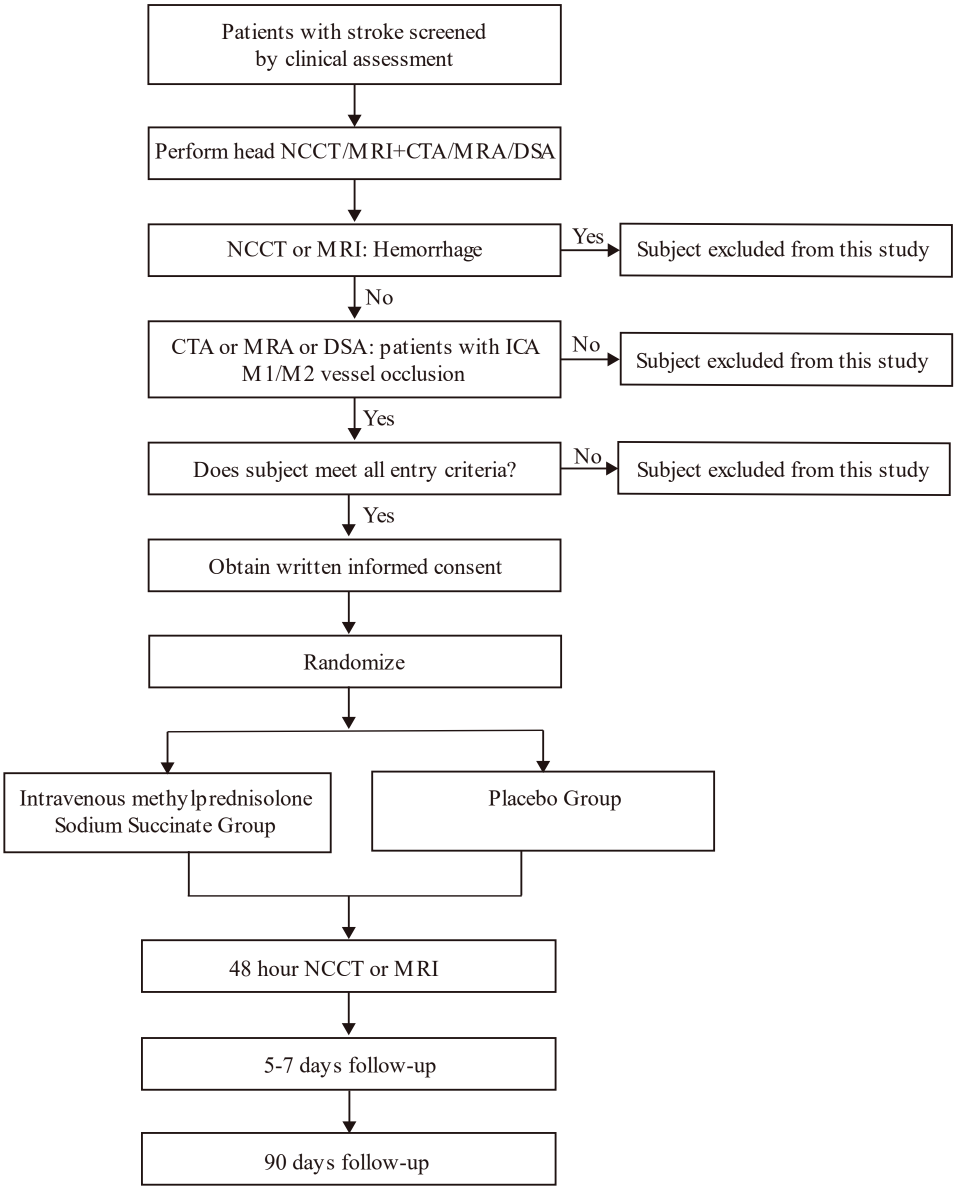

2.2. Schedule of Assessments

	**Visit (Time in hours / days from EVT =V1)**					
	Screening		Treatment		Follow Up	
	**V0**	**R**	**V1**[a] Study medication administration	**V2** (48-96h)	**V3**[b] (5-7d)	**V4** (90±7d)
Informed consent	X					
Demographic data	X					
Medical history	X					
Physical examination	X			X	X	
Pre-stroke mRS	X					
NIHSS	X				X	
mRS						X
EQ-5D						X
Previous medication	X					
Blood pressure and heart rate	X		X	X[e]	X	
Local laboratory result	X			X		
Pregnancy test[c]	X					

Brain CT/MRI plus CTA/MRA/DSA	X					
Brain CT/MRI scan[d]				X		
Inclusion / exclusion criteria	X					
Randomization		X				
Study medication			X			
Concomitant medication	X		X	X	X	X
12-lead ECG	X					
48h monitoring			X[a]			
Adverse events			X	X	X	X

V = Visit; R = Randomization; h = hour; d = day

[a] 48h monitoring in Unit or equivalent unit including repeated measurements of blood pressure, heart rate and body temperature

[b] or hospital discharge if < 5 days

[c] Mandatory for women of childbearing potential

[d] also to be performed in any case of neurological deterioration during the first 96h

[e] every hour

3. List of Abbreviations

AE	Adverse Event
AIS	Acute Ischemia Stroke
BI	Barthel Index
CRF	Case Report Form
CT	Computed Tomography
CTA	Computed Tomography Angiography
DSMB	Data Safety Monitoring Board
EAB	Ethics Advisory Board
ECG	Electrocardiogram
EQ-5D	European Quality Five-Dimension
EVT	Endovascular Therapy
GCP	Good Clinical Practice
GOS	Glasgow outcome scale
HBC	Heidelberg bleeding classification
ICH	Intracerebral Haemorrhage
ICH-GCP	International Conference on Harmonization-Good Clinical Practice
IRB	Institutional Review Board
ITT	Intention-to-Treat
IVT	Intravenous Thrombolysis
LVO	Large Vessel Occlusion
MARVEL	MARVEL: Methylprednisolone for acute large vessel occlusion: a

	randomized double-blind, placebo-controlled trial in revascularization patients
MedDRA	Medical Dictionary for Regulatory Activities
MR	Magnetic Resonance
MRA	Magnetic Resonance Angiography
mRS	modified Rankin Scale
NIHSS	National Institutes of Health Stroke Scale
NINDS	National Institute of Neurological Disorders and Stroke
PI	Principal Investigator
PP	Per-Protocol
REB	Research Ethics Board
RISS	Rapidly improving stroke symptoms
RR	Risk ratio
rt-PA	recombinant tissue Plasminogen Activator
SAB	Scientific Advisory Board
SAE	Serious Adverse Event
SAP	Statistical Analysis Plan
SICH	Symptomatic Intracerebral Haemorrhage
SOC	System Organ Class

4. Background

4.1 The burden of stroke

Stroke remains the second-leading cause of death and the third-leading cause of death and disability combined in the world.[1-3] The estimated global cost of stroke is over US $721 billion (0.66% of the global GDP).[3] In the past 20 years, the burden increased substantially, with the bulk of the global stroke burden residing in lower-income and lower-middle-income countries.[3] Recently, the National Epidemiological Survey of Stroke in China (NESS-China) suggested that the current stroke prevalence (1115/100 000 [95%CI, 997–1233]) in China appears to be the highest among other low- to middle-income countries (range from 536 to 1040/100 000), but significantly lower than that observed in high-income countries (range from 2600 to 8000/100 000). [4]

4.2 Treatment in acute ischemic stroke

Stroke is normally classified as either ischemic or haemorrhagic stroke although 62.4% of cases belong to ischemic nature. Ischemic stroke is characterized by a high incidence, high disability rate, and high mortality rate, and has become the leading cause of death in China.[4] Currently, there are about 2.4 million new stroke patients and 1.1 million deaths in China each year, imposing a heavy burden on society and families.[4] Approximately 40% of acute ischemic strokes are caused acute large vessel occlusion (LVO), with anterior circulation LVO accounting for about 80% and posterior circulation LVO accounting for about 20%.[4]

Early restoration of blood flow and salvage of ischemic penumbra are the current theoretical basis of the treatment of AIS.[5,6] The current standard therapy for acute ischemic stroke including the intravenous thrombolysis and endovascular treatment.[6] Currently, the recanalization rate of acute anterior circulation large vessel occlusive stroke has been improved to 80%~94.0% in the endovascular treatment era.[7]

However, the successful recanalization rates have not been completely translated into clinical benefits for AIS patients, and the proportion of patients with good outcome was less than 50%, which is clinically referred to as "Futile recanalization (FR)" or “Ineffective reperfusion”.[8,9] That is, successful recanalization of occluded vessels (defined as modified treatment in cerebral ischemia (mTICI) blood flow grade ≥2b) does not lead to good functional outcomes (mRS score ≥3 at 90 days). Moreover, reperfusion of the ischaemic tissue is not risk-free, often causes haemorrhagic transformation (HT), blood–brain barrier (BBB) disruption.[10-12] Besides, malignant brain oedema was a common complication that were associated with high mortality.[13,14]

Several mechanisms may contribute futile reperfusion and may be the potential treatment target for the futile reperfusion phenomenon: the non-reflow phenomenon, cerebral oedema, blood-brain barrier damage and inflammatory damage.[12]

Firstly, the "non-reflow" phenomenon refers to tissue hypoperfusion despite the timely or complete recanalization of an occluded artery.[15] This phenomenon is frequently observed in clinical practice, with a prevalence of more than half in cases of coronary

artery occlusion, and it serves as an independent predictor of adverse outcomes.[16] In the cerebral circulation, non-reflow has consistently been demonstrated in preclinical studies since first being described in 1968.[15] Non-reflow in the brain may involve post-ischemic swelling in endothelial cells, microvasculature occluded by platelet and neutrophils, interstitial oedema, and inflammatory reaction.[17] Recent experimental data further suggest that non-reflow is present during the first 24 hours after recanalization.[17] In clinical studies, the prevalence of the no-reflow phenomenon after endovascular treatment (EVT) in acute ischemic stroke was approximately 30%, and this number is expected to increase with the extension of EVT to a 24-hour time window.[17] Secondly, the endovascular treatment has a damage to BBB, and will lead to severe brain edema and haemorrhagic transformation that considerably contribute to neurological deterioration and death. [11,18] Thirdly, inflammatory mechanisms after stroke are now increasingly considered prime targets for stroke therapy since immune signals and their mediators can have both detrimental and beneficial effects at different stages of the disease process.[19,20]

Thus, due to the multi-faceted nature of the aforementioned mechanisms, single-target agents may not sufficiently intervene. Therefore, combinations of agents or multi-target agents may be necessary to address these mechanisms effectively.

4.3 Glucocorticoid in Stroke

In animal models, corticosteroids have shown the ability to reduce infarct size, regulate cerebral blood flow, enhance non-reflow phenomenon, stabilize the blood-brain barrier,

prevent angiogenic edema, and modulate immune response.[21-29], while none have been adopted through clinical trial success in acute ischemic stroke therapy.[30-32]

Several lessons are learned from prior studies. Firstly, those trials were performed in the era when reperfusion strategies were not well established. The pre-clinical study of corticosteroids has suggested that corticosteroid was effective in the transient middle cerebral artery occlusion(tMCAO) model but was ineffective in the permanent middle cerebral artery occlusion(pMCAO) model.[22] It suggested that reperfusion or not was an important condition for the effect of corticosteroids.[22,33-35] In past corticosteriod treatment trials, conducted before the advent of mechanical thrombectomy as a treatment option, it is likely that many patients failed to reperfuse; the candidate adjuvant treatments were tested in the more challenging setting of permanent rather than transient brain ischemia and thus failed to show benefit in clinical trials.[35] The STAIR (Stroke Treatment Academic Industry Roundtable) suggested testing the neuroprotective agents in the new endovascular treatment era when the reperfusion rate can be up to 90%. [33,36] [37] Secondly, most included patients with “presumed ischemic stroke” were treated in an era where reperfusion was not effective, and the sample size of patients were small in the previous trials. Only 8/24 published trials of corticosteroids in stroke were acceptable for further analysis, and these comprised woefully inadequate numbers of patients (only 466). Davis and Donnan suggested that steroid therapy for stroke be discarded prematurely. Thirdly, most trials used a high-dose and long duration of corticosteroid thus increasing the incidence of major gastrointestinal hemorrhage and infections. [30]

Therefore, there was a call to reignite the study of corticosteroids in stroke, especially in the reperfusion era. [37,38] One small sample size trial in China has studied the use of corticosteroids in the new endovascular treatment era and have yielded promising result that a short course and low-dose corticosteroid can improve the functional outcome, which was consistent with the preclinical study.[39] The surprising result indicated that low-dose and short-term corticosteroids may have an effect for patients with ischemic stroke in the new EVT era. A ray of hope comes to restudy the effect of corticosteroids in the EVT era. Trials conducted on patients who underwent endovascular treatment closely aligned with the primate model of transient middle cerebral artery occlusion, where the corticosteroid has demonstrated its efficacy. This is in contrast to the permanent middle cerebral artery occlusion model, where the corticosteroid has shown its benefit.[22]

As an intermediate-acting corticosteroid with its minimal side effects and its ability to effectively cross the blood-brain barrier, methylprednisolone is widely used in clinical practice. We propose a multicenter, prospective, randomized, double-blind, placebo-controlled trial to investigate the efficacy and safety of adjunctive methylprednisolone for patients underwent endovascular treatment. The main objective of this trial is to determine whether the early administration of methylprednisolone can improve the clinical outcomes of patients with acute anterior circulation large vessel occlusive stroke after successful recanalization.

5. Study Objectives

The purpose of MARVEL trial is to investigate the efficacy and safety of early adjunctive methylprednisolone in AIS patients underwent EVT in a multicenter, randomized, double-blind, placebo-controlled trial. The findings of MARVEL are likely to have a direct impact on clinical practice. In case of a positive result, this will provide evidence for an effective, safe, and affordable treatment in the new EVT era. Additionally, this trial will contribute high-quality evidence on the use of corticosteroids in stroke through a large-scale, multicenter trial, addressing existing gaps in current knowledge.

6. Product characteristics of methylprednisolone

The following is the summary of product characteristics for methylprednisolone provided by the Chongqing Lummy Pharmaceutical Co., Ltd., Chongqing, China.

6.1 Description of the investigational medicinal product

Methylprednisolone sodium succinate, is the sodium succinate ester of methylpred-nisolone, and it occurs as a white, or nearly white, odorless hygroscopic, amorphous solid. It is very soluble in water and in alcohol; it is insoluble in chloroform and is very slightly soluble in acetone.

The chemical name for methylprednisolone sodium succinate is pregna-1,4-diene-3,20dione,21-(3-carboxy-1-oxopropoxy)-11,17-dihydroxy-6-methyl-monosodium salt, (6α, 11β), and the molecular weight is 496.53. The structural formula is represented below:

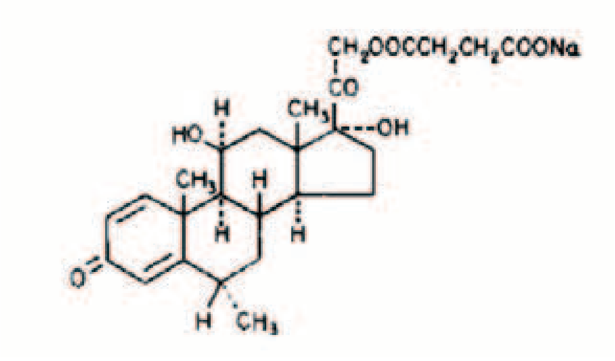

Methylprednisolone sodium succinate is soluble in water; it may be administered in a small volume of diluent and is well suited for intravenous use in situations where high blood levels of methylprednisolone are required rapidly.

CLINICAL PHARMACOLOGY

Glucocorticoids, naturally occurring and synthetic, are adrenocortical steroids that are readily absorbed from the gastrointestinal tract. Naturally occurring glucocorticoids (hydrocortisone and cortisone), which also have salt-retaining properties, are used as replacement therapy in adrenocortical deficiency states. Their synthetic analogs are primarily used for their potent anti-inflammatory effects in disorders of many organ systems. Glucocorticoids cause profound and varied metabolic effects. In addition, they modify the body's immune responses to diverse stimuli. Methylprednisolone is a potent anti-inflammatory steroid with greater anti-inflammatory potency than prednisolone and even less tendency than prednisolone to induce sodium and water retention. Methylprednisolone sodium succinate has the same metabolic and anti-inflammatory actions as methylprednisolone.

6.2 Indications and Usage

When oral therapy is not feasible, and the strength, dosage form, and route of administration of the drug reasonably lend the preparation to the treatment of the condition, the intravenous or intramuscular use of Methylprednisolone is indicated as follows:

Allergic states: Control of severe or incapacitating allergic conditions intractable to adequate trials of conventional treatment in asthma, atopic dermatitis, contact dermatitis, drug hypersensitivity reactions, perennial or seasonal allergic rhinitis, serum sickness, transfusion reactions.

Dermatologic diseases: Bullous dermatitis herpetiformis, exfoliative erythroderma, mycosis fungoides, pemphigus, severe erythema multiforme (Stevens-Johnson syndrome).

Endocrine disorders: Primary or secondary adrenocortical insufficiency (hydrocortisone or cortisone is the drug of choice; synthetic analogs may be used in conjunction with mineralocorticoids where applicable; in infancy, mineralocorticoid supplementation is of particular importance), congenital adrenal hyperplasia, hypercalcemia associated with cancer, nonsuppurative thyroiditis.

Gastrointestinal diseases: To tide the patient over a critical period of the disease in regional enteritis (systemic therapy) and ulcerative colitis. Hematologic disorders: Acquired (autoimmune) hemolytic anemia, congenital (erythroid) hypoplastic anemia (Diamond-Blackfan anemia), idiopathic thrombocytopenic purpura in adults

(intravenous administration only; intramuscular administration is contraindicated), pure red cell aplasia, selected cases of secondary thrombocytopenia.

Miscellaneous: Trichinosis with neurologic or myocardial involvement, tuberculous meningitis with subarachnoid block or impending block when used concurrently with appropriate antituberculous chemotherapy.

Neoplastic diseases: For the palliative management of leukemias and lymphomas.

Nervous System: Acute exacerbations of multiple sclerosis; cerebral edema associated with primary or metastatic brain tumor, or craniotomy.

Ophthalmic diseases: Sympathetic ophthalmia, uveitis and ocular inflammatory conditions unresponsive to topical corticosteroids. Renal diseases: To induce diuresis or remission of proteinuria in idiopathic nephrotic syndrome or that due to lupus erythematosus.

Respiratory diseases: Berylliosis, fulminating or disseminated pulmonary tuberculosis when used concurrently with appropriate antituberculous chemotherapy, idiopathic eosinophilic pneumonias, symptomatic sarcoidosis.

Rheumatic disorders: As adjunctive therapy for short-term administration (to tide the patient over an acute episode or exacerbation) in acute gouty arthritis; acute rheumatic carditis; ankylosing spondylitis; psoriatic arthritis; rheumatoid arthritis, including juvenile rheumatoid arthritis (selected cases may require low-dose maintenance therapy). For the treatment of dermatomyositis, temporal arteritis, polymyositis, and systemic lupus erythematosus.

6.3 Contraindications

The methylprednisolone sodium succinate is contraindicated:

- in systemic fungal infections and patients with known hypersensitivity to the product and its constituents.
- for intrathecal administration. Reports of severe medical events have been associated with this route of administration.

6.4 Warning

Serious Neurologic Adverse Reactions with Epidural Administration

Serious neurologic events, some resulting in death, have been reported with epidural injection of corticosteroids. Specific events reported include, but are not limited to, spinal cord infarction, paraplegia, quadriplegia, cortical blindness, and stroke. These serious neurologic events have been reported with and without use of fluoroscopy. The safety and effectiveness of epidural administration of corticosteroids have not been established, and corticosteroids are not approved for this use.

GENERAL

Increased dosage of rapidly acting corticosteroids is indicated in patients on corticosteroid therapy who are subjected to any unusual stress before, during, and after the stressful situation.

Results from one multicenter, randomized, placebo-controlled study with methylprednisolone hemisuccinate, an intravenous corticosteroid, showed an increase in early (at 2 weeks) and late

(at 6 months) mortality in patients with cranial trauma who were determined not to have other clear indications for corticosteroid treatment. High doses of systemic corticosteroids, should not be used for the treatment of traumatic brain injury.

Cardio-renal

Average and large doses of corticosteroids can cause elevation of blood pressure, salt and water retention, and increased excretion of potassium. These effects are less likely to occur with the synthetic derivatives except when used in large doses. Dietary salt restriction and potassium supplementation may be necessary. All corticosteroids increase calcium excretion. Literature reports suggest an apparent association between the use of corticosteroids and left ventricular free wall rupture after a recent myocardial infarction; therefore, therapy with corticosteroids should be used with great caution in these patients.

Endocrine

Hypothalamic-pituitary adrenal (HPA) axis suppression, Cushing's syndrome, and hyperglycemia. Monitor patients for these conditions with chronic use.

Corticosteroids can produce reversible HPA axis suppression with the potential for glucocorticosteroid insufficiency after withdrawal of treatment. Drug induced secondary adrenocortical insufficiency may be minimized by gradual reduction of dosage. This type of relative insufficiency may persist for months after discontinuation of therapy; therefore, in any situation of stress occurring during that period, hormone therapy should be reinstituted.

Drug-Induced Liver Injury

Rarely, high doses of cyclically pulsed intravenous methylprednisolone (usually for the treatment of exacerbations of multiple sclerosis at doses of 1 gram/day) can induce a toxic form of acute hepatitis. The time to onset of this form of steroid-induced liver injury can be several weeks or longer. Resolution has been observed after discontinuation of treatment. However, serious liver injury can occur, sometimes resulting in acute liver failure and death. Discontinue intravenous methylprednisolone if toxic hepatitis occurs. Since recurrence has occurred after re-challenge, avoid use of high dose intravenous methylprednisolone in patients with a history of toxic hepatitis caused by methylprednisolone.

Infections

General

Patients who are on corticosteroids are more susceptible to infections than are healthy individuals. There may be decreased resistance and inability to localize infection when corticosteroids are used. Infections with any pathogen (viral, bacterial, fungal, protozoan, or helminthic) in any location of the body may be associated with the use of corticosteroids alone or in combination with other immunosuppressive agents. These infections may be mild, but can be severe and at times fatal. With increasing doses of corticosteroids, the rate of occurrence of infectious complications increases. Corticosteroids may also mask some signs of current infection. Do not use intraarticularly, intrabursally or for intratendinous administration for local effect in the presence of acute local infection. A study has failed to establish the efficacy of methylprednisolone sodium succinate in the treatment of sepsis syndrome and septic

shock. The study also suggests that treatment of these conditions with methylprednisolone sodium succinate may increase the risk of mortality in certain patients (i.e., patients with elevated serum creatinine levels or patients who develop secondary infections after methylprednisolone sodium succinate).

Fungal infections

Corticosteroids may exacerbate systemic fungal infections and therefore should not be used in the presence of such infections unless they are needed to control drug reactions. There have been cases reported in which concomitant use of amphotericin B and hydrocortisone was followed by cardiac enlargement and congestive heart failure (see CONTRAINDICATIONS and PRECAUTIONS, Drug Interactions, Amphotericin B injection and potassium-depleting agents). Special pathogens Latent disease may be activated or there may be an exacerbation of intercurrent infections due to pathogens, including those caused by Amoeba, Candida, Cryptococcus, Mycobacterium, Nocardia, Pneumocystis, Toxoplasma. It is recommended that latent amebiasis or active amebiasis be ruled out before initiating corticosteroid therapy in any patient who has spent time in the tropics or in any patient with unexplained diarrhea. Similarly, corticosteroids should be used with great care in patients with known or suspected Strongyloides (threadworm) infestation. In such patients, corticosteroid-induced immunosuppression may lead to Strongyloides hyperinfection and dissemination with widespread larval migration, often accompanied by severe enterocolitis and potentially fatal gram-negative septicemia. Corticosteroids should not be used in cerebral malaria. There is currently no evidence of benefit from

steroids in this condition.

Tuberculosis

The use of corticosteroids in active tuberculosis should be restricted to those cases of fulminating or disseminated tuberculosis in which the corticosteroid is used for the management of the disease in conjunction with appropriate antituberculous regimen. If corticosteroids are indicated in patients with latent tuberculosis or tuberculin reactivity, close observation is necessary as reactivation of the disease may occur. During prolonged corticosteroid therapy, these patients should receive chemoprophylaxis.

Vaccination

Administration of live or live, attenuated vaccines is contraindicated in patients receiving immunosuppressive doses of corticosteroids. Killed or inactivated vaccines may be administered. However, the response to such vaccines cannot be predicted. Immunization procedures may be undertaken in patients receiving corticosteroids as replacement therapy, e.g., for Addison's disease.

Viral infections

Chicken pox and measles can have a more serious or even fatal course in pediatric and adult patients on corticosteroids. In pediatric and adult patients who have not had these diseases, particular care should be taken to avoid exposure. The contribution of the underlying disease and/or prior corticosteroid treatment to the risk is also not known. If exposed to chicken pox, prophylaxis with varicella zoster immune globulin (VZIG) may be indicated. If exposed to measles, prophylaxis with immunoglobulin

(IG) may be indicated. (See the respective package inserts for complete VZIG and IG prescribing information.) If chicken pox develops, treatment with antiviral agents should be considered.

Neurologic

Reports of severe medical events have been associated with the intrathecal route of administration (see ADVERSE REACTIONS, Gastrointestinal and Neurologic/Psychiatric).

Ophthalmic

Use of corticosteroids may produce posterior subcapsular cataracts, glaucoma with possible damage to the optic nerves, and may enhance the establishment of secondary ocular infections due to bacteria, fungi, or viruses. The use of oral corticosteroids is not recommended in the treatment of optineuritis and may lead to an increase in the risk of new episodes. Corticosteroids should be used cautiously in patients with ocular herpes simplex because of corneal perforation. Corticosteroids should not be used in active ocular herpes simplex.

PRECAUTIONS

General

This product, like many other steroid formulations, is sensitive to heat. Therefore, it should not be autoclaved when it is desirable to sterilize the exterior of the vial. The lowest possible dose of corticosteroid should be used to control the condition under treatment. When reduction in dosage is possible, the reduction should be gradual. Since complications of treatment with glucocorticoids are dependent on the size of the

dose and the duration of treatment, a risk/benefit decision must be made in each individual case as to dose and duration of treatment and as to whether daily or intermittent therapy should be used. Kaposi's sarcoma has been reported to occur in patients receiving corticosteroid therapy, most often for chronic conditions. Discontinuation of corticosteroids may result in clinical improvement.

Cardio-renal

As sodium retention with resultant edema and potassium loss may occur in patients receiving corticosteroids, these agents should be used with caution in patients with congestive heart failure, hypertension, or renal insufficiency.

Endocrine

Drug-induced secondary adrenocortical insufficiency may be minimized by gradual reduction of dosage. This type of relative insufficiency may persist for months after discontinuation of therapy; therefore, in any situation of stress occurring during that period, hormone therapy should be reinstituted. Metabolic clearance of corticosteroids is decreased in hypothyroid patients and increased in hyperthyroid patients. Changes in thyroid status of the patient may necessitate adjustment in dosage.

Gastrointestinal

Steroids should be used with caution in active or latent peptic ulcers, diverticulitis, fresh intestinal anastomoses, and nonspecific ulcerative colitis, since they may increase the risk of a perforation. Signs of peritoneal irritation following gastrointestinal perforation in patients receiving corticosteroids may be minimal or absent. There is an enhanced effect due to decreased metabolism of corticosteroids in

patients with cirrhosis.

Musculoskeletal

Corticosteroids decrease bone formation and increase bone resorption both through their effect on calcium regulation (i.e., decreasing absorption and increasing excretion) and inhibition of osteoblast function. This, together with a decrease in the protein matrix of the bone secondary to an increase in protein catabolism, and reduced sex hormone production, may lead to inhibition of bone growth in pediatric patients and the development of osteoporosis at any age. Special consideration should be given to patients at increased risk of osteoporosis (i.e., postmenopausal women) before initiating corticosteroid therapy. Local injection of a steroid into a previously infected site is not usually recommended.

Neurologic-Psychiatric

Although controlled clinical trials have shown corticosteroids to be effective in speeding the resolution of acute exacerbations of multiple sclerosis, they do not show that corticosteroids affect the ultimate outcome or natural history of the disease. The studies do show that relatively high doses of corticosteroids are necessary to demonstrate a significant effect. An acute myopathy has been observed with the use of high doses of corticosteroids, most often occurring in patients with disorders of neuromuscular transmission (e.g., myasthenia gravis), or in patients receiving concomitant therapy with neuromuscular blocking drugs (e.g., pancuronium). This acute myopathy is generalized, may involve ocular and respiratory.

7. Organizational Structure

7.1 Funding

MARVEL trial is an investigator-initiated study which is organized by the second affiliated hospital of the Army Medical University and conducted in 82 centres in China. MARVEL will be funded by the National Natural Science Foundation of China. Methylprednisolone and its placebo are manufactured and provided by Chongqing Lummy Pharmaceutical Co., Ltd., Chongqing, China. The funder had no involvement in the study design, data collection, analysis and interpretation, writing or decision to submit the paper.

7.2 Trials Boards and Committees

The following boards and institutions will assure the success of the clinical trial:

7.2.1 Steering Committee

The Steering Committee of the clinical trial will decide on the final protocol and oversee the trial.

7.2.2 Data Safety and Monitoring Board

The independent Date and Safety Monitoring Board (DSMB) will regularly monitor the safety of the trial and ensure the safety of patients at all stages of MARVEL. The independent DSMB will be composed of an experienced neurologist, a neuroradiologist, and a biostatistician, who are not participants of the MARVEL consortium and not involved in the clinical trial in any other way. The DSMB may at

any time propose to revise the clinical trial protocol or terminate the trial in case of safety concerns.

7.2.3 Ethics Advisory Board

The Ethics Advisory Board (EAB) will supervise the trial and ensure that the trial is conducted in accordance with the ICH-GCP guidelines and international and China legislation. The EAB may at any time propose to revise the clinical trial protocol or terminate the trial in case of ethical concerns.

7.2.4 Imaging Core Laboratory

Centralized imaging core laboratories will centrally blinded review all images and provide reference judgements for the definition of the per protocol population and intracranial haemorrhages. CT/MR and angiographic images will be independently reviewed by two independent central imaging core laboratories respectively. Imaging Core Laboratory will be independent to ensure it is blinded to the treatment allocation.

7.2.5 Clinical Events Committee

The Clinical Events Committee will be comprised of three expert physicians independent of the investigational sites. This committee will validate all the complications that occur over the course of the study and categorize each for severity and relatedness according to the definition in the AE section. The Clinical Events Committee can request any additional source information and images supporting the AEs to assist with the adjudication.

8. Design

MARVEL is a multicenter, randomized, double-blind, placebo-controlled trial. Patients will be randomized 1:1 to either Methylprednisolone Group or Placebo Group. The study has been registered at Chinese Clinical Trial Registry (www.chictr.org.cn, unique identifier ChiCTR2100051729).

9. Population

The patients for this trial will be recruited from AIS patients with large vessel occlusion within 24 hours from last known well.

9.1 Inclusion criteria

- Age ⩾ 18 years;
- The time from onset to randomization was within 24 hours;
- Anterior circulation ischemic stroke was preliminarily determined according to clinical symptoms or imaging examination;
- Baseline National Institutes of Health Stroke Scale (NIHSS) ⩾ 6
- Baseline Alberta Stroke Program Early CT Score (ASPECTS) ⩾ 3;
- Computed tomography angiography (CTA) /magnetic resonance angiography (MRA) /digital subtraction angiography (DSA) confirmed occlusion of intracranial segment of internal carotid artery and middle cerebral artery, and decided to undergo endovascular therapy;

- Written informed consent signed by patients or their family members.

9.2 Exclusion Criteria

Patients meeting any of the following criteria will be excluded from study enrolment.

- Intracranial hemorrhage confirmed by cranial computed tomography (CT) or magnetic resonance imaging (MRI);
- mRS score ≥ 2 before onset;
- Pregnant or lactating women;
- Allergic to contrast agents;
- Allergic to glucocorticoids;
- Participating in other clinical trials;
- Systolic blood pressure > 185 mmHg or diastolic pressure > 110 mmHg, and oral antihypertensive drugs can not control;
- Genetic or acquired bleeding constitution, lack of anticoagulant factors; Or oral anticoagulants and INR > 1.7;
- Blood sugar < 2.8 mmol/L (50 mg/dl) or > 22.2 mmol/L (400 mg/dl), platelet < 90 x 10^9/L;
- The artery is tortuous so that the thrombectomy device cannot reach the target vessel;
- Bleeding history (gastrointestinal and urinary tract bleeding) in recent 1 month;

- Chronic hemodialysis and severe renal insufficiency (glomerular filtration rate < 30 ml/min or serum creatinine > 220 umol/L [2.5 mg/ dL]);
- Life expectancy due to any advanced disease < 6 months;
- Follow-up is not expected to be completed;
- Intracranial aneurysm and arteriovenous malformation;
- Brain tumors with imaging mass effect;
- Severe systemic infectious diseases.

9.3 Sample Size

The current trial is designed detect a shift on the modified Rankin Scale score, which represents the global disability, to a lower score. The expected distribution of the modified Rankin Scale score, derived from the EVT for acute anterior circulation ischemic stroke (ACTUAL) registry[40] and the Highly Effective Reperfusion evaluated in Multiple Endovascular Stroke Trials (HERMES) collaboration[7], is as follows: 7%, 20%, 16%, 11%, 13%, 8% and 25%.

The sample size was calculated using the cumulative proportion of patients with mRS 0-2. We assume a moderate effect of 7% absolute increase in the cumulative proportion of patients with mRS 0-2 in the intervention group, compared with controls, indicating an odds ratio (OR) of 1.33, which have substantially exceeds the minimal clinically important difference.[41,42] In order to demonstrate the expected

treatment effect with a type-1 error alpha =0.05 (two-tailed) and a power of 80% (beta = 20%). A sample size of n= 1588 patients (n=794 per treatment group) is required.

The intention-to-treat principle will be applied to the primary analysis and, therefore, to safeguard against dilution of the treatment effect associated with an approximate 5% non-adherence rate (due to loss to follow-up, consent withdrawal and other reasons), we initially planned to enrol n=1672 patients (n=836 per treatment group) for this study. This estimation was performed based on PASS (NCSS, LLC. Kaysville, Utah, USA) version 15.0.

10. Imaging Training

Investigators involved in image reading within the trial will participate in a standardized image reading training before study start.

11. Randomization

Randomization will be done by a web-based APP (Jinlingshu) on mobile phone or computer (https://jinlingshu.com/). The automated system will assign an appropriate set of study medication to each patient. Eligible patients are randomized to treatment with either intravenous methylprednisolone or placebo with a ratio of 1:1. Randomization will be stratified by participating centre with permutation block size of 4. Randomization will be completely concealed by having both web-based real-time allocation and identical appearance of methylprednisolone sodium succinate and placebo bottles. (All bottles will have a unique number. Subjects will be assigned a

random serial number according to the time they were enrolled, and corresponding masked medications will be provided).

The randomization list will be prepared by the independent statistical centre using SAS 9.4. It will only be sent to the data coordination centre responsible for central randomization and data management, the pharmacy centre responsible for treatment packaging.

12. Blinding/Unblinding

Both patient and investigator are blinded to treatment assignment. If there is a clinical situation that the investigator believes it is necessary to unblind for the safety of patients, the medical monitor must be immediately notified to discuss the intended unblinding. Premature breaking of the blind should be restricted to the setting where identification of the type of treatment is critical for adequate treatment of the patient. All unblinded patients will remain in the study and complete all follow-up visits.

If the site principal investigator wants to know the identity of the study drug for any other reason, he or she must call the medical monitor. Unless the circumstances are deemed necessary by site principal investigator, treatment codes should not be broken. The site principal investigator should document and make an explanation for any premature unblinding.

For the purpose of regulatory reporting, if required by the local health authorities, treatment codes could be broken for all serious, unexpected suspected adverse reactions that are deemed by the investigator to be related to study drug.

13. Study Treatment

13.1 Formulation, Packaging, and Handling

Methylprednisolone sodium succinate and its placebo are manufactured by Lummy Pharmaceutical Group Co., Ltd., Chongqing, China. All study medication will be manufactured, tested, released, and shipped according to Good Manufacturing Practice guidelines. Labelling and packaging of study medication will be conducted according to Good Clinical Practice and Good Manufacturing Practice guidelines, and any national regulatory requirements.

Methylprednisolone sodium succinate and its corresponding placebos are provided in numbered and are visually identical, except for a unique number. Methylprednisolone sodium succinate and it's placebo will be packed in glass bottle of identical appearance. Each kit has a unique identification number and will be stored in a safe location at room temperature on the clinical site with limited access.

13.2 Test Product

Each kit of the methylprednisolone group contains l2 bottles, and each bottles contains 40mg methylprednisolone sodium succinate, labelled for “Study Drug” and “clinical trial use only”). Each kit will be stored in a safe location at room temperature (25°C with excursions permitted between 15°C−30°C) on the clinical site with restricted access.

13.3 Comparator

Each kit of the placebo group contains l2 bottles, and each bottles contains 40mg placebo, labelled for "Study Drug" and "clinical trial use only"). Each kit will be stored in a safe location at room temperature (25°C with excursions permitted between 15°C−30°C) on the clinical site with restricted access.

13.4 Dosage, Administration, and Compliance

Eligible patients will be randomly assigned a number corresponding to a blinded sealed medication kit that sent to each patient. It is recommended to start using the study drug within 15 minutes after randomization.

13.4.1 Methylprednisolone and its Placebo

Patients will be assigned to receive either a placebo or methylprednisolone, with a dosage of 2mg/kg (based on estimated or actual weight if known, not exceeding a maximum dose of 160 mg) per day for a duration of 3 days. The initial study drug will be administered as soon as possible after randomisation. It is recommended that the initial study drug administrated before arterial access closure, but it should not be delayed more than 2 hours after arterial access closure. All patients will be treated according to the current Chinese Stroke Association guidelines for clinical management of cerebrovascular disorders[43]. Risk factors such as diabetes mellitus, hypertension, hyperlipidaemia, hyperhomocysteinaemia, obesity, drinking and smoking should be managed appropriately.

13.4.2 Concomitant therapy

Administration of any other (intravenous or oral) corticosteroids (E.g.,

Hydrocortisone) is not allowed during the first 72 hours post randomization. Gastrointestinal prophylaxis and hyperglycemia treatment will be administered per standard local protocols. Any concomitant medication within one week prior to screening through the study completion/discontinuation visit should be documented in the Concomitant Medications Case Report Form.

13.4.4 Drug Accountability

The investigational sites will be provided with sufficient amounts of investigational medicinal products. The investigational medicinal products must not be used outside the study protocol. The investigator or authorized staff is obliged to acknowledge receipt of the study medication to confirm the content and shipping temperature. Any damaged shipment of study medications will be replaced. The study drug will be disposed of in accordance with the standard operating procedures at the study site, or returned to the sponsor with appropriate documents. The investigational site's method of study medication destruction must be agreed to by the Sponsor.

Before destroying any study medications, the investigational site must obtain the written authorization from the sponsor and document the destruction of study medications in an appropriate form. Accurate records of receipt, dispensation, return and disposition of all study medication during the study should be documented on the drug inventory log.

14. Study Schedule

The schedule of assessments conducted during the study is shown in Part 2 of Chapter

2. Flow Chart and Schedule of Assessments. The study includes three periods: Screening (V0), treatment (V1, V2) and Follow up (V3, V4).

14.1 Screening period (Visit V0, Randomization)

14.1.1 Visit V0

For each eligible patient, written informed consent for participation in the study will be obtained from the patient or the patient’s legal representative prior to the performance of any protocol-related investigation. Once written informed consent is obtained, the results of the following standard of care assessments will be used for the study. The investigator will record the details of all patients consented to confirm eligibility and record the reasons for screening failure.

- Demographics
- Medical history
- Physical examination
- Determination of the pre-stroke mRS via interview of the patient or the patient’s kin
- NIHSS score (Note: performed by an NIHSS-certified practitioner)
- Collection of information on previous medications
- Measurement of blood pressure and heart rate
- Pregnancy test (urine test, in non-menopausal women)

- Laboratory tests
- A 12-lead ECG
- Non-contrast CT scan or MRI scan and CTA, MRA, or DSA
- Concomitant medications
- Checking of inclusion and exclusion criteria

14.1.2 Randomization (R)

If all inclusion criteria are met and no exclusion criteria are present, randomization will be performed.

14.2 Treatment period (Visits V1，V2)

- After randomization, the treatment period starts, including study medication (V1) and post-treatment clinical examination (V2).

14.2.1 Visit 1

Before administration of the study medication, measurement of blood pressure and heart rate have to be performed. The study medication will be administered as specified for intravenous methylprednisolone. During the administration of study medication, the following assessments will be made:

- Measurement of blood pressure and heart rate every hour.
- Documentation of any concomitant medication.
- Assessment of Adverse Event.

- In any case of significant neurological deterioration judged by the investigator, an assessment of the neurological deficit and another non-contrast cranial CT scan or MRI scan have to be performed to determine whether intracranial haemorrhage occurs.
- The 48h monitoring should be performed in Stroke Unit or equivalent unit.

14.2.2 Visit 2

- Visit 2 comprises the clinical examination and a second non-contrast cranial CT or MRI scan which is performed to diagnose intracranial haemorrhage. Visit 2 will be done 48-96h after the administration of study medication. The following assessments will be performed:
- Physical examination
- Assessment of the neurological deficit by the NIHSS
- Measurement of blood pressure and heart rate
- Laboratory tests
- Non-contrast cranial CT or MRI scan
- Documentation of any concomitant medication
- Assessment of Adverse Event

14.3 Follow Up period (Visits V3, V4)

The Follow Up period comprises the period of the subacute stage (day 5-7 or hospital

discharge) in Visit 3， and the final follow up examination 90±7 days in Visit 4.

14.3.1 Visit 3

The following assessments will be performed:

- Physical examination
- Assessment of the neurological deficit by the NIHSS
- Measurement of blood pressure and heart rate
- Documentation of any concomitant medication
- Assessment of Adverse Event

14.3.3 Visit 4

The following assessments will be performed:

- Physical examination (If the patients return to local hospital)
- Assessment of the neurological deficit by the NIHSS
- Assessment of functional status by the mRS
- Assessment of functional health status and quality of life by the EQ-5D scale
- Documentation of any concomitant medication
- Assessment of Adverse Event

15. Endpoints of the Clinical Trial

15.1 Efficacy Analysis-Primary Endpoint

The primary outcome was the ordinal mRS score, a global measure of disability, comprises of seven grades ranging from 0 (no symptoms) to 6 (death).

15.2 Efficacy Analysis-Secondary Endpoints

- Proportion of patients with mRS score 0 to 4 at 90±7 days;
- Proportion of patients with mRS score 0 to 3 at 90±7 days;
- Proportion of patients with mRS score 0 to 2 at 90±7 days
- Proportion of patients with mRS score 0 to 1(or returned to pre-stroke modified Rankin scale score) at 90±7 days
- NIHSS score at 5-7 days after EVT or at early discharge;
- European Quality Five-Dimension scale score at 90 days.

15.3 Safety Analysis-Primary Endpoint

15.3.1 Primary Safety Endpoints

- Mortality due to any cause at 90 (±7) days;
- Proportion of patients with symptomatic intracranial haemorrhage within 48 hours after endovascular treatmen. Symptomatic intracranial haemorrhage will be adjudicated by an independent Imaging Core Laboratory and the Clinical Events Committee according to the modified Heidelberg Bleeding Classification, and is diagnosed based on a combination of: 1) clinical deterioration, 2) imaging findings, and 3) causal relatedness assessment. Clinical deterioration is defined as any of the following conditions: 1) NIHSS score increased more than 4 points than that

immediately before worsening; 2) NIHSS score increased more than 2 points in one category; 3) Deterioration led to intubation, hemicraniectomy, external ventricular drain placement or any other major interventions. The imaging criteria are presence of parenchymal hematoma type 2 (PH2), parenchymal hematoma type 1 (PH1), remote intracerebral haemorrhage (RIH), subarachnoid haemorrhage (SAH), intraventricular haemorrhage (IVH), or subdural haemorrhage. When clinical deterioration and PH2 co-occur, the event is automatically categorized as SICH. When clinical deterioration and PH1/RIH/SAH/IVH/SDH co-occur, the event is deemed SICH if it is judged that the haemorrhage contributed substantially to the clinical worsening. When clinical deterioration occurs only with haemorrhagic infarction type 1 or 2 (HI1 or HI2), the event is automatically classified as not SICH. ICH.

15.3.2 Secondary Safety Endpoints

- Proportion of patients with any radiologic intracranial haemorrhage within 48 hours after treatment;
- Proportion of patients with pneumonia;
- Proportion of patients with gastrointestinal haemorrhage within 7 days after EVT;
- Incidence of serious adverse events.
- Incidence of any adverse events.

16. Assessment

16.1 Assessment of efficacy

16.1.1 The modified Rankin Scale (mRS)

The mRS score is a valid and reliable clinician-reported measure of global disability that has been widely applied for evaluating recovery from stroke. It is a scale used to measure functional recovery (the degree of disability or dependence in daily activities) of people who have suffered a stroke[44,45]. mRS scores range from 0 to 6, with 0 indicating no residual symptoms; 5 indicating bedbound, requiring constant care; and 6 indicating death. The mRS score will be obtained at

Day 90. Premorbid mRS status will also be obtained retrospectively and reported on the 24 Hours CRF page. In this trial, we will keep video and voice recording versions of mRS score except those who die, unable, or refuse to take a video. For mRS score at Day 90, the mRS score will be assessed by two independent certified neurologists in a blinded manner with the video combined with voice record. For those who decline to participate in a video recording, the outcomes will be determined in person by site neurologists blinded to the treatment assignment, and keep a chat record with site neurologists. Disagreements are resolved by consensus.

The mRS will only be scored by those who have been trained and certified to use this scale using the table below. (See appendix 1)

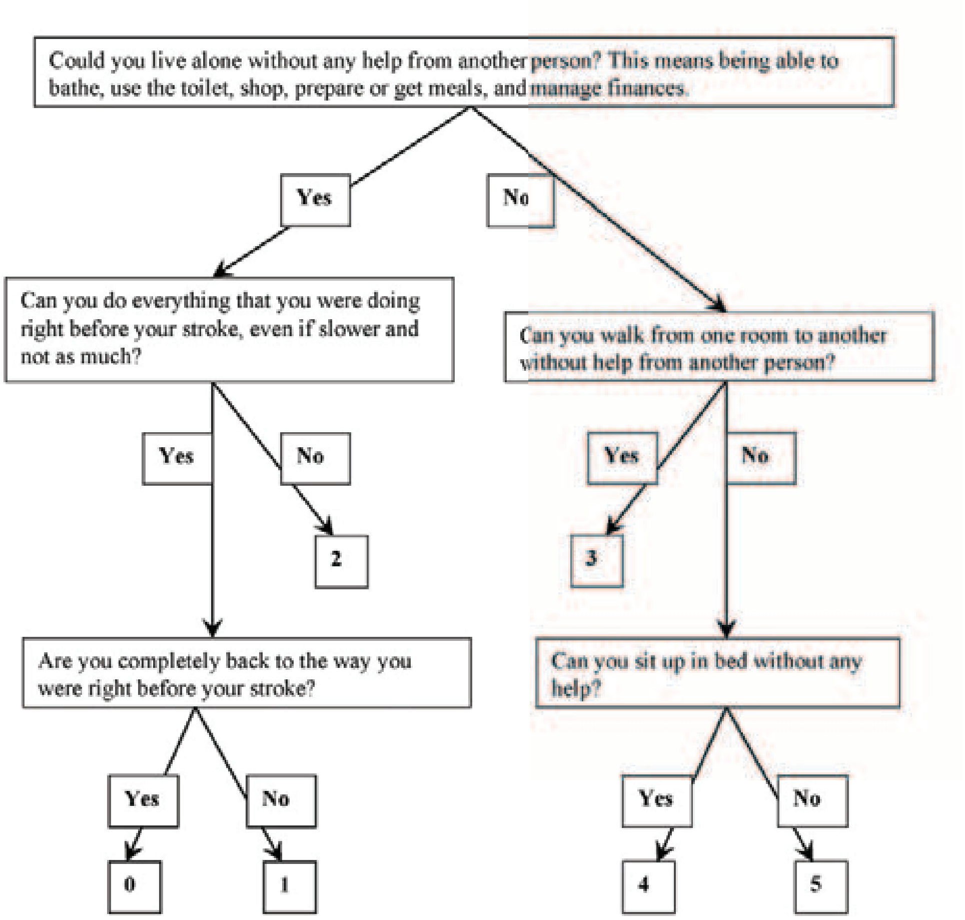

16.1.2 The National Institutes of Health Stroke Scale (NIHSS)

The NIHSS is a standardized neurological examination score that is a valid and reliable measure of disability and recovery after acute stroke[46]. Scores range from 0 to 42, with higher scores indicating more severe disability. The scale includes measures of level of consciousness, extra ocular movements, motor and sensory tests, coordination, language and speech evaluations. The NIHSS will be administered at baseline, Day 5-7 or discharge if earlier. The NIHSS will only be scored by those trained and certified in the use of this scale. In this trial, we will keep a video version of NIHSS score except in those who die or are unable or refuse to take a video. The

NIHSS score will be centrally assessed by two independent certified neurologists in a blinded manner via the video. Disagreements are resolved by consensus.(See appendix 2)

16.1.3 European Quality Five Dimensions Five Level scale

The EQ-5D-VAS is a generic instrument for describing and valuing health. The instrument is designed for self-completion, and respondents also rate their overall health on the day of the interview on a 0-100 hash-marked, vertical visual analogue scale. Patients who died will have a score of 0. The EQ-5D-VAS will be administered on Day 90±7 by those trained in the use of this scale. (See appendix 3)

16.2 Assessment of safety

16.2.1 Mortality at 90±7 days

Mortality at 90±7 days is one of the most important safety endpoints of the MARVEL trial. Mortality rates are defined as the number of deaths observed divided by the number of subjects observed over the 90-day study period.

16.2.2 Symptomatic Intracranial Haemorrhage

In addition to mortality, we will investigate SICH being the most feared complication for acute ischemic stroke. SICH within 48 hours will be adjudicated by an independent Imaging Core Laboratory and the Clinical Events Committee according to the Heidelberg Bleeding Classification.[47] SICH is diagnosed based on a combination of: 1) clinical deterioration, 2) imaging findings, and 3) causal

relatedness assessment. Clinical deterioration is defined as any of the following conditions: 1) NIHSS score increased more than 4 points than that immediately before worsening; 2) NIHSS score increased more than 2 points in one category; 3) Deterioration led to intubation, hemicraniectomy, external ventricular drain placement or any other major interventions. The imaging criteria are presence of parenchymal hematoma type 2 (PH2), parenchymal hematoma type 1 (PH1), remote intracerebral haemorrhage (RIH), subarachnoid haemorrhage (SAH), intraventricular haemorrhage (IVH), or subdural haemorrhage (SDH). When clinical deterioration and PH2 co-occur, the event is automatically categorized as SICH. When clinical deterioration and PH1/RIH/SAH/IVH/SDH co-occur, the event is deemed SICH if it is judged that the haemorrhage contributed substantially to the clinical worsening. When clinical deterioration occurs only with haemorrhagic infarction type 1 or 2 (HI1 or HI2), the event is automatically classified as not SICH.

16.2.3 Any ICH within 48 hours

The statistical analysis of differences in proportions of patients with any radiologic ICH within 48 hours between Methylprednisolone and Placebo groups will be performed.

The central reading will classify ICH according to the following intracerebral subtypes:

- Haemorrhagic infarct type 1 or type 2;
- Parenchymal hematoma type 1 or type 2;

- Remote intraparenchymal haemorrhage;
- In addition, intraventricular haemorrhage, subarachnoid haemorrhage, subdural haemorrhage, or epidural haemorrhage will be noted.

16.2.4 Proportion of patients with pneumonia

Pneumonia is considered when at least 2 signs (body temperature > 38°C; leukocytosis >12 000/mL, or leukopenia <4000/mL; purulent pulmonary secretions) associated with the appearance of a new infiltrate are present or when changes occur in an existing infiltrate on chest x-ray. If necessary, the diagnosis will be confirmed by a respiratory tract sample using a quantitative culture with a predefined positive threshold of 10^4 colony-forming units per milliliter (CFU/mL) for a bronchoalveolar lavage or non bronchoscopic sample, of 10^3 CFU/mL for a protected specimen brush and of 10^6 CFU/mL for a tracheal sample. The incidence of pneumonia will be reported by local investigators.

16.2.4 Proportion of patients with gastrointestinal bleeding.

The statistical analysis of differences in proportions of patients with gastrointestinal bleeding within 7 days between Methylprednisolone and Placebo groups will be performed. Gastrointestinal bleeding will be reported by local investigators.

16.2.5 Adverse events

The safety assessments will include monitoring and recording adverse events, including serious and non-serious adverse events, measuring vital signs specified in

the protocol, and other protocol specified tests that are considered to be critical to the safety evaluation of the study.

Any adverse change in health or the appearance of or worsening of any undesirable sign, symptom or medical condition occurring after enrolment into the trial will be recorded as Adverse Event whether or not it is considered to be related to the study drug. An adverse event also includes any occurrence that is a new illness; aggravated in severity of frequency from the baseline condition, abnormal results of diagnostic procedures, or a combination of the above. The assessment of severity and relationship of adverse events are determined by using the following definitions.

Table 3: Severity categorization and relationship definitions of Adverse Event.

Severity categorization		
Grade1	Mild	It is usually transient and may require only minimal treatment or therapeutic intervention. The event doesn't generally interfere with usual activities of daily living.
Grade2	Moderate	It is usually alleviated with additional specific therapeutic intervention. The event interferes with usual activities of daily living, causing discomfort but poses no significant or permanent risk of harm to the research subject.
Grade3	Severe	It interrupts usual activities of daily living, or significantly affects clinical status, or may require intensive therapeutic intervention.

Grade4	Life-threatening	Substantial risk of dying at time of event
Grade5	Death	Death
Relationship of adverse events		
Related	A clinical event, including laboratory test abnormality, where there is a "reasonable possibility" that the serious adverse event was caused by the study medication, meaning that there is evidence or arguments to suggest a causal relationship.	
Probably	A clinical event, including laboratory test abnormality, with a reasonable time sequence to drug administration, unlikely to be attributed to concurrent disease or other drugs or chemicals, and which follows a clinically reasonable response on withdrawal.	
Possibly	A clinical event, including laboratory test abnormality, with a reasonable time sequence to drug administration, but which could also be explained by concurrent disease or other drugs or chemicals. Information on drug withdrawal may be lacking or unclear.	
Unrelated	This category is applicable to adverse events which are judged to be clearly and incontrovertibly due to extraneous causes (diseases, environment, etc.) and do not meet the criteria for drug relationship listed for the above-mentioned conditions.	

16.2.6 Serious Adverse Event

A Serious Adverse Event is any untoward medical occurrence (whether deemed to be related to investigational medication or not) that meets any of the following criteria:

- Fatal (i.e., the adverse event actually causes or leads to death)
- Life-threatening (i.e., the adverse event, in the view of the investigator, places the patient at immediate risk of death). This does not include any adverse event that, had it occurred in a more severe form or was allowed to continue, might have caused death.
- Requires or prolongs inpatient hospitalization
- Results in persistent or significant disability/incapacity (i.e., the adverse event results in substantial disruption of the patient's ability to conduct normal life functions)
- Congenital anomaly/birth defect in a neonate/infant born to a mother exposed to study drug
- Significant medical event in the investigator's judgment (e.g., may jeopardize the patient or may require medical/surgical intervention to prevent one of the outcomes listed above)

16.2.7 Adverse Event Recording and Follow Up

The investigator is responsible for evaluating and reporting any adverse events occurring during the study. The investigator will record onset, duration, intensity, any taken action, evolution/outcome, and the causality assessment for any adverse event.

Any adverse events leading to withdrawal from the study and those that persist at the end of the study must be followed up.

16.2.8 Serious Adverse Events Reporting

Although adverse events are recorded in the adverse event form of CRF special reporting requirements apply to the reporting of serious adverse events. Serious adverse events need to be reported immediately. The investigator should immediately notify the principal investigator of any serious adverse events that occurred at the trial site within 24 hours: MARVEL security desk Fax: +86 023-68774270

Serious adverse event reports will be collected centrally at the safety desk. Copies of all serious adverse event reports will be sent to the central trial management. Such preliminary reports will be followed by detailed descriptions, including anonymous copies of hospital case reports or related results, as well as other documents (if required and applicable). All serious adverse events that have not been resolved by the end of the study, or that have not been resolved upon discontinuation of the subject's participation in the study, must be followed until any of the following occurs:

- the event resolves
- the event stabilizes
- the event returns to baseline, if a baseline value is available
- the event can be attributed to agents other than the study drug or to factors unrelated to study conduct

- when it becomes unlikely that any additional information can be obtained

Any additional information known after the event has been initially reported should be sent using a new serious adverse event report form. New information will be noted on the "serious adverse event" form, by ticking the box marked "follow-up" and sending to MARVEL SAFETY DESK.

Patients who withdraw from the study treatment due to any adverse event will be followed up at least until the outcomes are determined, even if it implies that the follow-up continues after the patient has left the trial.

The investigator should assess the seriousness of the adverse events. This is based on the regulatory definitions of seriousness. The investigator should assess the causal relationship of serious adverse events. This is a clinical assessment of whether the adverse events may be related to the investigational medication. The evaluation of the expectedness is based on knowledge of adverse reactions and references.

17. Study Discontinuation Criteria

17.1 Patient Discontinuation

Patients have the right to voluntarily withdraw from the study at any time for any reason. In addition, the investigator has the right to withdraw the patient from the study at any time. Reasons for withdrawal from the study may include, but are not limited to the following:

- Patient withdraws consent at any time

- Any medical condition determined by the investigator may endanger the patient's safety if he or she continues the study
- The investigator determines that it is in the best interests of the patient
- Patient noncompliance, specifically defined as unwillingness to participate in a 3-month assessment of neurological status and function

Every effort should be made to obtain information about patients who withdrew from the study. The main reasons for withdrawal from the study should be recorded on the corresponding CRF. Patients who withdraw from the study will not be replaced.

17.2 Study Treatment Discontinuation

The treatment with the investigate medication should be terminated immediately if the patients experience any of the following:

- Any serious gastrointestinal bleeding.
- Uncontrollable hyperglycaemia (blood sugar >22.2 mmol/L)
- Anaphylactic reaction
- Continuation of the study drug would be detrimental to the patient’s well-being.
- Withdrawal for personal reasons.

According to the intention to treat (ITT) principle, patients who discontinue study treatment prematurely will continue in the study and be followed up to the 90th day.

17.3 Study and Site Discontinuation

The investigators has the right to terminate this study in any case of concern about the safety of patients caused by new information. The reasons for terminating the study may include, but are not limited to:

- The incidence or severity of adverse events in this or other studies indicates a potential health hazard to patients.
- Patient enrolment is unsatisfactory

The investigators have the right to close a site at any time. Reasons for closing a site may include, but are not limited to, the following:

- Excessively slow recruitment
- Poor protocol adherence
- Inaccurate or incomplete data recording
- Non-compliance with the International Conference on Harmonization guideline for Good Clinical Practice
- No study activity

18. Statistical Analysis

This study will test whether treatment of patients with acute ischemic stroke with intravenous methylprednisolone will lead to a lower mRS score at 90 days.

18.1 Determination of sample size

The current trial is designed detect a shift on the modified Rankin Scale score, which represents the global disability, to a lower score. The expected distribution of the modified Rankin Scale score, derived from the EVT for acute anterior circulation ischemic stroke (ACTUAL) registry[40] and the Highly Effective Reperfusion evaluated in Multiple Endovascular Stroke Trials (HERMES) collaboration[7], is as follows: 7%, 20%, 16%, 11%, 13%, 8% and 25%.

The sample size was calculated using the cumulative proportion of patients with mRS 0-2. We assume a moderate effect of 7% absolute increase in the cumulative proportion of patients with mRS 0-2 in the intervention group, compared with controls, indicating an odds ratio (OR) of 1.33, which have substantially exceeds the minimal clinically important difference.[41,42] In order to demonstrate the expected treatment effect with a type-1 error alpha =0.05 (two-tailed) and a power of 80% (beta = 20%). A sample size of n= 1588 patients (n=794 per treatment group) is required.

The intention-to-treat principle will be applied to the primary analysis and, therefore, to safeguard against dilution of the treatment effect associated with an approximate 5% non-adherence rate (due to loss to follow-up, consent withdrawal and other reasons), we initially planned to enrol n=1672 patients (n=836 per treatment group) for this study. This estimation was performed based on PASS (NCSS, LLC. Kaysville, Utah, USA) version 15.0.

18.2 Analysis population

18.2.1 Intention-to-Treat Population

The ITT population includes all patients randomized into the trial who were recorded as receiving any amount of study drug, even if the subject does not receive the correct treatment, or does not follow the protocol until completion[48]. The ITT population will be the primary analysis population for the efficacy endpoints and subjects will be analysed according to the treatment group to which they were assigned at randomization.

18.2.2. Per-Protocol Population

The PP population is defined as the subset of the ITT population excluding major protocol violators deemed to have the potential to affect patient outcome in terms of efficacy.

The PP population includes patients who actually received the assigned treatment and do not have major protocol violations or deviations. Major protocol violations or deviations will be identified in a blinded fashion prior to database lock. More specifically, patients with anyone of the following criteria will be excluded from the PP population. These deviations will be determined based on the medical monitors' records, as well as programmatically, using the following criteria at a minimum:

- Received but did not complete treatment with study drug, or dose of study drug administered outside recommended dose.
- Received study drug after 2 hours after arterial access closure.
- Violated inclusion or exclusion criteria.

A list of patients to be excluded from the randomized patients to create the PP-Efficacy analysis will be established and validated by the Steering Committee prior to unblinding.

18.2.3. Safety Population

The Safety Population includes all patients who received any amount of study drug. In case of violation of the randomization scheme, patients will be classified according to the treatment they actually received. Patients will be assigned to the different populations prior to unblinding of the database. Patients who withdraw informed consent immediately after randomization and are not to receive any treatment should be excluded from Safety Population.

18.2 Analysis of Efficacy–primary endpoint

The primary efficacy analysis will estimate the effect of treatment with intravenous methylprednisolone and with the placebo in acute ischemic stroke patients.

The primary efficacy outcome is the global disability defined by mRS score at 90 days post-randomization. Adjusted common odds ratio or assumption free method will be calculated. The adjusted covariates include age, baseline NIHSS score, pre-stroke mRS, baseline ASPECTS score, use of intravenous thrombolysis, time from onset to randomization, and occlusion location. (Details are described in SAP Section 12.1). We will analyse the primary outcome in the intention-to-treat (ITT) and per-

protocol (PP) population.

18.3 Analysis of Efficacy – secondary endpoints

The mRS score will be used to evaluate the proportion of patients with a score = 0 to 4 *versus* 5 or 6, 0 to 3 *versus* 4 or higher, 0 to 2 *versus* 3 or higher, 0 to 1(or return to pre stroke morbidity) *versus* 2 or higher and at day 90±7. The adjusted risk ratio will be calculated by fitting the GLM models separately.

NIHSS score at 5-7 days or at early discharge and Functional health status quality of life 90 (±7) days after randomization (EQ-5D-VAS). The EQ-5D-VAS will be compared between the methylprednisolone group and the placebo group via GLM.

Normality and variance homogeneity of residuals will be accessed graphically. The win ratio method will be used if normality assumption is seriously violated.

The significance of each test is determined at the two-sided alpha level of 0.05. The specific statistical model for analysing each of these outcome measures will be detailed in the Statistical Analysis Plan (SAP).

18.4 Safety analysis

Safety outcomes include incidence of overall mortality at 90 (±7) days after randomization, the proportion of patient with SICH within 48 hours after treatment, the proportion of patient with any ICH within 48 hours after treatment, proportion of patients with pneumonia and proportion of patients with gastrointestinal haemorrhage within 7 days after EVT;. The safety analysis will be performed on the Safety

Population. The incidences of each outcome will be compared via Chi-square test or Fisher's exact test. The modified Poisson regression models will be fitted to estimate the risk-ratio associated with the treatment effect. Risk ratio with 95% CI will be reported. Patients with missing outcome will not be included in the regression analysis. Additionally, Log-Rank test and the Kaplan-Meier estimates will be plotted over the observation period of 90 days for mortality. Cox regression model will be employed to calculate the hazard ratio with 95%CI.

18.5 Tolerability Analysis (adverse events)

Tolerability analyses will be performed on the Safety Population only.

18.5.1 Adverse Events

Adverse Events will be coded using the Medical Dictionary for Regulatory Activities (MedDRA) central coding dictionary.

18.5.2 Serious Adverse Events

Serious Adverse Event reconciliation will be performed by Data Management, Clinical Research, and Pharmacovigilance via data listings.

A summary of patients with serious adverse events will be presented by treatment group for all patients in the Safety Population. A data listing of SAEs will also be provided, displaying details of the event(s) captured on the CRF. The between group difference will be tested using Chi-square test or Fisher's exact test.

18.5.3 All Adverse Events

Summaries of patients with AEs by System Organ Class and Preferred Term (MedDRA) will be prepared. Each patient will be counted only once within each category (System Organ Class or Preferred Term). If a patient experiences more than one AE within a category, only the AE with the strongest relationship or the greatest intensity, as appropriate, will be included in the summaries. The between group difference will be tested using Chi-square test or Fisher's exact test. Only AEs beginning at or after the beginning of study drug administration will be included.

18.6 Adjustment for Covariates and Subgroup Analyses

In addition to the primary and secondary analyses adjusting for age, baseline NIHSS score, pre-stroke mRS, baseline ASPECTS score, the use of intravenous thrombolysis, time from onset to randomization, and occlusion location, exploratory analyses will be conducted to determine the potential roles of common baseline characteristics and assess potential heterogeneity of treatment effect across subgroups. The primary pre-specified subgroups as follows will be explored.

- age
- sex
- baseline NIHSS score
- pre-stroke mRS
- baseline ASPECTS score
- intravenous thrombolysis

- time from last known well to randomization. (mins)
- occlusion location
- Complete Reperfusion defined as Extended Thrombolysis in Cerebral Infarction grade 2c or 3
- Patients with any radiological haemorrhages
- Patients with symptomatic haemorrhages

18.7 Missing data handling

All efforts will be made to minimize the amount of missing data. However, some missing data may be inevitable due to, for example, loss to follow-up. Missing baseline covariates will be imputed using simple imputation or multiple imputation methods (Details in SAP Section 9.2.2). Sensitivity analyses based on different hypotheses about the missingness pattern of the primary outcome will be performed to test for the robustness of the primary analysis (Details in SAP Section 12.1.5).

19. Ethical and regulatory consideration

19.1 General requirements and Considerations

This study followed the ethical principles of the Helsinki Declaration. Approval of the conduct of the trial will be obtained from the Ethics Committees of all participating centres as well as from the local regulatory authorities. The trial will not start in any centre before written approval and authorization by the respective Ethics Committee and Regulatory Authority. Any subsequent protocol amendment will be submitted to

the Ethics Committee for approval. The involvement of committees in the clinical trial of MARVEL will further ensure that the subjects have the highest priority at any time. In addition to the treatment to be tested, all diagnostic procedures and treatments applied are part of standard management of acute stroke patients and will follow the national guidelines. These procedures are therefore of immediate benefit to the patients. The investigators will assure that every patient participating in the trial will receive best medical treatment.

19.2 Study monitoring and quality control

The design of the MARVEL trial has been carefully reviewed and approved by the Steering Committee before being submitted to the Ethics Committees and Regulatory Authorities for approval. In addition, the independent EAB and DSMB and an external SAB have reviewed and approved the trial protocol and will continuously monitor the conduction of the trial. These committees, composed of well-known independent experts, will ensure sufficient alertness to all ethical and safety issues.

The investigators promise to conduct the MARVEL trial in accordance with this trial protocol, International Conference on Harmonization-GCP (ICH-GCP) Guidelines and applicable regulatory requirements. The investigators agree to provide reliable data and all information required by the trial protocol in an accurate and legible manner according to the instructions provided.

The investigators of this clinical trial is responsible to the health authorities and takes all reasonable measures to ensure the correct implementation of the clinical trial

protocol in terms of ethics, clinical trial protocol compliance, and the completeness and validity of the data recorded on the CRF.

The main responsibility of the monitoring team is to help investigators to ensure that all aspects of clinical trials are highly ethical, scientific, professional and standardized. The monitoring team will regularly contact the centres through field visits, emails or phone calls, and send inspectors to assess the progress of the trial, the compliance of investigators and patients with the trial protocol, and to resolve urgent issues. During these inspection visits, the inspector will work with the on-site investigator. The main aspects of inspection and monitoring are as follows (not exclusive): patient informed consent, patient recruitment and follow-up, serious adverse event recording and reporting, study drug supply, treatment compliance of study drug group participants, study drug count, concomitant treatment and data quality.

19.3 Informed Consent

The written informed consent must be obtained from all participants in the clinical trial prior to inclusion into the study. Informed consent forms must be written to be easily understood by the participants or their legal representatives, enabling them to understand the purpose of the trial, procedures, possible benefits, potential risks, and the rights/obligations of participation. Participants have the right to withdraw from the study at any stage of the trial. Each participant must leave contact information to the investigator of the coordinating centre. At the same time, the investigator must leave

his own phone number to the participant so that the participant can find the investigator at any time.

19.4 Confidentiality

Personal data will be processed in accordance with Chinese data protection directives and regulations, relevant international legislation and good practices. Data will only be processed for the trial's purposes. The investigators encode each patient participating in the study by assigning a unique patient identification number to maintain confidentiality standards. This means that all individual patients' data will be linked to the CRF via a unique identification number throughout the trial. Individual patient's medical information will be recorded only in anonymous form. The clinical monitors may inspect source data in order to ensure the accuracy of the data recorded in the CRF.

19.5 Liability and Insurance

The study investigators provide an appropriate insurance for patients in the event of any trial related damage in accordance with applicable national laws. A certificate of insurance will be provided to the investigator of the coordinating centre in which this document is required.

20. Administrative procedures

20.1 Curriculum vitae

A latest copy of the curriculum vitae of each investigator and co-investigator will be provided to the responsible coordinating centre prior to the start of the study.

20.2 Secrecy agreement

The investigators will take all necessary measures to ensure that there is no violation of confidentiality in respect of all information accumulated, acquired or deduced in the course of the trial, other than that information to be disclosed by law.

20.3 Ownership of data and use of the study results

All materials, information and unpublished documentation supplied to the investigators, inclusive of this study, and the patient case report forms are the exclusive property of the study initiators. Therefore, the study initiators reserve the right to use the data of the present study, either in the form of case report forms, or in the form of a report, with or without comments and with or without analysis, in order to submit them to the health authorities.

20.4 Protocol amendments

Any protocol amendments will be prepared by the investigators. Protocol amendments should be submitted to the REB/IRB for approval prior to implementation in accordance with local regulatory requirements. Approval must be obtained from the REB/IRB and regulatory authorities (as locally required) before implementation of any changes, except for changes necessary to eliminate an immediate hazard to patients or changes that involve logistical or administrative aspects only

21. Data Retention

The double reviewed case report form (CRF) and imaging data will be sent to the data management group. The person in charge of the data management group will check and sign the receipt form. The CRF will be kept by the research centre after data entry is completed.

22. Study report and publications

The results of the trial will be reported to the regulatory authorities and ethics committees. The investigators will provide an annual safety report and the final report.

According to the pre-defined analysis in the clinical trial protocol, the results of the trial will be published in the appropriate journal (for manuscripts) or meeting (for abstracts).

By signing the clinical trial protocol， the investigator agrees that the results of the clinical trial can be used for publication.

The trial will be registered at the Chinese Clinical Trial Registry website.

23.?References?Uncategorized References

1. Global, regional, and national burden of stroke and its risk factors, 1990-2019: a systematic analysis for the Global Burden of Disease Study 2019. Lancet Neurol 2021;20:795-820.
2. Diseases GBD, Injuries C. Global burden of 369 diseases and injuries in 204 countries and territories, 1990-2019: a systematic analysis for the Global Burden of Disease Study 2019. Lancet 2020;396:1204-22.
3. Lindsay MP, Norrving B, Sacco RL, et al. World Stroke Organization (WSO): Global Stroke Fact Sheet 2019. Int J Stroke 2019;14:806-17.
4. Wang W, Jiang B, Sun H, et al. Prevalence, Incidence, and Mortality of Stroke in China: Results from a Nationwide Population-Based Survey of 480 687 Adults. Circulation 2017;135:759-71.
5. Baron JC. Protecting the ischaemic penumbra as an adjunct to thrombectomy for acute stroke. Nat Rev Neurol 2018;14:325-37.
6. Powers WJ, Rabinstein AA, Ackerson T, et al. Guidelines for the Early Management of Patients With Acute Ischemic Stroke: 2019 Update to the 2018 Guidelines for the Early Management of Acute Ischemic Stroke: A Guideline for Healthcare Professionals From the American Heart Association/American Stroke Association. Stroke 2019;50:e344-e418.
7. Goyal M, Menon BK, van Zwam WH, et al. Endovascular thrombectomy after large-vessel ischaemic stroke: a meta-analysis of individual patient data from five randomised trials. LANCET 2016;387:1723-31.
8. Nie X, Pu Y, Zhang Z, Liu X, Duan W, Liu L. Futile Recanalization after Endovascular Therapy in Acute Ischemic Stroke. Biomed Res Int 2018;2018:5879548.
9. Stoll G, Pham M. Beyond recanalization — a call for action in acute stroke. Nature Reviews Neurology 2020;16:591-2.
10. Whiteley WN, Emberson J, Lees KR, et al. Risk of intracerebral haemorrhage with alteplase after acute ischaemic stroke: a secondary analysis of an individual patient data meta-analysis. Lancet Neurol 2016;15:925-33.
11. Shi ZS, Duckwiler GR, Jahan R, et al. Early Blood-Brain Barrier Disruption after Mechanical Thrombectomy in Acute Ischemic Stroke. J Neuroimaging 2018;28:283-8.
12. Conti E, Piccardi B, Sodero A, et al. Translational Stroke Research Review: Using the Mouse to Model Human Futile Recanalization and Reperfusion Injury in Ischemic Brain Tissue. Cells 2021;10:19.
13. Cheripelli BK, Huang XY, MacIsaac R, Muir KW. Interaction of Recanalization, Intracerebral Hemorrhage, and Cerebral Edema After Intravenous Thrombolysis. Stroke 2016;47:1761-7.
14. Chen XL, Huang Q, Deng QW, et al. A prediction model of brain edema after endovascular treatment in patients with acute ischemic stroke. Journal of the Neurological Sciences 2019;407.
15. Ames A, 3rd, Wright RL, Kowada M, Thurston JM, Majno G. Cerebral ischemia. II. The no-reflow phenomenon. The American journal of pathology 1968;52:437-53.
16. Caiazzo G, Musci RL, Frediani L, et al. State of the Art: No-Reflow Phenomenon. Cardiol Clin 2020;38:563-73.
17. Kloner RA, King KS, Harrington MG. No-reflow phenomenon in the heart and brain. Am J Physiol Heart Circ Physiol 2018;315:H550-H62.
18. Ng FC, Churilov L, Yassi N, et al. Microvascular Dysfunction in Blood-Brain Barrier Disruption and Hypoperfusion Within the Infarct Posttreatment Are Associated With Cerebral Edema. 2022;53:1597-605.
19. Planas AM. Role of Immune Cells Migrating to the Ischemic Brain. Stroke 2018;49:2261-7.
20. Shi KB, Tian DC, Li ZLG, Ducruet AF, Lawton MT, Shi FD. Global brain inflammation in stroke. Lancet Neurology 2019;18:1058-66.
21. Laha RK, Dujovny M, Barrionuevo PJ, DeCastro SC, Hellstrom HR, Maroon JC. Protective effects of methyl prednisolone and dimethyl sulfoxide in experimental middle cerebral artery embolectomy. J Neurosurg

1978;49:508-16.
22. Slivka AP, Murphy EJ. High-dose methylprednisolone treatment in experimental focal cerebral ischemia. Exp Neurol 2001;167:166-72.
23. de Courten-Myers GM, Kleinholz M, Wagner KR, Xi G, Myers RE. Efficacious experimental stroke treatment with high-dose methylprednisolone. Stroke 1994;25:487-92; discussion 93.
24. Barbosa-Coutinho LM, Hartmann A, Hossmann KA, Rommel T. Effect of dexamethasone on serum protein extravasation in experimental brain infarcts of monkey: an immunohistochemical study. Acta Neuropathol 1985;65:255-60.
25. Espinosa A, Meneses G, Chavarría A, et al. Intranasal Dexamethasone Reduces Mortality and Brain Damage in a Mouse Experimental Ischemic Stroke Model. Neurotherapeutics 2020;17:1907-18.
26. Altamentova S, Rumajogee P, Hong J, et al. Methylprednisolone Reduces Persistent Post-ischemic Inflammation in a Rat Hypoxia-Ischemia Model of Perinatal Stroke. Transl Stroke Res 2020;11:1117-36.
27. Limbourg FP, Huang Z, Plumier JC, et al. Rapid nontranscriptional activation of endothelial nitric oxide synthase mediates increased cerebral blood flow and stroke protection by corticosteroids. J Clin Invest 2002;110:1729-38.
28. Norris JW. Steroids may have a role in stroke therapy. Stroke 2004;35:228-9.
29. Nellis SH, Roberts BH, Kinney EL, Field J, Ummat A, Zelis R. Beneficial effect of dexamethasone on the "no reflow" phenomenon in canine myocardium. Cardiovasc Res 1980;14:137-41.
30. Sandercock PAG, Soane T. Corticosteroids for acute ischaemic stroke. Cochrane Database of Systematic Reviews 2011.
31. Ogun SA, Odusote KA. Effectiveness of high dose dexamethasone in the treatment of acute stroke. West African journal of medicine 2001;20:1-6.
32. Nie X, Leng X, Miao Z, Fisher M, Liu L. Clinically Ineffective Reperfusion After Endovascular Therapy in Acute Ischemic Stroke. 2023;54:873-81.
33. Lyden P, Buchan A, Boltze J, et al. Top Priorities for Cerebroprotective Studies—A Paradigm Shift: Report From STAIR XI. Stroke 2021;52:3063-71.
34. Lapchak PA. Emerging Therapies: Pleiotropic Multi-target Drugs to Treat Stroke Victims. Translational Stroke Research 2011;2:129-35.
35. Fraser JF, Pahwa S, Maniskas M, et al. Now that the door is open: an update on ischemic stroke pharmacotherapeutics for the neurointerventionalist. Journal of Neurointerventional Surgery 2023.
36. Savitz SI, Baron JC, Fisher M, Consortium SX. Stroke Treatment Academic Industry Roundtable X: Brain Cytoprotection Therapies in the Reperfusion Era. Stroke 2019;50:1026-31.
37. Savitz SI, Baron J-C, Yenari MA, Sanossian N, Fisher M. Reconsidering Neuroprotection in the Reperfusion Era. 2017;48:3413-9.
38. Davis SM, Donnan GA. Steroids for stroke: another potential therapy discarded prematurely? Stroke 2004;35:230-1.
39. Huting ZHANG SL. Influence of neurological intervention combined with dexamethasone injection on the outcome for the patients suffered from acute ischemic stroke. Journal of Molecular Imaging 2016;39:121-4.
40. Zi W, Wang H, Yang D, et al. Clinical Effectiveness and Safety Outcomes of Endovascular Treatment for Acute Anterior Circulation Ischemic Stroke in China. Cerebrovasc Dis 2017;44:248-58.
41. Cranston JS, Kaplan BD, Saver JL. Minimal Clinically Important Difference for Safe and Simple Novel Acute Ischemic Stroke Therapies. Stroke 2017;48:2946-51.
42. Berkhemer OA, Fransen PSS, Beumer D, et al. A Randomized Trial of Intraarterial Treatment for Acute Ischemic Stroke. New England Journal of Medicine 2015;372:11-20.

43. Wang Y, Han S, Qin H, et al. Chinese Stroke Association guidelines for clinical management of cerebrovascular disorders: executive summary and 2019 update of the management of high-risk population. Stroke Vasc Neurol 2020;5:270-8.
44. Banks JL, Marotta CA. Outcomes validity and reliability of the modified Rankin scale: implications for stroke clinical trials: a literature review and synthesis. Stroke 2007;38:1091-6.
45. Quinn TJ, Dawson J, Walters MR, Lees KR. Reliability of the modified Rankin Scale: a systematic review. Stroke 2009;40:3393-5.
46. Brott T, Adams HP, Jr., Olinger CP, et al. Measurements of acute cerebral infarction: a clinical examination scale. Stroke 1989;20:864-70.
47. von Kummer R, Broderick JP, Campbell BC, et al. The Heidelberg Bleeding Classification: Classification of Bleeding Events After Ischemic Stroke and Reperfusion Therapy. Stroke 2015;46:2981-6.
48. White IR, Horton NJ, Carpenter J, Pocock SJ. Strategy for intention to treat analysis in randomised trials with missing outcome data. Bmj 2011;342

24. Appendices

24.1 Appendix 1: mRS

Subject ID ___ ___ ___ ___ ___ ___ ___ ___ ___ ___
Subject Date of Birth ___ ___/___ ___/___ ___
Hospital ID ______________________________
Date of Examination ___ ___/___ ___/___ ___

The Modified Rankin Scale (mRS)

(Use web calculator at www.modifiedrankin.com)

0 No symptoms

1 No significant disability; able to carry out all usual activities, despite some symptoms

2 Slight disability; able to look after own affairs without assistance, but unable to carry out all previous activities

3 Moderate disability; requires some help, but able to walk unassisted

4 Moderately severe disability; unable to attend to own bodily needs without assistance, and unable to walk unassisted

5 Severe disability; requires constant nursing care and attention, bedridden, incontinent

6 Dead

References:

Rankin J (May 1957). "Cerebral vascular accidents in patients over the age of 60. II. Prognosis". Scott Med J 2 (5): 200–15

Patel, N., et al. Simple and reliable determination of the modified Rankin Scale in neurosurgical and neurological patients: The mRS-9Q. *Neurosurgery*, published online in advance of print 26 July 2012

24.2 Appendix 2: NIHSS

NIHSS page 1

Subject ID ___ ___ ___ ___ ___ ___ ___ ___ ___ ___
Subject Date of Birth ___ ___/___ ___/___ ___
Hospital ID ____________________
Date of Examination ___ ___/___ ___/___ ___

NATIONAL INSTITUTES OF HEALTH STROKE SCALE (NIHSS)[1]

Interval: [ ] Baseline
[ ] 7-10 days
[ ] 1 month
[ ] 3 months
[ ] 6 months
[ ] Other ____________________

Time: ___ ___:___ ___ [ ]am [ ]pm

Person Administering Scale ____________________

Purpose:
The NIH Stroke Scale (NIHSS) is a standardized neurological examination intended to describe the neurological deficits found in large groups of stroke patients participating in treatment trials.

Administer stroke scale items in the order listed. Record performance in each category after each subscale exam. Do ***not*** go back and change scores. Follow directions provided for each exam technique. Scores should reflect what the patient does, not what the clinician thinks the patient can do. The clinician should record answers while administering the exam and work quickly. Except where indicated, the patient should not be coached (i.e., repeated requests to patient to make a special effort).

Instructions	Scale Definition	Score
**1a. Level of Consciousness:** The investigator must choose a response if a full evaluation is prevented by such obstacles as an endotracheal tube language barrier, orotracheal trauma/bandages. A 3 is scored only if the patient makes no movement (other than reflexive posturing) in response to noxious stimulation.	0 = **Alert**; Keenly responsive.<br>1 = **Not alert, but arousable** by minor stimulation to obey, answer or respond.<br>2 = **Not alert**; requires repeated stimulation to attend, or is obtunded and requires strong or painful stimulation to make movements (not stereotyped).<br>3 = **Responds only with reflex motor or autonomic effects or totally unresponsive**, flaccid, and areflexic.	______
**1b. LOC Questions:** The patient is asked the month and his/her age. The answer must be correct – there is no partial credit for being close. Aphasic and stuporous patients who do not comprehend the questions will score 2. Patients unable to speak because of endotracheal intubation, orotracheal trauma, severe dysarthria from any cause, language barrier, or any other problem not secondary to aphasia are given a 1. It is important that only the initial answer be graded and that the examiner not "help" the patient with verbal or non-verbal cues.	0 = **Answers both questions correctly.**<br>1 = **Answers one question correctly.**<br>2 = **Answers neither question correctly.**	______

[1] The National Institute of Neurological Diseases and Stroke (NINDS), National Institutes of Health (NIH), Last Revised 01 October 2003 (https://stroke.nih.gov/resources/index.htm).

NIHSS page 2

Instructions	Scale Definition	Score
**6. Motor Leg:** The limb is placed in the appropriate position: hold the leg at 30 degrees (always tested supine). Drift is scored if the leg falls before 5 seconds. The aphasic patient is encouraged using urgency in the voice and pantomime, but not noxious stimulation. Each limb is tested in turn, beginning with the non-paretic leg. Only the case of amputation or joint fusion at the hip, should the examiner record the score as untestable (UN), and clearly write the explanation for this choice.	0 = **No drift**; limb holds 90 (or 45) degrees for full 10 seconds.<br>1 = **Drift**; limb holds 90 (or 45) degrees, but drifts down before full 10 seconds; does not hit bed or other support.<br>2 = **Some effort against gravity**; limb cannot get to or maintain (if cued) 90 (or 45) degrees, drifts down to bed, but has some effort against gravity.<br>3 = **No effort against gravity**; leg falls to bed immediately.<br>4 = **No movement**.<br>UN= **Amputation** or joint fusion; explain: ____________<br>6a= **Left Arm**.<br>6b= **Right Arm**.	________<br>________
**7. Limb Ataxia:** This item is aimed at finding evidence of a unilateral cerebellar lesion. Test with eyes open. In case of visual defect, ensure testing is done in intact visual field. The finger-nose-finger and heel-shin tests are performed on both sides, and ataxia is scored only if present out of proportion to weakness. Ataxia is absent in the patient who cannot understand or is paralyzed. Only in the case of amputation or joint fusion, should the examiner record the score as untestable (UN), and clearly write the explanation for this choice. In case of blindness, test by having the patient touch nose from extended arm position.	0 = **Absent.**<br>1 = **Present in one limb**.<br>2 = **Present in two limbs**.<br>UN= **Amputation** or joint fusion; explain: ____________	________
**8. Sensory:** Sensation or grimace to pinprick when tested, or withdrawal from noxious stimulus in the obtunded or aphasic patient. Only sensory loss attributed to stroke is scored as abnormal and the examiner should test as many body areas [arms (not hands), legs, trunk, face) as needed to accurately check for hemisensory loss. A score of 2, "severe or total sensory loss," should only be given when a severe or total loss of sensation can be clearly demonstrated. Stuporous and aphasic patients will, therefore, probably score 1 or 0. The patient with brainstem stroke who has bilateral loss of sensation is scored 2. If the patient does not respond and is quadriplegic, score 2. Patients in a coma (item 1a=3) are automatically given a 2 on this item.	0 = **Normal**; no sensory loss.<br>1 = **Mild-to-moderate** sensory loss; patient feels pinprick is less sharp or is dull on the affected side, or there is a loss of superficial pain with pinprick, but patient is aware of being touched.<br>2 = **Severe to total** sensory loss; patient is not aware of being touched in the face, arm, and leg.	________
**9. Best Language:** A great deal of information about comprehension will be obtained during the preceding sections of the examination. For this scale item, the patient is asked to describe what is happening in the attached picture, to name the items on the attached naming sheet and to read from the attached list of sentences. Comprehension is judged from responses here, as well as to all of the commands in the preceding general neurological exam. If visual loss interferes with the tests, ask the patient to identify objects placed in the hand, repeat, and produce speech. The intubated patient should be asked to write. The patient in a coma (item 1a=3) will automatically score 3 on this item. The examiner must choose a score for the patient with stupor or limited cooperation, but a score of 3 should be used only if the patient is mute and follows no one-step commands.	0 = **No asphasia**; normal.<br>1 = **Mild-to-moderate aphasia**; some obvious loss of fluency or facility of comprehension, without significant limitation on ideas expressed or form of expression. Reduction of speech and/or comprehension, however, makes conversation about provided materials difficult or impossible. For example, in conversation about provided materials, examiner can identify picture or naming card content from patient's response.<br>2 = **Severe asphasia**; all communication is through fragmentary expression; great need for inference, questioning, and guessing by the listener. Range of information that can be exchanged is limited; listener carries burden of communication. Examiner cannot identify materials provided from patient response.<br>3 = **Mute, global aphasia**; no usable speech or auditory comprehension.	________

NIHSS page 3

Instructions	Scale Definition	Score
**1c. LOC Commands:** The patient is asked to open and close the eyes and then to grip and release the non-paretic hand. Substitute another one step command if the hands cannot be used. Credit is given if an unequivocal attempt is made but not completed due to weakness. If the patient does not respond to command, the task should be demonstrated to him/her (pantomime), and the result scored (i.e., follows none, one or two commands). Patients with trauma, amputation, or other physical impediments should be given suitable one-step commands. Only the first attempt is scored.	0 = **Answers both tasks correctly**.<br>1 = **Answers one task correctly**.<br>2 = **Answers neither task correctly**.	________
**2. Best Gaze**: Only horizontal eye movements will be tested. Voluntary or reflexive (oculocephalic) eye movements will be scored, but caloric testing is not done. If the patient has a conjugate deviation of the eyes that can be overcome by voluntary or reflexive activity, the score will be 1. If a patient has an isolated peripheral nerve paresis (CN III, IV or VI), score a 1. Gaze is testable in all aphasic patients. Patients with ocular trauma, bandages, pre-existing blindness, or other disorder of visual acuity or fields should be tested with reflexive movements, and a choice made by the investigator. Establishing eye contact and then moving about the patient from side to side will occasionally clarify the presence of a partial gaze palsy.	0 = **Normal.**<br>1 = **Partial gaze palsy**; gaze is abnormal in one or both eyes, but forced deviation or total gaze paresis is not present.<br>2 = **Forced deviation**, or total gaze paresis not overcome by the oculocephalic maneuver.	________
**3. Visual:** Visual fields (upper and lower quadrants) are tested by confrontation, using finger counting or visual threat, as appropriate. Patients may be encouraged, but if they look at the side of the moving fingers appropriately, this can be scored as normal. If there is unilateral blindness or enucleation, visual fields in the remaining eye are scored. Score 1 only if a clear-cut asymmetry, including quadrantanopia, is found. If patient is blind from any cause, score 3. Double simultaneous stimulation is performed at this point. If there is extinction, patient receives a 1, and the results are used to respond to item 11.	0 = **No visual loss**.<br>1 = **Partial hemianopia**.<br>2 = **Complete hemianopia**.<br>3 = **Bilateral hemianopia** (blind including cortical blindness).	________
**4. Facial Palsy:** Ask – or use pantomime to encourage – the patient to show teeth or raise eyebrows and close eyes. Score symmetry of grimace in response to noxious stimuli in the poorly responsive or non-comprehending patient. If facial trauma/bandages, orotracheal tube, tape or other physical barriers obscure the face, these should be removed to the extent possible.	0 = **Normal** symmetrical movements.<br>1 = **Minor paralysis** (flattened nasolabial fold, asymmetry on smiling).<br>2 = **Partial paralysis** (total or near-total paralysis of lower face).<br>3 = **Complete paralysis** of one or both sides (absence of facial movement in the upper and lower face	________
**5. Motor Arm:** The limb is placed in the appropriate position: extend the arms (palms down) 90 degrees (if sitting) or 45 degrees (if supine). Drift is scored if the arm falls before 10 sounds. The aphasic patient is encouraged using urgency in the voice and pantomime, but not noxious stimulation. Each limb is tested in turn, beginning with the non-paretic arm. Only in the case of amputation or joint fusion at the shoulder, should the examiner record the score as untestable (UN), and clearly write the explanation for this choice.	0 = **No drift**; limb holds 90 (or 45) degrees for full 10 seconds.<br>1 = **Drift**; limb holds 90 (or 45) degrees, but drifts down before full 10 seconds; does not hit bed or other support.<br>2 = **Some effort against gravity**; limb cannot get to or maintain (if cued) 90 (or 45) degrees, drifts down to bed, but has some effort against gravity.<br>3 = **No effort against gravity**; limb falls.<br>4 = **No movement**.<br>UN= **Amputation** or joint fusion; explain: ______________________<br>5a= **Left Arm**.<br>5b= **Right Arm**.	________<br>________

NIHSS page 4

Instructions	Scale Definition	Score
**10. Dysarthria:** If patient is thought to be normal, an adequate sample of speech must be obtained by asking patient to read or repeat words from the attached list. If the patient has severe aphasia, the clarity of articulation of spontaneous speech can be rated. Only if the patient is intubated or has other physical barriers to producing speech, should the examiner record the score as untestable (UN), and clearly write an explanation for this choice. Do not tell the patient why he or she is being tested.	0 = **Normal**.<br>1 = **Mild-to-moderate dysarthria**; patient slurs at least some words and, at worst, can be understood with some difficulty.<br>2 = **Severe dysarthria**; patient's speech is so slurred as to be unintelligible in the absence of or out of proportion to any dysphasia, or is mute/anarthric.<br>UN= **Intubated** or other physical barrier; explain: ______________________	______
**11. Extinction and Inattention (formerly Neglect):** Sufficient information to identify neglect may be obtained during the prior testing. If the patient has a severe visual loss preventing visual double simultaneous stimulation, and the cutaneous stimuli are normal, the score is normal. If the patient has aphasia but does appear to attend to both sides, the score is normal. The presence of visual spatial neglect or anosagnosia may also be taken as evidence of abnormality. Since the abnormality is scored only if present, the item is never untestable.	0 = **No abnormality**.<br>1 = **Visual, tactile, auditory, spatial, or personal inattention** or extinction to bilateral simultaneous stimulation in one of the sensory modalities.<br>2 = **Profound hemi-inattention or extinction to more than one modality**; does not recognize own hand or orients to only one side of space.	______

NIHSS page 5

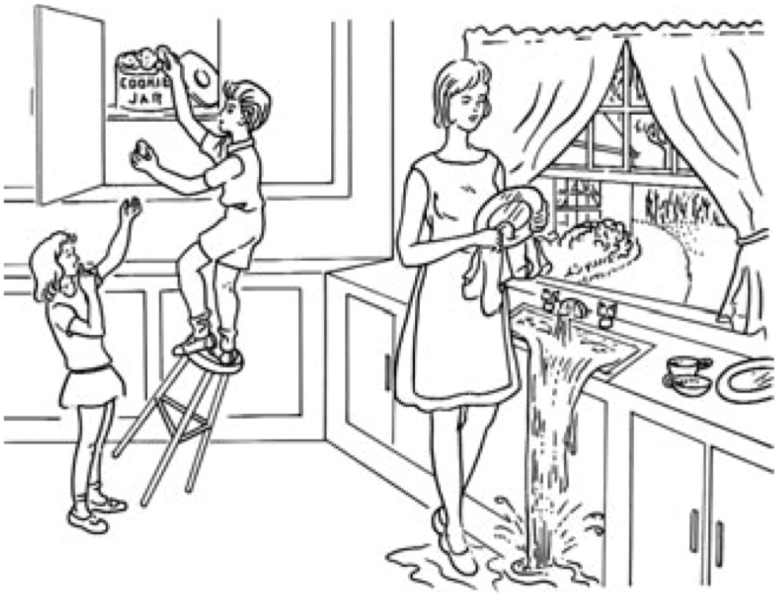

You know how.

Down to earth.

I got home from work.

Near the table in the dining room.

They heard him speak on the radio last night.

请您读出下列句子：
知道
下楼梯
回家做饭
在学校复习
发表精彩演讲

NIHSS page 6

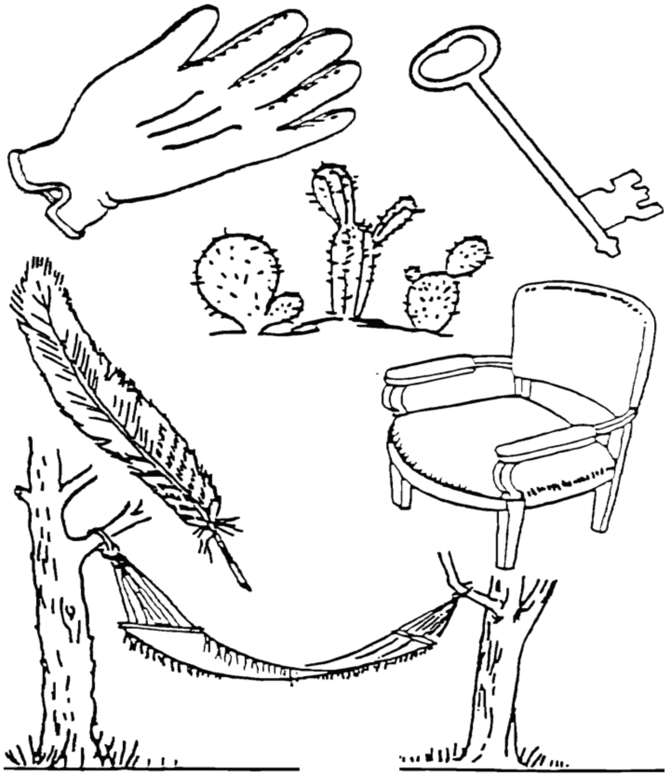

MAMA

TIP – TOP

FIFTY – FIFTY

THANKS

HUCKLEBERRY

BASEBALL PLAYER

**请您读出下列单词：**
**妈妈**
**大地**
**飞机飞机**
**丝绸**
**按时开工**
**吃葡萄不吐葡萄皮**

24.3 Appendix 3: EQ-5D

Subject ID ___ ___ ___ ___ ___ ___ ___ ___ ___ ___
Subject Date of Birth ___ ___/___ ___/___ ___
Hospital ID ______________________________
Date of Examination ___ ___/___ ___/___ ___

Health Questionnaire (EQ-5D-5L)

Under each heading, please tick the ONE box that best describes your health TODAY.

MOBILITY

☐ 1 I have no problems in walking about
☐ 2 I have slight problems in walking about
☐ 3 I have moderate problems in walking about
☐ 4 I have severe problems in walking about
☐ 5 I am unable to walk about

SELF-CARE

☐ 1 I have no problems washing or dressing myself
☐ 2 I have slight problems washing or dressing myself
☐ 3 I have moderate problems washing or dressing myself
☐ 4 I have severe problems washing or dressing myself
☐ 5 I am unable to wash or dress myself

USUAL ACTIVITIES *(e.g. work, study, housework, family or leisure activities)*

☐ 1 I have no problems doing my usual activities
☐ 2 I have slight problems doing my usual activities
☐ 3 I have moderate problems doing my usual activities
☐ 4 I have severe problems doing my usual activities
☐ 5 I am unable to do my usual activities

PAIN / DISCOMFORT

☐ 1 I have no pain or discomfort
☐ 2 I have slight pain or discomfort
☐ 3 I have moderate pain or discomfort
☐ 4 I have severe pain or discomfort
☐ 5 I have extreme pain or discomfort

ANXIETY / DEPRESSION

☐ 1 I am not anxious or depressed
☐ 2 I am slightly anxious or depressed
☐ 3 I am moderately anxious or depressed
☐ 4 I am severely anxious or depressed
☐ 5 I am extremely anxious or depressed

To help people say how good or bad a health state is, we have drawn a scale (rather like a thermometer) on which the best state you can imagine is marked 100 and the worst state you can imagine is marked 0.

We would like you to indicate on this scale how good or bad your own health is today, in your opinion. Please do this by drawing a line from the box below to whichever point on the scale indicates how good or bad your health state is today.

Your own health state today

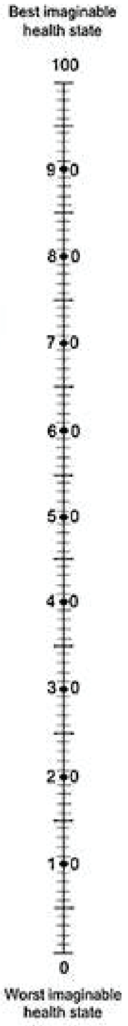

3

UK (English) © 1990 EuroQol Group EQ-5D™ is a trade mark of the EuroQol Group

Summary of Changes - MARVEL Protocol Version 1.0 to Version 2.0

Below is the table of changes. Deleted items are identified with Strikethrough font. Additional wording is in bold font

Section(s)	Protocol Version 1.0<br>Change From:	Protocol Version 2.0<br>Change To:	Rationale
**Section 1. Study Synopsis Inclusion Criteria**	• Baseline Alberta Stroke Program Early CT Score (ASPECTS)>= 5;	Changed:<br>• Baseline Alberta Stroke Program Early CT Score (ASPECTS)>= ~~5~~**3**;	Addition,<br>In light of recent advancements in the management of patients with large infarcts (ASPECTS 3-5), the RESCUE-JAPAN limit trial and several observational studies have demonstrated the superior effectiveness of EVT in treating patients with large infarcts. Additionally, corticosteroids have been suggested to potentially benefit patients with large infarcts.

			To enhance the generalizability and applicability of this study, we have expanded the study population in alignment with the objectives of this research.
**Section 9.1. Study Synopsis Inclusion Criteria**	● Baseline Alberta Stroke Program Early CT Score (ASPECTS)>= 5;	Changed:<br>● Baseline Alberta Stroke Program Early CT Score (ASPECTS)>= ~~5~~3;	Addition,<br>In light of recent advancements in the management of patients with large infarcts (ASPECTS 3-5), the RESCUE-JAPAN limit trial and several observational studies have demonstrated the superior effectiveness of EVT in treating patients with large infarcts. Additionally, corticosteroids have been suggested to potentially benefit patients with large infarcts.

			To enhance the generalizability and applicability of this study, we have expanded the study population in alignment with the objectives of this research.

STATISTICAL ANALYSIS PLAN

MARVEL： Methylprednisolone for acute large vessel occlusion: a randomized double-blind, placebo-controlled trial in revascularization patients

Principal Investigators

Wenjie Zi, MD and Qingwu Yang, PhD., MD

Xinqiao Hospital, Army Medical University, Chongqing, China

Supplement 2 – Statistical Analysis Plan

This supplement contains the following items:

1. Original Statistical Analysis Plan (Pages 2 to 35)
2. Final Statistical Analysis Plan (Pages 36 to 71)
3. Summary of Changes (Pages 72 to 83)

STATISTICAL ANALYSIS PLAN

MARVEL： Methylprednisolone for acute large vessel occlusion: a randomized double-blind, placebo-controlled trial in revascularization patients

Principal Investigators

Wenjie Zi, MD and Qingwu Yang, PhD., MD

Xinqiao Hospital, Army Medical University, Chongqing, China

SAP Authors

Wenjie Zi, MD, PhD; Changwei Guo, MD; Xinqiao Hospital, Army Medical University, Chongqing, China

Duolao Wang, PhD, Global Health Trials Unit, Liverpool School of Tropical Medicine, Liverpool, United Kingdom

Mengjie Lu, PhD, School of Public Health, Shanghai JiaoTong University School of Medicine, Shanghai, China

CONFIDENTIAL

SAP version history		
Version Date	SAP Version #	Details of Changes
2021/09/29	*1.0*	

TABLE OF CONTENTS

Abbreviations and Definitions of Terms 6

1. PREFACE 8

2. PURPOSE OF SAP 8

3. VALIDATION OF SAP AND AMENDMENTS 9

4. STUDY OBJECTIVES AND ENDPOINTS 9

4.1 Study Objectives 9
4.2 Study Endpoints (Target Variables) 9
4.2.1 Primary Efficacy and Safety endpoints 10
4.2.1.1 Primary Efficacy Endpoint 10
4.2.1.2 Primary Safety Endpoints 10
4.2.2 Secondary Efficacy and safety endpoints 10
4.2.2.1 Secondary Efficacy Endpoints 10
4.2.2.2 Secondary Safety Endpoints 11

5. STUDY METHODS 11

5.1 Overall Study Design and Plan 11
5.2 Selection of Study Population 12
5.2.1 Inclusion Criteria 12
5.2.2 Exclusion Criteria 13
5.3 Method of Treatment Assignment and Randomization 14
5.4 Treatment Masking (Blinding) 14
5.5 Contents of Investigational Product Kit 15

6. FORMAL ANALYSES AND REPORTING 16

7. SAMPLE SIZE DETERMINATION 16

8. ANALYSIS POPULATIONS 17

8.1 Intention-to-Treat (ITT) Population 17
8.2 Per-Protocol (PP) Population 18
8.3 Safety Population 18

9. GENERAL ISSUES FOR STATISTICAL ANALYSIS 19

9.1 Analysis Software 19
9.2 Methods for Withdrawals and Missing Data 19
9.2.1 Withdrawals 19
9.2.2 Missing data 19
9.2.2.1 Baseline covariates 19
9.2.2.2 Efficacy outcomes 20
9.3 Data Transformations 20

9.4 Multicenter Studies 20
9.5 Multiple Comparisons and Multiplicity 21
9.6 Covariates 21
9.7 Planned Subgroups 21
9.8 Derived and Computed Variables 23
9.9 Presentation of results 23

10. DISPOSITION OF SUBJECTS AND WITHDRAWALS 23

11. DEMOGRAPHICS AND BASELINE CHARACTERISTICS 23

12. EFFICACY ANALYSES 24

12.1 Primary Efficacy Endpoint 25
12.1.1 Hypothesis 25
12.1.2 Crude Analysis 26
12.1.3 Covariates Adjusted Analysis 26
12.1.4 Subgroup analysis 26
12.1.5 Sensitivity analysis 27
12.2 Secondary Efficacy Analysis 28
12.2.1 Dicotomized modified Rankin Scale score. 29
12.2.2 NIHSS score at 5-7 days or at early discharge 29
12.2.3 European Quality Five-Dimension (EQ-5D) 30

13. SAFETY ANALYSES 30

13.1 Mortality at 90 days 30
13.1.1 Defined of endpoint 30
13.1.2 Statistical Analysis 31
13.2 SICH rate within 48 hours 31
13.2.1 Defined of endpoint 31
13.2.2 Statistical Analysis 32
13.3 Any ICH within 48 hours 32
13.4 Proportion of patients with pneumonia 32
13.5 Proportion of patients with gastrointestinal bleeding within 7 days after EVT 32
13.6. ADVERSE EVENTS 33
13.6.1 Adverse Events 33
13.6.2 Serious Adverse Events 33

References 35

Abbreviations and Definitions of Terms

AE	Adverse Event
AIS	Acute ischemic stroke
ASPECTS	Alberta Stroke Program Early CT Score
CI	Confidence Interval
CRF	Case Report Form
CT	Computed Tomography
CTA	CT angiography
DSA	digital subtraction angiography
DSMB	Data Safety Monitoring Board
EQ-5D-5L	European Quality of Life Five-Dimension Five-Level scale
EVT	Endovascular Therapy
GCP	Good Clinical Practice
GEE	Generalized Estimating Equation
GLM	Generalized Linear Model
ICH	Intracranial Haemorrhage
ICH-E9	International Conference on Harmonization - Statistical principles for clinical trials
ITT	Intention-to-Treat
IVT	Intravenous Thrombolysis
MR	Magnetic Resonance
MRA	MR angiography
mRS	Modified Rankin Scale
NIHSS	National Institute of Health Stroke Scale
NMPA	National Medical Products Administration
PI	Principal Investigator
PP	Per-protocol
SAE	Serious Adverse Event
SAP	Statistical Analysis Plan

SICH	Symptomatic Intracranial Haemorrhage
SOC	System Organ Class
TIA	Transient Ischemic Attack
tPA	Tissue Plasminogen Activator

1. PREFACE

This Statistical Analysis Plan (SAP) describes the planned analysis and reporting for protocol ChiCTR2100051729 entitled "Methylprednisolone for acute large vessel occlusion: a randomized double-blind, placebo-controlled trial in revascularization patients" (Version 1.0, 2022/9/29).

This study is being completed to assess the efficacy and safety of adjunctive intravenous methylprednisolone compared with placebo for the treatment of acute ischemic stroke (AIS) in patients with large vessel occlusion (LVO) underwent endovascular treatment (EVT).

The structure and content of this SAP provides sufficient details to meet the requirements determined by the National Medical Products Administration (NMPA) and International Conference on Harmonization of Technical Requirements for Registration of Pharmaceuticals for Human Use (ICH-E9): Guidance on Statistical Principles in Clinical Trials[1]. All work planned and reported for this SAP will follow internationally recognized guidelines, published by the American Statistical Association[2] and the Royal Statistical Society[3], for statistical practice.

In preparing this SAP, the following documents were reviewed:

- Clinical Study Protocol Version
- Case report forms (CRFs).
- ICH-E9 Guidance on Statistical Principles for Clinical Trials.
- Guidelines for the Content of Statistical Analysis Plans in Clinical Trials

Readers of this SAP are encouraged to also read the clinical trial protocol to understand the implementation details of this study, the operational aspects of clinical evaluation, and the schedule of patients completing this study.

2. PURPOSE OF SAP

The purpose of this SAP is to outline the planned analyses to be completed to support the completion of the clinical study report. The planned analyses identified in this SAP will be included in regulatory submissions and/or future manuscripts. In addition, exploratory analyses not necessarily identified in this SAP may be conducted to support the clinical development plan. Any post-hoc, or unplanned, analyses outside of this SAP will be clearly identified in the respective clinical study report.

3. VALIDATION OF SAP AND AMENDMENTS

The SAP should be validated and signed by:

Principal investigator(s)

Chair of Steering Committee

Chair of Data Safety and Monitoring Committee

Senior statistician

After validation, the SAP may be amended, and all changes will be clearly tracked. Any amendments can occur only before the database is locked and the treatment code is unblinded.

4. STUDY OBJECTIVES AND ENDPOINTS

4.1 Study Objectives

The primary objective is to test the efficacy and safety of adjunctive intravenous methylprednisolone compared with placebo for the treatment of acute ischemic stroke in patients with large vessel occlusion underwent endovascular treatment.

4.2 Study Endpoints (Target Variables)

4.2.1 Primary Efficacy and Safety endpoints

4.2.1.1 Primary Efficacy Endpoint

The primary efficacy outcome measure for this study is: The distribution of the global disability defined as a modified Rankin Scale (mRS) score at Day 90 (±7) after randomization.

4.2.1.2 Primary Safety Endpoints

The primary safety outcome measure for this study are as follows:

- Mortality due to any cause within 90 (±7) days after randomization
- Proportion of patients with symptomatic intracranial haemorrhage within 48 h after treatment. Symptomatic intracranial haemorrhage will be adjudicated by an independent Imaging Core Laboratory according to the modified Heidelberg Bleeding Classification, as specified in the trial protocol. SICH defined as a new ICH seen on imaging will be later confirmed on independent reading by blinded central study radiologists

4.2.2 Secondary Efficacy and safety endpoints

4.2.2.1 Secondary Efficacy Endpoints

The secondary efficacy outcomes are as follows:

The secondary endpoints include:

1) proportion of mRS score 0 to 4 at 90 days;

2) proportion of mRS score 0 to 3 at 90 days

3) proportion of mRS score 0 to 2 at 90 days;

4) proportion of mRS score 0 to 1 or return to pre-morbid mRS score at 90 days (for patients with mRS >1) ;

5) NIHSS score at 5-7 days after EVT or at early discharge;

6) European Quality Five-Dimension scale score at 90 days.

4.2.2.2 Secondary Safety Endpoints

The secondary safety outcomes measure for this study are as follows:

- Proportion of patients with any radiologic intracranial haemorrhage (based on central neuroimaging reading) within 48 hours after receiving intravenous study drug;
- Proportion of patients with pulmonary infection;
- Proportion of patients with gastrointestinal bleeding within 7 days after EVT;
- Proportion of patients with any adverse events;
- Proportion of patients with serious adverse events;

5. STUDY METHODS

5.1 Overall Study Design and Plan

MARVEL is a multi-center, randomised, double-blinded, clinical trial. Patients will be randomized 1:1 to either intravenous methylprednisolone or intravenous placebo. It is planned to recruit at least 1672 patients in about 80 sites in China. The follow-up period covers the period from the subacute stage (day 5-7 or hospital discharge) to the final follow-up examination 83-97 days after randomization which defines the primary endpoint. The study patient flow outline following Consort diagram is shown in Figure 1.

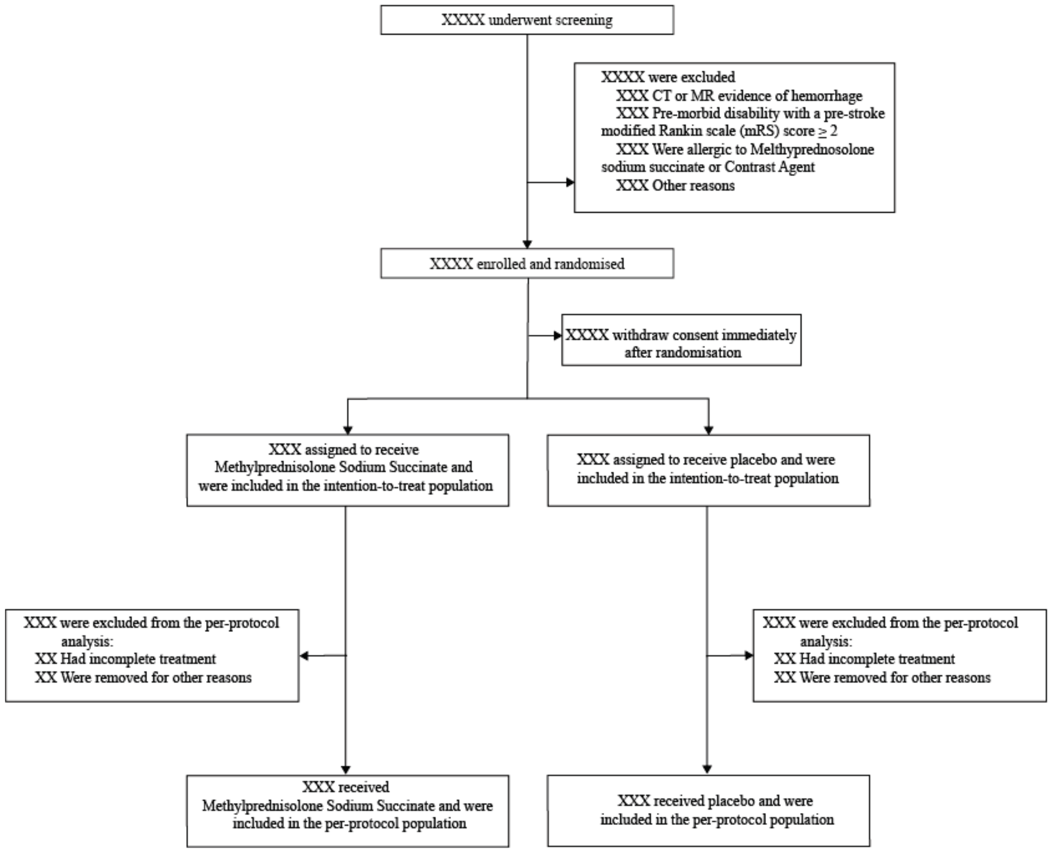

Figure 1. The study patient flow outline following Consort diagram.

5.2 Selection of Study Population

5.2.1 Inclusion Criteria

- Aged 18 years or older;
- The time from onset to randomization was within 24 hours;
- Baseline National Institutes of Health Stroke Scale (NIHSS) ⩾ 6
- Anterior circulation ischemic stroke was preliminarily determined according to clinical symptoms or imaging examination;
- Baseline Alberta Stroke Program Early CT Score (ASPECTS) ⩾ 5;

- Computed tomography angiography (CTA) /magnetic resonance angiography MRA) /digital subtraction angiography (DSA) confirmed occlusion of intracranial segment of internal carotid artery and middle cerebral artery, basilar artery and endovascular therapy was decided;
- Written informed consent signed by patients or their family members.

5.2.2 Exclusion Criteria

Patients meeting any of the following criteria will be excluded from study enrolment.

- Intracranial hemorrhage confirmed by cranial computed tomography (CT) or magnetic resonance imaging (MRI);
- mRS score ⩾ 2 before onset;
- Pregnant or lactating women;
- Allergic to contrast agents;
- Allergic to glucocorticoids;
- Participating in other clinical trials;
- Systolic blood pressure > 185 mmHg or diastolic pressure > 110 mmHg, and oral antihypertensive drugs can not control;
- Genetic or acquired bleeding constitution, lack of anticoagulant factors; Or oral anticoagulants and INR > 1.7;
- Blood sugar < 2.8 mmol/L (50 mg/dl) or > 22.2 mmol/L (400 mg/dl), platelet < 90 x 10^9/L;
- The artery is tortuous so that the thrombectomy device cannot reach the target vessel;
- Bleeding history (gastrointestinal and urinary tract bleeding) in recent 1 month;

- Chronic hemodialysis and severe renal insufficiency (glomerular filtration rate < 30 ml/min or serum creatinine > 220 umol/L [2.5 mg/ dL]);
- Life expectancy due to any advanced disease < 6 months;
- Follow-up is not expected to be completed;
- Intracranial aneurysm and arteriovenous malformation;
- Brain tumors with imaging mass effect;
- Severe systemic infectious diseases.

Inclusion and exclusion criteria will be assessed based on information available during the screening period.

5.3 Method of Treatment Assignment and Randomization

Randomization will be done by a web-based APP (Jinlingshu) on mobile phone or computer (https://jinlingshu.com/). The automated system will assign an appropriate set of study medication to each patient. Eligible patients are randomized to treatment with either intravenous methylprednisolone sodium succinate or intravenous placebo with a ratio of 1:1. Randomization will be stratified by participating centre with permutation block size of 4. Randomization will be completely concealed by having both web-based real-time allocation and identical appearance of methylprednisolone sodium succinate and placebo bottles. (All bottles will have a unique number. Subjects will be assigned a random serial number according to the time they were enrolled, and corresponding masked medications will be provided).

The randomization list will be prepared by the independent statistical centre using SAS 9.4. It will only be sent to the data coordination centre responsible for central randomization and data management, the pharmacy centre responsible for treatment packaging, and the centre responsible for blinding emergency patients.

5.4 Treatment Masking (Blinding)

All trial personnel (investigators, their clinical staff, and the data management group) and patients will be blinded to treatment assignment. The Data Safety Monitoring Board (DSMB) will have access to masked data.

In addition, the person in charge of the data management group responsible for managing the programming of the randomized system will be unblinded. If it is necessary to unblind, this person will become the contact person. This person will not participate in data management and will only communicate to unblind when contacted by the medical monitor.

If the treatment code needs to be broken for the safety of the patient, the site principal investigator (PI) will call the medical monitor for all unblinding queries. The medical monitor will discuss with the researcher whether it is necessary to unblind the patient. If the medical monitor determines that the PI should be unblinded for the patient, the medical monitor will contact the person in charge of the data management group by e-mail or phone, instructing the unblinding of the specific patient. Members of the unblinding data management team will provide only to the site PI with allocation information, the unblinding date, site number, PI name, and patient number via email. Any cases unblinded in this way will be recorded in the central file. Only the physician who requests the unblinding will receive the unblinding message. Study drugs will be stopped afterwards. It is not expected that there will be any clinical cases that need to be unblinded. The randomization data will be strictly confidential, and only authorized personnel can access it until the database is locked.

5.5 Contents of Investigational Product Kit

Methylprednisolone sodium succinate and its placebo are manufactured and provided by Lummy Pharmaceutical Group Co., Ltd., Chongqing, China. The medication packages are visually identical (including labelling, dosage form, size, and colour), except for a unique number. Methylprednisolone sodium succinate and its placebo will be packed in sterile, disposable, individually labelled bottles. Each kit has a unique six-digit identification number and will be stored in a safe location at room temperature on

the clinical site with restricted access. All study medication will be manufactured, tested, released, packaged, labelled, and shipped in accordance with Good Manufacturing Practice, Good Clinical Practice (GCP) guidelines, and any national regulatory requirements.

6. FORMAL ANALYSES AND REPORTING

All formal planned analyses identified in the protocol and in this SAP will be performed only after the formal database lock. Before database lock and completion of the final analyses, a blinded data review meeting will be held. Also, no database may be locked, random code unblinded, or analyses completed until this SAP has been approved and signed.

Any post-hoc, exploratory analyses completed to support planned study analyses, which were not identified in this SAP, will be documented and reported in appendices. Any results of these unplanned analyses will also be clearly identified in appendices.

7. SAMPLE SIZE DETERMINATION

The current trial is designed detect a shift on the modified Rankin Scale score, which represents the global disability, to a lower score. The expected distribution of the modified Rankin Scale score, derived from the EVT for acute anterior circulation ischemic stroke (ACTUAL) registry[4] and the Highly Effective Reperfusion evaluated in Multiple Endovascular Stroke Trials (HERMES) collaboration[5], is as follows: 7%, 20%, 16%, 11%, 13%, 8% and 25%.

The sample size was calculated using the cumulative proportion of patients with mRS 0-2. We assume a moderate effect of 7% absolute increase in the cumulative proportion of patients with mRS 0-2 in the intervention group, compared with controls, indicating an odds ratio (OR) of 1.33, which have substantially exceeds the minimal clinically important difference.[6,7] In order to demonstrate the expected

treatment effect with a type-1 error alpha =0.05 (two-tailed) and a power of 80% (beta = 20%). A sample size of n= 1588 patients (n=794 per treatment group) is required.

The intention-to-treat principle will be applied to the primary analysis and, therefore, to safeguard against dilution of the treatment effect associated with an approximate 5% non-adherence rate (due to loss to follow-up, consent withdrawal and other reasons), we initially planned to enrol n=1672 patients (n=836 per treatment group) for this study. This estimation was performed based on PASS (NCSS, LLC. Kaysville, Utah, USA) version 15.0.

8. ANALYSIS POPULATIONS

The following analysis populations are planned for the studies:

8.1 Intention-to-Treat (ITT) Population

The ITT population includes all patients randomized into the trial and who were recorded as receiving any amount of study drug, even if the subject does not receive the correct treatment, or does not follow the protocol until completion[8]. The ITT population will be the primary analysis population for the efficacy endpoints and subjects will be analysed according to the treatment group to which they were assigned at randomization.

The ITT analysis strategy[8] for MARVEL is defined as follows:

- Is based on an ITT design that aims to collect all outcome data on all randomized subjects;
- Includes a main analysis that keeps subjects in their randomized groups, analyses all available outcome data, and is valid under a named plausible assumption about the missing data;
- Includes sensitivity analyses that consider a range of plausible alternative assumptions about the missing data;

- All randomised individuals are included in sensitivity analyses.

8.2 Per-Protocol (PP) Population

The PP population is defined as the subset of the ITT population excluding major protocol violators deemed to have the potential to affect patient outcome in terms of efficacy.

The PP population includes patients who actually received the assigned treatment and do not have major protocol violations or deviations. Major protocol violations or deviations will be identified in a blinded fashion prior to database lock. More specifically, patients with anyone of the following criteria will be excluded from the PP population. These deviations will be determined based on the medical monitors' records, as well as programmatically, using the following criteria at a minimum:

- Received but did not complete treatment with study drug, or dose of study drug administered outside recommended dose.
- Violate inclusion or exclusion criteria.
- Missing essential information.

A list of patients to be excluded from the randomized patients to create the PP-Efficacy analysis will be established and validated by the Steering Committee prior to unblinding.

8.3 Safety Population

The Safety Population includes all patients who received any amount of study drug. In case of violation of the randomization scheme, patients will be classified according to the treatment they actually received. Patients will be assigned to the different populations prior to unblinding of the database. Patients who withdraw informed consent immediately after randomization and are not to receive any treatment should be excluded from Safety Populations.

9. GENERAL ISSUES FOR STATISTICAL ANALYSIS

9.1 Analysis Software

All statistical analyses will be performed using SAS® Software version 9.4 in a Windows environment[9] and R version 4.1.1.

9.2 Methods for Withdrawals and Missing Data

9.2.1 Withdrawals

If any subject withdraws from the study prematurely (before the final visit day 90±7 for evaluation), they are required to complete the withdrawal visit in the Case Report Form. The reasons for withdrawal will be listed in a summary table. Subjects who withdrew before the subject's last follow up (90 ± 7 days) will be included in the analysis. Subjects who do not receive any study drugs after randomization will be excluded from any end-point analysis because no post-baseline data are available.

9.2.2 Missing data

The handling of missing data will follow the principles specified in the ICH-E9[1] and the CPMP/EWP/1776/99Rev1. Guideline on Missing Data in confirmatory trials Guidelines.[10] All efforts will be made to minimize the amount of missing data, particularly the 90-day outcome assessment. After the patients are randomized, the patients or their families and doctors will keep WeChat (a Chinese social networking software similar to Facebook) and phone numbers for each other to avoid loss to follow-up.

9.2.2.1 Baseline covariates

Missing baseline covariates will be imputed using simple imputation methods in the covariate adjusted analysis based on the covariate distributions, should the missing values for a particular covariate be less than 5%. For a continuous variable, missing

values will be imputed from random values from a normal distribution with mean and SD calculated from the available sample. For a categorical variable, missing values will be imputed from random values from a uniform distribution with probabilities P_1, P_2, …, and P_k from the sample.

If the missing values for a covariate are ≥5% then they will be imputed using Markov chain Monte Carlo (MCMC) methods using SAS PROC MI. A total of 10 multiply imputed dataset will be generated. The seed for the imputations will be 128.

9.2.2.2 Efficacy outcomes

Sensitivity analyses based on different hypotheses about the missingness pattern of the primary outcome will be performed to test for the robustness of the primary analysis results[11]. Primary analysis of the primary outcome will be conducted in the complete case of the ITT population (in which patients with missing data will be censored).

9.3 Data Transformations

Data transformation is not relevant for most of the primary and secondary endpoints, which are categorical variables. The analysis of continuous variables may require transformation to normalize the distributions, but these variables will be mainly analysed using nonparametric statistical tests. Decisions to transform the distribution of some variables for analysis may be taken after the blind review of the data, and before unblinding the treatment code.

9.4 Multicenter Studies

No adjustment on centre will be performed for the primary analysis, on the basis of the following rationale:

The MARVEL trial aims to recruit 1672 patients from about 80 stroke centres in China, about 24 patients per centre. However, it is likely that many centres will recruit far fewer patients, while a few large centres will recruit far more patients. This situation is

the argument for not adjusted the primary analysis on centres. As the International Conference on Harmonization 9 guidelines quotes:

"In some trials, for example some large mortality trials with very few subjects per centre, there may be no reason to expect the centres to have any influence on the primary or secondary variables because they are unlikely to represent influences of clinical importance."

However, the central effect will be considered in the sensitivity analysis together with other important prognostic covariates.

9.5 Multiple Comparisons and Multiplicity

Analyses of secondary safety outcomes and additional analyses for the primary outcome are regarded as exploratory in nature, therefore, multiplicity adjustment will not apply to the primary and secondary outcome analyses.

9.6 Covariates

In the covariates-adjusted analysis, the following variables will be adjusted using the IPTW method.

- Age
- Baseline NIHSS score
- Pre-stroke modified Rankin Scale score
- Baseline aspects score
- Intravenous Thrombolysis
- Time from stroke onset to randomization
- Occlusion location

The same IPTW weight will be used for all adjusted analyses.

9.7 Planned Subgroups

Subgroup analyses could be carried out irrespective of whether there is a significant

treatment effect on the outcome. Their purpose is to supplement evidence from the primary analysis to help to fully characterize the treatment effect. Results from subgroup analyses will be interpreted in this context.

Subgroup analyses will be performed on a few important patient characteristics and other variables of interest. Pre-planned subgroup analyses will explore the effects of:

- Age
- Sex
- Baseline NIHSS score
- Pre-stroke mRS
- Baseline ASPECTS score
- Intravenous thrombolysis
- Time from last known well to randomization. (mins)
- Occlusion location
- Complete Reperfusion defined as Extended Thrombolysis In Cerebral Infarction grade 2c or 3
- Patients with any radiological haemorrhages
- Patients with symptomatic haemorrhages

Subgroup analyses will be conducted irrespective of whether there is a significant treatment effect on the outcome. The purpose is to supplement evidence from the primary analysis to help to fully characterize the treatment effect. These analyses are exploratory and will not lead to formal inferential conclusions about the treatment effect in any particular subgroup.

A forest plot will be used to present the treatment effect of methylprednisolone for each of the individual subgroups. The plot will show the cOR described above with two-sided 95%CIs, corresponding to each level of these subgroups. Separately, the adjusted

estimates for each of the patient characteristics defining a subgroup will be presented from the multivariate model.

9.8 Derived and Computed Variables

Some derived and computed variables have been initially identified and described as appropriate later in the document. It is expected that additional variables will be required. The SAP will not be amended for additional variables that are not related to the primary target or key secondary target variables.

9.9 Presentation of results

Description of continuous variables includes the mean, standard deviation, median, first and third quartiles, and range. The description of categorical variables includes the frequency and percentage n (%). The description of ordered variables includes the frequency, percentage and cumulative percentage.

10. DISPOSITION OF SUBJECTS AND WITHDRAWALS

The number of patients for each of the following categories will be summarized.

- Total number of assessed patients
- Number of randomized patients
- Number of patients completing the study and not completing the study (grouped by treatment and main reason)
- Number of patients included in the ITT population
- Number of patients included in the PP population

11. DEMOGRAPHICS AND BASELINE CHARACTERISTICS

Clinically important demographic and disease characteristics at screening will be summarized with descriptive statistics for each treatment group. Summaries will be produced for the ITT population and PP population.

If the missing value exceeds 5%, the denominator will be added in the footnote of the corresponding summary table.

The variables to be summarized include but are not restricted to the following:

Age

Sex

Medical History (hypertension, coronary heart disease, smoking, diabetes mellitus, hyperlipidaemia, and prior stroke)

Pre stroke onset mRS score

Baseline NIHSS score

Baseline ASPECTS Score

Systolic and Diastolic Blood Pressure

Stroke Aetiology

Serum Glucose

Occlusion Sites

Intravenous Thrombolysis

ASITN/SIR

Time From Stroke Onset (Last Known Well) to Randomization.

Time From Stroke Onset (Last Known Well) to Puncture

Time From Puncture to Reperfusion or the End of the Operation

Time From Randomization to Initiation of study drugs.

12. EFFICACY ANALYSES

All efficacy analyses will provide the point estimate of treatment effect with associated two-sided 95% confidence intervals (CIs). Statistical analyses of the primary and

secondary efficacy endpoints will primarily be based on the ITT population. Secondarily, the same analyses will be repeated on the PP population.

Assessment of efficacy will be conducted based on endpoints collected during the Day 90 follow-up. The protocol allows this visit to be scheduled within 7 days of the target 90 days after randomization.

12.1 Primary Efficacy Endpoint

The mRS score is an assessment of disability with values from 0 (no symptoms at all) to 5 (sever disability) to 6 (death). The mRS score is collected at 90 days after randomization. If death occurs prior to an assessment day (Day 90), the mRS score will be considered available and will be set to 6.

As the primary endpoint, mRS score at Day 90 will be further explored on the range of the values (0 to 6). The distribution of full range of mRS scores at Day 90 will be summarized by treatment group. Both tabular displays and stacked bar charts will be prepared. Win ratio method will be used to calculate the win ratio statistic as a measurement of treatment effect in mRS score.[12] Primary analysis of the primary outcome will be conducted in the complete case of the ITT population (in which patients with missing data will be deleted) using the proportional odds model.

12.1.1 Hypothesis

The null and alternative hypotheses to be tested appear below:

The null hypothesis for proportional odds logistic regression is that the common OR is equal to one, which needs to be disproved. The null hypothesis for WMW test is the equality of ranks when ties are split evenly. This can be rephrased as both probabilities Prob ($Y_M > Y_P$) + 0.5Prob ($Y_M = Y_P$) and Prob ($Y_M < Y_P$) + 0.5Prob ($Y_M = Y_P$) being equal to 0.5, resulting in WMW GenOR being equal to 1. Therefore, the null hypothesis for the WMW test states that there's an equal probability of treatment observation being worse or better than control observation (with equally splitting ties), meaning that

WMW GenOR isn't different from one.

12.1.2 Crude Analysis

The categorical shift in the distribution of mRS scores between the two treatment groups will be analysed fitting a proportional-odds logistic regression model, assuming a common odds ratio across all cut points of the modified Rankin scale.

a) If the proportional odds assumptions are satisfied (both Brant test and Approximate likelihood-ratio test of proportionality of odds are not significant), common OR and 95%CI will be calculate from an GLM with multinomial distribution and cumlogit-link(ordinal logistic regression model) [13,14];

b) If the proportional odds assumptions are not satisfied for the crude analysis (significant Brant test or Approximate likelihood-ratio test of proportionality of odds), assumption-free method will be used.[15]

12.1.3 Covariates Adjusted Analysis

a) If the proportional odds assumptions are satisfied for crude analysis, and still satisfied when adding the covariates in the ordinal logistic regression model, then the common odds ratio from an ordinal logistic regression model, IPTW method will be employed in the co-variate adjusted analysis. [13,14,16]

b) If the proportional odds assumptions are not satisfied for the IPTW adjusted analysis (significant Brant test or Approximate likelihood-ratio test of proportionality of odds), assumption-free method will be used.[15]

Covariate adjusted analysis will be performed on both ITT and PP populations.

Main conclusion will be drawn from the adjusted ITT analysis of the primary endpoint.

12.1.4 Subgroup analysis

Subgroup analysis of the primary endpoint will be performed using modified Poisson regression model on the following subgroup variables:

- age
- baseline NIHSS score
- pre-stroke mRS
- baseline ASPECTS score
- intravenous thrombolysis (yes vs. no)
- time from last known well to randomization. (mins)
- occlusion location
- sex
- pre-stroke mRS
- Complete Reperfusion defined as Extended Thrombolysis in Cerebral Infarction grade 2c or 3
- Patients with any radiological haemorrhages
- Patients with symptomatic haemorrhages

Assessment of the homogeneity of treatment effect by a subgroup variable will be conducted by a GLM with the treatment, subgroup variable, and their interaction term as predictors, adjusted for age, baseline NIHSS score, pre-stroke mRS, baseline ASPECTS score, use of intravenous thrombolysis, time from onset to randomization, and occlusion location, and the *P*-value from the interaction term will be used to assess exploratorily homogeneity of treatment effect by a subgroup variable.

Subgroup analysis will be performed on both ITT and PP populations.

12.1.5 Sensitivity analysis

To assess the influence of the missing primary endpoints on the treatment effect estimate, sensitivity analyses will be performed using the same statistical methods as described in Section 12.1.1 and 12.1.2, under the different assumptions of the missing

outcome below:

(1) Worst-case scenario:

All patients with a missing primary endpoint will be considered as having the worst mRS score of 6.

(2) Best-case scenario:

All patients with a missing primary endpoint will be considered as having the best mRS score of 0.

(3) Multiple Imputation

Missing mRS score will be imputed using multiple imputation method via SAS PROC MI. Imputation procedure will be performed under the missing-at-random (MAR) assumption. Fully conditional method (FCS) will be used to predict missing mRS score by fitting an ordered logistic regression model with following predictors: treatment group, age, baseline NIHSS score, pre-stroke mRS, baseline ASPECTS score, use of intravenous thrombolysis, time from onset to randomization, and occlusion location, and last observed NIHSS if available.[17] The seed will be 128. A total of 10 imputed datasets will be generated. cOR will be estimated for each dataset separately, and the pooled together to calculate the final pooled cOR and 95% confidence interval using the Robin's role.

(4) Mixed-effect model: A general linear mixed effect model will be fitted to estimate the common odds ratio between the methylprednisolone sodium succinate and placebo treatment with treatment as fixed effect, age, baseline NIHSS score, pre-stroke mRS, baseline ASPECTS score, use of intravenous thrombolysis, time from onset to randomization, and occlusion location as covariates, and the centre as a random effect. The results will be calculated for each dataset after multiple imputation and combined following the Rubin's Rule.

12.2 Secondary Efficacy Analysis

12.2.1 Dicotomized modified Rankin Scale score.

Secondary efficacy outcomes include mRS 0-4, mRS 0-3, mRS0-2, mRS 0-1(or return to pre-morbid mRS score) and EQ-5D at Day 90. These results will further describe the treatment effect.

The treatment effect will be estimated in the ITT population using a GLM with Poisson distribution and log-link function to determine the risk ratio (RR) and its 95%CI.[18] In case of nonconvergence of this model, the following generalized linear models (GLMs) will be fitted sequentially until a model is converged:

- GLM with Binomial distribution and log link function;
- GLM with Negative Binomial distribution and log link function;
- GLM with Binomial distribution and logit link function, from which odds ratio (OR) will be converted into RR.[16,19]

In addition, risk difference and OR will be estimated and presented using two GLMs (identity-binomial regression and logit-binomial regression) as supportive measurements of treatment effect.

Adjusted analyses will be carried out on the primary efficacy outcome to determine whether the treatment effect estimate is affected with the inclusion of covariates at baseline. The covariates that will be included in the adjusted analyses are listed and described in Section 9.6. Imputation for baseline missing covariates (see description in the Section 9.2

12.2.2 NIHSS score at 5-7 days or at early discharge

The NIHSS is a standardized neurological examination score, with scores ranging from 0 to 42, with higher scores indicating increasing severity.

The mean difference and 95%CI of NIHSS score between two groups will be estimated using a GLM with treatment group as the sole predictor and the NIHSS score at 5-7 days or at early discharge as the dependent variable. Subjects with missing NIHSS score at 5-7 days or at early discharge will be excluded from the analysis. IPTW method will

be employed in calculating the co-variates adjusted mean difference (Co-variates will be defined as Section 9.6). Normality and variance homogeneity of residuals will be accessed graphically. The win ratio method will be used if normality assumption is seriously violated.

12.2.3 European Quality Five-Dimension (EQ-5D)

The EQ-5D is a tool for describing and assessing health-related quality of life 90±7 days after randomization. The tool is designed for self-completion.-The EQ-5D will be completed on the 90 (±7) days after randomization. Reasons for not performing the assessment of EQ-5D at Day 90 will be summarized.

The mean difference and 95%CI of EQ-5D score between two groups will be estimated using a GLM with treatment group as the sole predictor and the 90 days EQ-5D score as the dependent variable. IPTW method will be employed in calculating the co-variates adjusted mean difference and corresponding 95%CI (Co-variates will be defined as Section 9.6).

Subjects with missing EQ-5D score will be excluded from the analysis. Normality and variance homogeneity of residuals will be accessed graphically. The win ratio method will be used if normality assumption is seriously violated.

13. SAFETY ANALYSES

Safety analyses will be conducted on the Safety population only.

13.1 Mortality at 90 days

13.1.1 Defined of endpoint

Mortality due to any cause is the primary safety endpoint. The outcome is measured on a binary scale and is defined as positive in the presence of death of any cause, and as negative otherwise. With the date of randomization at day 0, any death occurring on or before calendar day 90 will be counted as a death. Patients who are alive at day 90 will

be censored at day 90. Mortality rates are defined as the number of deaths observed divided by the number of subjects observed over the 90-day study period.

13.1.2 Statistical Analysis

The modified Poisson regression with a treatment group as an independent variable and the cumulative rate death due to any cause at 90 days as the dependent variable. The treatment effect will be presented as RR with the corresponding 95%CI. IPTW will be used in calculating the adjusted RR and its 95%CI.

In addition, proportional hazards regression model will be employed to calculate the hazard ratio with 95%CI. IPTW method will be used to calculate the adjusted hazard ratio. In addition, the Kaplan-Meier method will be used to display the survival curves by treatment arm. The log-rank test will be applied to compare survival curves between the two treatment groups.

13.2 SICH rate within 48 hours

13.2.1 Defined of endpoint

Proportion of patients with symptomatic intracranial haemorrhage (SICH) within 48 hours post-treatment is considered another primary safety outcome. SICH will be evaluated according to the Heidelberg Bleeding Classification[20].

13.2.2 Statistical Analysis

Percentage of subjects with SICH within 48 hours after treatment will be presented for each definition by treatment group. Frequency counts and percentage of patients within each category will be provided for categorical data. Subject rates will be compared between treatment groups with Chi-square test or Fisher's exact test.

The modified Poisson Regression model will be used to estimates the RR and corresponding 95%CI with treatment group as an independent variable and the presence or absence of SICH as the dependent variable. The treatment effect will be presented as RR with the corresponding 95%CI. IPTW methods will be employed to calculate the adjusted RR and 95%CI.

13.3 Any ICH within 48 hours

The statistical analysis of differences in proportions of patients with any radiologic ICH within 48 hours between methylprednisolone sodium succinate and placebo groups will be performed as per the Section 13.2. SICH rate within 48 hours.

13.4 Proportion of patients with pneumonia

Number of patients and percentage of subjects with pulmonary infection within 48 hours after treatment will be presented by treatment group. The between group difference will be tested using the modified Poisson Model. And the Adjusted RR and corresponding 95% CI will be estimated using the IPTW method as other binary outcomes. Both unadjusted and adjusted results will be presented.

13.5 Proportion of patients with gastrointestinal bleeding within 7 days after EVT

Number of patients and percentage of subjects' gastrointestinal bleeding within 7 days after EVT will be presented by treatment group. The between group difference will be

tested using the modified Poisson Model. The Adjusted RR and corresponding 95%CI will be estimated using the IPTW method as other binary outcomes. Both unadjusted and adjusted results will be presented.

13.6. ADVERSE EVENTS

Adverse events (AEs) and diseases will recorded in this study.

13.6.1 Adverse Events

Any adverse change in health or the appearance of or worsening of any undesirable sign, symptom, or medical condition occurring after enrollment into the trial will be recorded as Adverse Event (AE) whether or not it is considered to be related to the study drug. An AE also includes a new illness; aggravated in severity of frequency from the baseline condition, abnormal results of diagnostic procedures, or a combination of the above. Pre-existing medical conditions are not to be reported as AEs.

13.6.2 Serious Adverse Events

A serious adverse event (SAE) is any untoward medical occurrence that at any dose: Result in death; Are life-threatening or fatal; Require or prolong hospitalization; Result in persistent or significant disability/incapacity, Constitutes a congenital anomaly or birth defect, or Significant medical event.

A SAE can also be an important medical event that may not result in death, be life-threatening, or require hospitalization, but may jeopardize the subject and may require medical or surgical intervention to prevent one of the outcomes listed in this definition. For example, any new diagnosis of cancer (made after study enrollment) is considered an important medical event. Because our primary safety outcomes for the trial are also SAEs, they will be reported dually as SAEs and as outcomes. SAEs should be managed according to the best current standard of care. All deaths occurring during the follow-up to Day 90 will be reported as an SAE. When reporting a death, the event or condition that caused or contributed to the fatal outcome should be reported as a single medical

concept.

References

1. CPMP/ICH/363/96. ICH E9 Statistical Principles for Clinical Trials.
2. Association AS. Ethical Guidelines for Statistical Practice. Prepared by the Committee on Professional Ethics.
3. Society RS. The Royal Statistical Society: Code of Conduct.
4. Zi W, Wang H, Yang D, et al. Clinical Effectiveness and Safety Outcomes of Endovascular Treatment for Acute Anterior Circulation Ischemic Stroke in China. Cerebrovasc Dis 2017;44:248-58.
5. Goyal M, Menon BK, van Zwam WH, et al. Endovascular thrombectomy after large-vessel ischaemic stroke: a meta-analysis of individual patient data from five randomised trials. LANCET 2016;387:1723-31.
6. Cranston JS, Kaplan BD, Saver JL. Minimal Clinically Important Difference for Safe and Simple Novel Acute Ischemic Stroke Therapies. Stroke 2017;48:2946-51.
7. Berkhemer OA, Fransen PSS, Beumer D, et al. A Randomized Trial of Intraarterial Treatment for Acute Ischemic Stroke. New England Journal of Medicine 2015;372:11-20.
8. White IR, Horton NJ, Carpenter J, Pocock SJ. Strategy for intention to treat analysis in randomised trials with missing outcome data. Bmj 2011;342:d40.
9. SAS version 9.4 software, SAS Institute Inc., Cary, NC,.
10. Rev1. CE. Guideline on Missing Data in confirmatory trials.
11. (EMA/CHMP/ICH/436221/2017). ICH E9 (R1) addendum on estimands and sensitivity analysis in clinical trials to the guideline on statistical principles for clinical trials. Draft.
12. Wang D, Pocock S. A win ratio approach to comparing continuous non-normal outcomes in clinical trials. Pharm Stat 2016;15:238-45.
13. Agresti A. Categorical Data Analysis, Second Edition.
14. Saver JL. Novel end point analytic techniques and interpreting shifts across the entire range of outcome scales in acute stroke trials. Stroke 2007;38:3055-62.
15. Churilov L, Arnup S, Johns H, et al. An improved method for simple, assumption-free ordinal analysis of the modified Rankin Scale using generalized odds ratios. International Journal of Stroke 2014;9:999-1005.
16. Morris TP, Walker AS, Williamson EJ, White IR. Planning a method for covariate adjustment in individually randomised trials: a practical guide. Trials 2022;23:328.
17. Young-Saver DF, Gornbein J, Starkman S, Saver JL. Handling of Missing Outcome Data in Acute Stroke Trials: Advantages of Multiple Imputation Using Baseline and Postbaseline Variables. J Stroke Cerebrovasc Dis 2018;27:3662-9.
18. Zou G. A modified poisson regression approach to prospective studies with binary data. Am J Epidemiol 2004;159:702-6.
19. Austin PC, Stuart EA. Moving towards best practice when using inverse probability of treatment weighting (IPTW) using the propensity score to estimate causal treatment effects in observational studies. Stat Med 2015;34:3661-79.
20. von Kummer R, Broderick JP, Campbell BCV, et al. The Heidelberg Bleeding Classification Classification of Bleeding Events After Ischemic Stroke and Reperfusion Therapy. Stroke 2015;46:2981-6.

STATISTICAL ANALYSIS PLAN

MARVEL: Methylprednisolone for acute large vessel occlusion: a randomized double-blind, placebo-controlled trial in revascularization patients

Principal Investigators

Wenjie Zi, MD and Qingwu Yang, PhD., MD

Xinqiao Hospital, Army Medical University, Chongqing, China

SAP Authors

Wenjie Zi, MD, PhD; Changwei Guo, MD; Xinqiao Hospital, Army Medical University, Chongqing, China

Duolao Wang, PhD, Global Health Trials Unit, Liverpool School of Tropical Medicine, Liverpool, United Kingdom

Mengjie Lu, PhD, School of Public Health, Shanghai JiaoTong University School of

Medicine, Shanghai, China

CONFIDENTIAL

SAP version history		
Version Date	SAP Version #	Details of Changes
2021/09/29	*1.0*	
2023/10/01	*2.0*	*Changed the primary analysis of the primary outcome from inverse probability treatment weighting common odds ratio to generalized odds ratio.*

	Signature	Date
Dr. Changwei Guo (Trial Statistician)	Changwei Guo	*2023/10/01*
Prof. Duolao Wang (Senior Statistician)	Duolao Wang	
Prof. Qingwu Yang (Chief Investigator)	Qingwu Yang	
Prof. Yangmei Chen (Chief of the DSMB)	Yangmei Chen	

Prof. Wenjie Zi (Chief Investigator & SC Chair)	Wenjie Zi	

TABLE OF CONTENTS

Abbreviations and Definitions of Terms 40

1. PREFACE 42

2. PURPOSE OF SAP 42

3. VALIDATION OF SAP AND AMENDMENTS 43

4. STUDY OBJECTIVES AND ENDPOINTS 43

4.1 Study Objectives 43
4.2 Study Endpoints (Target Variables) 43
4.2.1 Primary efficacy and safety endpoints 44
4.2.1.1 Primary Efficacy Endpoint 44
4.2.1.2 Primary Safety Endpoints 44
4.2.2 Secondary efficacy and safety endpoints 44
4.2.2.1 Secondary Efficacy Endpoints 44
4.2.2.2 Secondary Safety Endpoints 45

5. STUDY METHODS 45

5.1 Overall Study Design and Plan 45
5.2 Selection of Study Population 46
5.2.1 Inclusion Criteria 46
5.2.2 Exclusion Criteria 47
5.3 Method of Treatment Assignment and Randomization 48
5.4 Treatment masking (Blinding) 48
5.5 Contents of Investigational Product Kit 49

6. FORMAL ANALYSES AND REPORTING 50

7. SAMPLE SIZE DETERMINATION 50

8. ANALYSIS POPULATIONS 51

8.1 Intention-to-Treat (ITT) population 51
8.2 Per-Protocol (PP) population 52
8.3 Safety population 52

9. GENERAL ISSUES FOR STATISTICAL ANALYSIS 53

9.1 Analysis Software 53
9.2 Methods for Withdrawals and Missing Data 53
9.2.1 Withdrawals 53

9.2.2 Missing data 53
9.2.2.1 Baseline covariates 53
9.2.2.2 Efficacy outcomes 54
9.3 Data Transformations 54
9.4 Multicenter Studies 54
9.5 Multiple Comparisons and Multiplicity 55
9.6 Covariates 55
9.7 Planned Subgroups 55
9.8 Derived and Computed Variables 57
9.9 Presentation of results 57
10. DISPOSITION OF SUBJECTS AND WITHDRAWALS 57
11. DEMOGRAPHICS AND BASELINE CHARACTERISTICS 58
12. EFFICACY ANALYSES 59
12.1 Primary Efficacy Endpoint (Ordinal data) 59
12.1.1 Crude analysis 60
12.1.2 Covariates adjusted analysis 61
12.1.3 Sensitivity analysis 61
12.1.4 Supplementary analysis 62
12.1.5 Subgroup analysis 63
12.2 Secondary Efficacy Analysis 64
12.2.1 Dichotomized modified Rankin Scale score. (Binary data) 64
12.2.2 NIHSS score at 5-7 days or discharge if earlier (Continuous) 64
12.2.3 European Quality Five-Dimension (EQ-5D) (Continuous data) 65
13. SAFETY ANALYSES 65
13.1 Mortality at 90 days (Time to Event data) 65
13.1.1 Defined of endpoint 65
13.1.2 Statistical Analysis 66
13.2 SICH rate within 48 hours (Binary data) 66
13.2.1 Defined of endpoint 66
13.2.2 Statistical Analysis 67
13.3 Any ICH within 48 hours (Binary data) 67
13.4 Proportion of patients with newly or deteriorated pulmonary infection (Binary data) 67
13.5 Proportion of patients with gastrointestinal bleeding within 7 days after EVT (Binary data) 67
13.6. ADVERSE EVENTS 68
13.6.1 Adverse Events 68
13.6.2 Serious Adverse Events 68
References 70

Abbreviations and Definitions of Terms

AE	Adverse Event
AIS	Acute ischemic stroke
ASPECTS	Alberta Stroke Program Early CT Score
CI	Confidence Interval
CRF	Case Report Form
CT	Computed Tomography
CTA	CT angiography
DSA	digital subtraction angiography
DSMB	Data Safety Monitoring Board
EQ-5D-5L	European Quality of Life Five-Dimension Five-Level scale
EVT	Endovascular Therapy
GCP	Good Clinical Practice
GEE	Generalized Estimating Equation
GLM	Generalized Linear Model
ICH	Intracranial Haemorrhage
ICH-E9	International Conference on Harmonization - Statistical principles for clinical trials
ITT	Intention-to-Treat
IVT	Intravenous Thrombolysis
MR	Magnetic Resonance
MRA	MR angiography
mRS	Modified Rankin Scale
NIHSS	National Institute of Health Stroke Scale
NMPA	National Medical Products Administration
PI	Principal Investigator
PP	Per-protocol
SAE	Serious Adverse Event
SAP	Statistical Analysis Plan

SICH	Symptomatic Intracranial Haemorrhage
SOC	System Organ Class
TIA	Transient Ischemic Attack
tPA	Tissue Plasminogen Activator

1. PREFACE

This Statistical Analysis Plan (SAP) describes the planned analysis and reporting for protocol ChiCTR2100051729 entitled "Methylprednisolone for acute large vessel occlusion: a randomized double-blind, placebo-controlled trial in revascularization patients" (Version 2.0, 2022/8/18).

This study is being completed to assess the efficacy and safety of adjunctive intravenous methylprednisolone compared with placebo for the treatment of acute ischemic stroke (AIS) in patients with large vessel occlusion (LVO) underwent endovascular treatment (EVT).

The structure and content of this SAP provides sufficient details to meet the requirements determined by the National Medical Products Administration (NMPA) and International Conference on Harmonization of Technical Requirements for Registration of Pharmaceuticals for Human Use (ICH-E9): Guidance on Statistical Principles in Clinical Trials[1]. All work planned and reported for this SAP will follow internationally recognized guidelines, published by the American Statistical Association[2] and the Royal Statistical Society[3], for statistical practice.

In preparing this SAP, the following documents were reviewed:

- Clinical Study Protocol Version
- Case report forms (CRFs).
- ICH-E9 Guidance on Statistical Principles for Clinical Trials.
- Guidelines for the Content of Statistical Analysis Plans in Clinical Trials

Readers of this SAP are encouraged to also read the clinical trial protocol to understand the implementation details of this study, the operational aspects of clinical evaluation, and the schedule of patients completing this study.

2. PURPOSE OF SAP

The purpose of this SAP is to outline the planned analyses to be completed to support the completion of the clinical study report. The planned analyses identified in this SAP will be included in regulatory submissions and/or future manuscripts. In addition, exploratory analyses not necessarily identified in this SAP may be conducted to support the clinical development plan. Any post-hoc, or unplanned, analyses outside of this SAP will be clearly identified in the respective clinical study report.

3. VALIDATION OF SAP AND AMENDMENTS

The SAP should be validated and signed by:

Principal investigator(s)

Chair of Steering Committee

Chair of Data Safety and Monitoring Committee

Senior statistician

After validation, the SAP may be amended, and all changes will be clearly tracked. Any amendments can occur only before the database is locked and the treatment code is unblinded.

4. STUDY OBJECTIVES AND ENDPOINTS

4.1 Study Objectives

The primary objective is to test the efficacy and safety of adjunctive intravenous methylprednisolone compared with placebo for the treatment of acute ischemic stroke in patients with large vessel occlusion underwent endovascular treatment.

4.2 Study Endpoints (Target Variables)

4.2.1 Primary efficacy and safety endpoints

4.2.1.1 Primary Efficacy Endpoint

The primary efficacy outcome measure for this study is: The distribution of the global disability defined as a modified Rankin Scale (mRS) score at Day 90 (±7) after randomization (Ordinal data).

4.2.1.2 Primary Safety Endpoints

The primary safety outcome measure for this study are as follows:

- Mortality due to any cause within 90 (±7) days after randomization (Binary data)
- Proportion of patients with symptomatic intracranial haemorrhage within 48 h after treatment. Symptomatic intracranial haemorrhage will be adjudicated by an independent Imaging Core Laboratory according to the modified Heidelberg Bleeding Classification, as specified in the trial protocol. SICH defined as a new ICH seen on imaging will be later confirmed on independent reading by blinded central study radiologists (Binary data).

4.2.2 Secondary efficacy and safety endpoints

4.2.2.1 Secondary Efficacy Endpoints

The secondary efficacy outcomes are as follows:

The secondary endpoints include:

1) proportion of mRS score 0 to 4 at 90 days (Binary data);

2) proportion of mRS score 0 to 3 at 90 days (Binary data)’

3) proportion of mRS score 0 to 2 at 90 days (Binary data);

4) proportion of mRS score 0 to 1 or return to pre-morbid mRS score at 90 days (for patients with mRS >1) (Binary data);

5) NIHSS score at 5-7 days after EVT or at early discharge; (Continuous data)

6) European Quality Five-Dimension scale score at 90 days (Continuous data).

4.2.2.2 Secondary Safety Endpoints

The secondary safety outcomes measure for this study are as follows:

- Proportion of patients with any radiologic intracranial haemorrhage (based on central neuroimaging reading) within 48 hours after receiving intravenous study drug (Binary data);
- Proportion of patients with pneumonia (Binary data);
- Proportion of patients with gastrointestinal bleeding within 7 days after EVT (Binary data);
- Proportion of patients with any adverse events;
- Proportion of patients with serious adverse events;

5. STUDY METHODS

5.1 Overall Study Design and Plan

MARVEL is a multi-center, randomised, double-blinded, clinical trial. Patients will be randomized 1:1 to either intravenous methylprednisolone or intravenous placebo. It is planned to recruit at least 1672 patients in about 80 sites in China. The follow-up period covers the period from the subacute stage (day 5-7 or hospital discharge) to the final follow-up examination 83-97 days after randomization which defines the primary endpoint. The study patient flow outline following Consort diagram is shown in Figure 1.

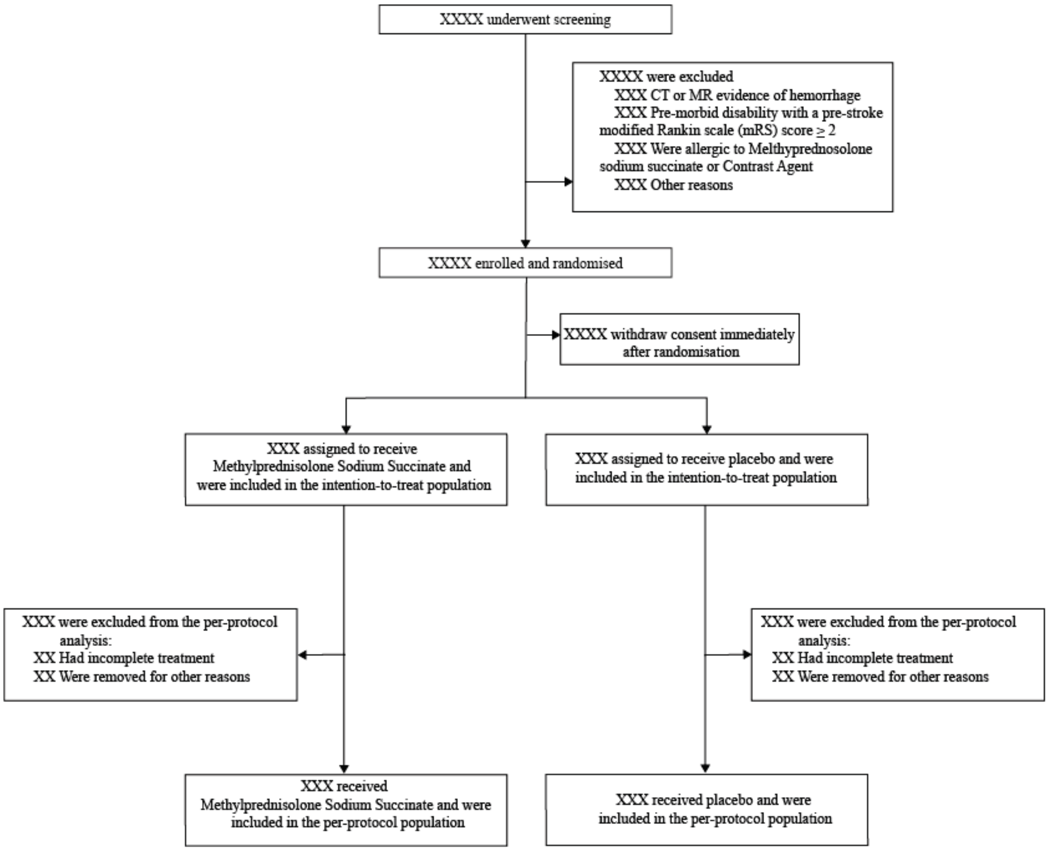

Figure 1. The study patient flow outline following Consort diagram.

5.2 Selection of Study Population

5.2.1 Inclusion Criteria

- Aged 18 years or older;
- The time from onset to randomization was within 24 hours;
- Baseline National Institutes of Health Stroke Scale (NIHSS) ≥ 6
- Anterior circulation ischemic stroke was preliminarily determined according to clinical symptoms or imaging examination;
- Baseline Alberta Stroke Program Early CT Score (ASPECTS) ≥ 3;

- Computed tomography angiography (CTA) /magnetic resonance angiography MRA) /digital subtraction angiography (DSA) confirmed occlusion of intracranial segment of internal carotid artery and middle cerebral artery, basilar artery and endovascular therapy was decided;
- Written informed consent signed by patients or their family members.

5.2.2 Exclusion Criteria

Patients meeting any of the following criteria will be excluded from study enrolment.

- Intracranial hemorrhage confirmed by cranial computed tomography (CT) or magnetic resonance imaging (MRI);
- mRS score ⩾ 2 before onset;
- Pregnant or lactating women;
- Allergic to contrast agents;
- Allergic to glucocorticoids;
- Participating in other clinical trials;
- Systolic blood pressure > 185 mmHg or diastolic pressure > 110 mmHg, and oral antihypertensive drugs can not control;
- Genetic or acquired bleeding constitution, lack of anticoagulant factors; Or oral anticoagulants and INR > 1.7;
- Blood sugar < 2.8 mmol/L (50 mg/dl) or > 22.2 mmol/L (400 mg/dl), platelet < 90 x 10^9/L;
- The artery is tortuous so that the thrombectomy device cannot reach the target vessel;
- Bleeding history (gastrointestinal and urinary tract bleeding) in recent 1 month;

- Chronic hemodialysis and severe renal insufficiency (glomerular filtration rate < 30 ml/min or serum creatinine > 220 umol/L [2.5 mg/ dL]);
- Life expectancy due to any advanced disease < 6 months;
- Follow-up is not expected to be completed;
- Intracranial aneurysm and arteriovenous malformation;
- Brain tumors with imaging mass effect;
- Severe systemic infectious diseases.

Inclusion and exclusion criteria will be assessed based on information available during the screening period.

5.3 Method of Treatment Assignment and Randomization

Randomization will be done by a web-based APP (Jinlingshu) on mobile phone or computer (https://jinlingshu.com/). The automated system will assign an appropriate set of study medication to each patient. Eligible patients are randomized to treatment with either intravenous methylprednisolone sodium succinate or intravenous placebo with a ratio of 1:1. Randomization will be stratified by participating centre with permutation block size of 4. Randomization will be completely concealed by having both web-based real-time allocation and identical appearance of methylprednisolone sodium succinate and placebo bottles. (All bottles will have a unique number. Subjects will be assigned a random serial number according to the time they were enrolled, and corresponding masked medications will be provided).

The randomization list will be prepared by the independent statistical centre using SAS 9.4. It will only be sent to the data coordination centre responsible for central randomization and data management, the pharmacy centre responsible for treatment packaging, and the centre responsible for blinding emergency patients.

5.4 Treatment masking (Blinding)

All trial personnel (investigators, their clinical staff, and the data management group) and patients will be blinded to treatment assignment. The Data Safety Monitoring Board (DSMB) will have access to masked data.

Both the person responsible for investigational product labels and the independent statistical group that prepare reports for the DSMB will be unblinded. In addition, the person in charge of the data management group responsible for managing the programming of the randomized system will be unblinded. If it is necessary to unblind, this person will become the contact person. This person will not participate in data management and will only communicate to unblind when contacted by the medical monitor.

If the treatment code needs to be broken for the safety of the patient, the site principal investigator (PI) will call the medical monitor for all unblinding queries. The medical monitor will discuss with the researcher whether it is necessary to unblind the patient. If the medical monitor determines that the PI should be unblinded for the patient, the medical monitor will contact the person in charge of the data management group by e-mail or phone, instructing the unblinding of the specific patient. Members of the unblinding data management team will provide only to the site PI with allocation information, the unblinding date, site number, PI name, and patient number via email. Any cases unblinded in this way will be recorded in the central file. Only the physician who requests the unblinding will receive the unblinding message. Study drugs will be stopped afterwards. It is not expected that there will be any clinical cases that need to be unblinded. The randomization data will be strictly confidential, and only authorized personnel can access it until the database is locked.

5.5 Contents of Investigational Product Kit

Methylprednisolone sodium succinate and its placebo are manufactured and provided by Lummy Pharmaceutical Group Co., Ltd., Chongqing, China. The medication packages are visually identical (including labelling, dosage form, size, and colour), except for a unique number. Methylprednisolone sodium succinate and its placebo will

be packed in sterile, disposable, individually labelled bottles. Each kit has a unique six-digit identification number and will be stored in a safe location at room temperature on the clinical site with restricted access. All study medication will be manufactured, tested, released, packaged, labelled, and shipped in accordance with Good Manufacturing Practice, Good Clinical Practice (GCP) guidelines, and any national regulatory requirements.

6. FORMAL ANALYSES AND REPORTING

All formal planned analyses identified in the protocol and in this SAP will be performed only after the formal database lock. Before database lock and completion of the final analyses, a blinded data review meeting will be held. Also, no database may be locked, random code unblinded, or analyses completed until this SAP has been approved and signed.

Any post-hoc, exploratory analyses completed to support planned study analyses, which were not identified in this SAP, will be documented and reported in appendices. Any results of these unplanned analyses will also be clearly identified in appendices.

7. SAMPLE SIZE DETERMINATION

The current trial is designed detect a shift on the modified Rankin Scale score, which represents the global disability, to a lower score. The expected distribution of the modified Rankin Scale score, derived from the EVT for acute anterior circulation ischemic stroke (ACTUAL) registry[4] and the Highly Effective Reperfusion evaluated in Multiple Endovascular Stroke Trials (HERMES) collaboration[5], is as follows: 7%, 20%, 16%, 11%, 13%, 8% and 25%.

The sample size was calculated using the cumulative proportion of patients with mRS 0-2. We assume a moderate effect of 7% absolute increase in the cumulative proportion of patients with mRS 0-2 in the intervention group, compared with controls, indicating an odds ratio (OR) of 1.33, which have substantially exceeds the

minimal clinically important difference.[6,7] In order to demonstrate the expected treatment effect with a type-1 error alpha =0.05 (two-tailed) and a power of 80% (beta = 20%). A sample size of n= 1588 patients (n=794 per treatment group) is required.

The intention-to-treat principle will be applied to the primary analysis and, therefore, to safeguard against dilution of the treatment effect associated with an approximate 5% non-adherence rate (due to loss to follow-up, consent withdrawal and other reasons), we initially planned to enrol n=1672 patients (n=836 per treatment group) for this study. This estimation was performed based on PASS (NCSS, LLC. Kaysville, Utah, USA) version 15.0.

8. ANALYSIS POPULATIONS

The following analysis populations are planned for the studies:

8.1 Intention-to-Treat (ITT) population

The ITT population includes all patients randomized into the trial and who were recorded as receiving any amount of study drug, even if the subject does not receive the correct treatment, or does not follow the protocol until completion[8]. The ITT population will be the primary analysis population for the efficacy endpoints and subjects will be analysed according to the treatment group to which they were assigned at randomization.

The ITT analysis strategy[8] for MARVEL is defined as follows:

- Is based on an ITT design that aims to collect all outcome data on all randomized

subjects;

- Includes a main analysis that keeps subjects in their randomized groups, analyses all available outcome data, and is valid under a named plausible assumption about the missing data;
- Includes sensitivity analyses that consider a range of plausible alternative assumptions about the missing data;

- All randomised individuals are included in sensitivity analyses.

8.2 Per-Protocol (PP) population

The PP population is defined as the subset of the ITT population excluding major protocol violators deemed to have the potential to affect patient outcome in terms of efficacy.

The PP population includes patients who actually received the assigned treatment and do not have major protocol violations or deviations. Major protocol violations or deviations will be identified in a blinded fashion prior to database lock. More specifically, patients with anyone of the following criteria will be excluded from the PP population. These deviations will be determined based on the medical monitors' records, as well as programmatically, using the following criteria at a minimum:

- Received but did not complete treatment with study drug, or dose of study drug administered outside recommended dose.
- Violate inclusion or exclusion criteria.
- Missing essential information.

A list of patients to be excluded from the randomized patients to create the PP-Efficacy analysis will be established and validated by the Steering Committee prior to unblinding.

8.3 Safety population

The Safety Population includes all patients who received any amount of study drug. In case of violation of the randomization scheme, patients will be classified according to the treatment they actually received. Patients will be assigned to the different populations prior to unblinding of the database. Patients who withdraw informed consent immediately after randomization and are not to receive any treatment should be excluded from Safety Populations.

9. GENERAL ISSUES FOR STATISTICAL ANALYSIS

9.1 Analysis Software

All statistical analyses will be performed using SAS® Software version 9.4 in a Windows environment[9] and R version 4.3.0.

9.2 Methods for Withdrawals and Missing Data

9.2.1 Withdrawals

If any subject withdraws from the study prematurely (before the final visit day 90±7 for evaluation), they are required to complete the withdrawal visit in the Case Report Form. The reasons for withdrawal will be listed in a summary table. Subjects who withdrew before the subject's last follow up (90 ± 7 days) will be included in the analysis. Subjects who do not receive any study drugs after randomization will be excluded from any end-point analysis because no post-baseline data are available.

9.2.2 Missing data

The handling of missing data will follow the principles specified in the ICH-E9[1] and the CPMP/EWP/1776/99Rev1. Guideline on Missing Data in confirmatory trials Guidelines.[10] All efforts will be made to minimize the amount of missing data, particularly the 90-day outcome assessment. After the patients are randomized, the patients or their families and doctors will keep WeChat (a Chinese social networking software similar to Facebook) and phone numbers for each other to avoid loss to follow-up.

9.2.2.1 Baseline covariates

Missing baseline covariates will be imputed using simple imputation methods in the covariate adjusted analysis based on the covariate distributions, should the missing values for a particular covariate be less than 5%. For a continuous variable, missing

values will be imputed from random values from a normal distribution with mean and SD calculated from the available sample. For a categorical variable, missing values will be imputed from random values from a uniform distribution with probabilities P_1, P_2, …, and P_k from the sample.

If the missing values for a covariate are ≥5% then they will be imputed using Markov chain Monte Carlo (MCMC) methods using SAS PROC MI. A total of 10 multiply imputed dataset will be generated. The seed for the imputations will be 128.

9.2.2.2 Efficacy outcomes

Sensitivity analyses based on different hypotheses about the missingness pattern of the primary outcome will be performed to test for the robustness of the primary analysis results[11]. Primary analysis of the primary outcome will be conducted in the complete case of the ITT population (in which patients with missing data will be censored). About other missing pattern please refer to Section 12.2.4.

9.3 Data Transformations

Data transformation is not relevant for most of the primary and secondary endpoints, which are categorical variables. The analysis of continuous variables may require transformation to normalize the distributions, but these variables will be mainly analysed using nonparametric statistical tests. Decisions to transform the distribution of some variables for analysis may be taken after the blind review of the data, and before unblinding the treatment code.

9.4 Multicenter Studies

No adjustment on centre will be performed for the primary analysis, on the basis of the following rationale:

The MARVEL trial aims to recruit 1672 patients from about 80 stroke centres in China, about 24 patients per centre. However, it is likely that many centres will recruit far

fewer patients, while a few large centres will recruit far more patients. This situation is the argument for not adjusted the primary analysis on centres. As the International Conference on Harmonization 9 guidelines quotes:

"In some trials, for example some large mortality trials with very few subjects per centre, there may be no reason to expect the centres to have any influence on the primary or secondary variables because they are unlikely to represent influences of clinical importance."

However, the central effect will be considered in the sensitivity analysis together with other important prognostic covariates.

9.5 Multiple Comparisons and Multiplicity

Analyses of secondary safety outcomes and additional analyses for the primary outcome are regarded as exploratory in nature, therefore, multiplicity adjustment will not apply to the primary and secondary outcome analyses.

9.6 Covariates

In the covariates-adjusted analysis, the following variables will be adjusted using the IPTW method.

- Age (Continuous)
- Baseline NIHSS score (Continuous)
- Pre-stroke modified Rankin Scale score (Ordered)
- Baseline aspects score (Continuous)
- Intravenous Thrombolysis (Binary)
- Time from stroke onset to randomization (Continuous)
- Occlusion location (Categorical)

The same IPTW weight will be used for all adjusted analyses.

9.7 Planned Subgroups

Subgroup analyses could be carried out irrespective of whether there is a significant treatment effect on the outcome. Their purpose is to supplement evidence from the primary analysis to help to fully characterize the treatment effect. Results from subgroup analyses will be interpreted in this context.

Subgroup analyses will be performed on a few important patient characteristics and other variables of interest. Pre-planned subgroup analyses will explore the effects of:

- Age (> median vs. ≤ median years old)
- Sex (Male vs. Female)
- Baseline NIHSS score (> median vs. ≤ median)
- Pre-stroke mRS (0 vs. 1 vs. 2 or more)
- Baseline ASPECTS score (> 5 vs. ≤ 5)
- Intravenous thrombolysis (yes vs. no)
- Time from last known well to randomization. (mins) (> median vs. ≤ median)
- Occlusion location (ICA vs. M1 vs. M2)
- Complete Reperfusion defined as Extended Thrombolysis In Cerebral Infarction grade 2c or 3 (yes or no)
- Patients with any radiological haemorrhages
- Patients with symptomatic haemorrhages

Subgroup analyses will be conducted irrespective of whether there is a significant treatment effect on the outcome. The purpose is to supplement evidence from the primary analysis to help to fully characterize the treatment effect. These analyses are exploratory and will not lead to formal inferential conclusions about the treatment effect in any particular subgroup.

A forest plot will be used to present the treatment effect of methylprednisolone for each of the individual subgroups. The plot will show the GenOR described above with two-

sided 95%CIs, corresponding to each level of these subgroups. Separately, the adjusted estimates for each of the patient characteristics defining a subgroup will be presented from the multivariate model.

Consistent with regulatory[12-14] to reduce the effect of the COVID-19 pandemic on the trial results, we prespecified the following subgroup analysis.

This subgroup included patients who completed 90-days follow up before 8th Jan 2023 when the Chinese Government officially downgraded the management of the disease from Class A to Class B in accordance with the country's law on prevention and treatment of infectious disease and remove it from quarantinable infectious disease management carried out in accordance with the Frontier Health and Quarantine Law of the People's Republic of China.

9.8 Derived and Computed Variables

Some derived and computed variables have been initially identified and described as appropriate later in the document. It is expected that additional variables will be required. The SAP will not be amended for additional variables that are not related to the primary target or key secondary target variables.

9.9 Presentation of results

Description of continuous variables includes the mean, standard deviation, median, first and third quartiles, and range. The description of categorical variables includes the frequency and percentage n (%). The description of ordered variables includes the frequency, percentage and cumulative percentage.

10. DISPOSITION OF SUBJECTS AND WITHDRAWALS

The number of patients for each of the following categories will be summarized.

- Total number of assessed patients
- Number of randomized patients

- Number of patients completing the study and not completing the study (grouped by treatment and main reason)
- Number of patients included in the ITT population
- Number of patients included in the PP population

11. DEMOGRAPHICS AND BASELINE CHARACTERISTICS

Clinically important demographic and disease characteristics at screening will be summarized with descriptive statistics for each treatment group. Summaries will be produced for the ITT population and PP population.

If the missing value exceeds 5%, the denominator will be added in the footnote of the corresponding summary table.

The variables to be summarized include but are not restricted to the following:

Age

Sex

Medical History (hypertension, coronary heart disease, smoking, diabetes mellitus, hyperlipidaemia, and prior stroke)

Pre stroke onset mRS score

Baseline NIHSS score

Baseline ASPECTS Score

Systolic and Diastolic Blood Pressure

Stroke Aetiology

Serum Glucose

Occlusion Sites

Intravenous Thrombolysis

American Society of Interventional and Therapeutic Neuroradiology/Society of Interventional Radiology (ASITN/SIR)

Time From Stroke Onset (Last Known Well) to Randomization.

Time From Stroke Onset (Last Known Well) to Puncture

Time From Puncture to Reperfusion or the End of the Operation

Time From Randomization to Initiation of study drugs.

12. EFFICACY ANALYSES

All efficacy analyses will provide the point estimate of treatment effect with associated two-sided 95% confidence intervals (CIs). Statistical analyses of the primary and secondary efficacy endpoints will primarily be based on the ITT population. Secondarily, the same analyses will be repeated on the PP population.

Assessment of efficacy will be conducted based on endpoints collected during the Day 90 follow-up. The protocol allows this visit to be scheduled within 7 days of the target 90 days after randomization.

12.1 Primary Efficacy Endpoint (Ordinal data)

The mRS score is an assessment of disability with values from 0 (no symptoms at all) to 5 (sever disability) to 6 (death). The mRS score is collected at 90 days after randomization. If death occurs prior to an assessment day (Day 90), the mRS score will be considered available and will be set to 6.

For the analysis of the ordinal outcomes, Agresti proposed a generalized odds ratio (GenOR) for evaluating the difference between two groups.[15] Agresti's generalised odds ratio was defined as the ratio between the proportion of all pairs with better outcomes in the treatment group and the proportion of all pairs with better outcomes in the control group.[16] GenOR can be calculated using the following formula:

$$GenOR = \frac{P(Y_T < Y_C)}{P(Y_T > Y_C)}$$

Where Y_T and Y_C are the mRS scores in the treatment arm and control arm, respectively. $P(Y_T < Y_C)$ is the probability that among all possible pairwise comparisons between treatment and control a subject in treatment arm has a smaller mRS score than that in control arm, and $P(Y_T > Y_C)$ is the probability that among all possible pairwise comparisons between treatment and control a subject in treatment arm has a larger mRS score than that in control arm. [13]

The GenOR is actually identical to the win ratio statistic proposed by Pocock et al in 2012.[15,17] We will therefore use the WINS package in R to perform GenOR analysis, in which the variance of win ratio (GenOR) is estimated using a method proposed by Dong et al.[18] In case of binary outcome, genearalized odds ratio is actually the conventional odds ratio. Since the generalised odds ratio was proposed in 1980 by Agresti)[15] well before win ratio statistic proposed in 2012 by Pocock et al[17], we will use the term generalised odds ratio or genOR in the statistical reporting.

The distribution of full range of primary endpoint, mRS scores at Day 90, will be summarized by treatment group using a summary table and stacked bar chart. The primary endpoint will also be descriptively summarised using proportion of wins in treatment arm over control arm ($P(Y_T < Y_C)$ and proportion of wins in control arm over treatment arm ($P(Y_T > Y_C)$. The treatment effect will be measured using genOR together with its 95%CI and P-value.

The primary analysis of the primary endpoint will be conducted based on the complete case of the ITT population (patients with missing outcome data will be excluded) Sensitivity analysis based on different missing pattern and statistical method will be employed to test to robustness of the results.

12.1.1 Crude analysis

The null and alternative hypotheses to be tested appear below:

The null hypothesis for the generalized OR (GenOR) is equal to one:

$$GenOR = 1$$

The above hypothesis can be rephrased as the probability Prob ($Y_T < Y_C$) is equal to the probability Prob ($Y_T > Y_C$). Alternative hypothesis is

$GenOR \neq 1$. The point estimate and 95%CI of the crude GenOR will be estimated using the method by Dong et al[18] using the WINS package in R. Null hypothesis will be rejected if the 95%CI for the GenOR does not include 1.

12.1.2 Covariates adjusted analysis

The covariates adjusted generalized odds ratio will be calculated using the IPTW combined using the WINS package.

We will first calculate a propensity score with treatment as the dependent variable (1 for active treatment group and 0 for control) and all covariates listed above as independent variables through a logistic regression model, and then calculate the inverse probability of treatment weighting (IPTW) for each subject. In the next step, we will calculate the adjusted generalized odds ratio with the IPTW method. The application of these weights to the study population creates a pseudopopulation in which confounders are equally distributed across treatment and control groups.

12.1.3 Sensitivity analysis

To assess the influence of the missing primary endpoints on the treatment effect estimate, sensitivity analyses will be performed using the same statistical methods as described in Section 12.1.1 and 12.1.2, under the different assumptions of the missing outcome below:

(1) Worst-case scenario:

All patients with a missing primary endpoint will be considered as having the worst mRS score of 6.

(2) Best-case scenario:

All patients with a missing primary endpoint will be considered as having the best mRS

score of 0.

(3) Multiple Imputation

Missing mRS score will be imputed using multiple imputation method via SAS PROC MI. Imputation procedure will be performed under the missing-at-random (MAR) assumption. Fully conditional method (FCS) will be used to predict missing mRS score by fitting an ordered logistic regression model with following predictors: treatment group, age, baseline NIHSS score, pre-stroke mRS, baseline ASPECTS score, use of intravenous thrombolysis, time from onset to randomization, and occlusion location, and last observed NIHSS if available.[19] The seed will be 128. A total of 10 imputed datasets will be generated. GenOR will be estimated for each dataset separately, and the pooled together to calculate the final pooled genOR and 95% confidence interval using the Robin's role.

The above analysis will be performed on the imputed primary outcome for both ITT and PP populations as described in Section 12.1.2.

12.1.4 Supplementary analysis

To access the robustness of the primary analysis, the following supportive analysis will be employed as complement to the primary analysis. The analysis will be conducted in the complete case population of the primary outcomes.

(1) Proportional odds model. A proportional odds model will be employed in the sensitivity analysis. Both the unadjusted and adjusted results will be presented. The adjusted model will include age, baseline NIHSS score, pre-stroke mRS, baseline ASPECTS score, the use of intravenous thrombolysis, time from onset to randomization, and occlusion location as covariates.

(2) Patial Proportional Odds Model. If the proportional odds assumption holds true for unadjusted analysis in the proportional odds model, but fails when adjusted for age, baseline NIHSS score, pre-stroke mRS, baseline ASPECTS score, the use of intravenous thrombolysis, time from onset to randomization, and occlusion location,

then a partial proportional odds model will be fitted to estimate the common odds ratio of treatment.[20,21]

To access the influence of centre on the treatment effect, the following methods will be used:

(3) Proportional odds model with random effect: A proportional odds model with random effect will be fitted to estimate the common odds ratio between the methylprednisolone sodium succinate and placebo treatment with treatment as fixed effect, age, baseline NIHSS score, pre-stroke mRS, baseline ASPECTS score, use of intravenous thrombolysis, time from onset to randomization, and occlusion location as covariates, and the centre as a random effect.

12.1.5 Subgroup analysis

Subgroup analysis of the primary endpoint will be performed on the following subgroup variables, and the GenOR and its 95% CI will be estimated for each category of a subgroup variable and displayed in a forest plot to assess exploratorily homogeneity of treatment effect by a subgroup variable.

- age (> median vs. ≤ median years old)
- sex (Male vs. Female)
- baseline NIHSS score (> median vs. ≤ median)
- pre-stroke mRS (0 vs. 1 vs. 2 or more)
- baseline ASPECTS score (> 5 vs. ≤ 5)
- intravenous thrombolysis (yes vs. no)
- time from last known well to randomization. (mins) (> median vs. ≤ median)
- occlusion location (ICA vs. M1 vs. M2)
- Complete Reperfusion defined as Extended Thrombolysis in Cerebral Infarction grade 2c or 3 (yes or no)

- Patients with any radiological haemorrhages (with vs without)
- Patients with symptomatic haemorrhages (with vs without)

Subgroup analysis will be performed on both ITT and PP populations.

12.2 Secondary Efficacy Analysis

12.2.1 Dichotomized modified Rankin Scale score. (Binary data)

The treatment effect for binary secondary outcomes will be estimated in the ITT population using a modified Poisson regression as the risk ratio (RR) and its 95%CI.[22]

In the adjusted analysis, IPTW method will be used: we will first calculate a propensity score with treatment as the dependent variable (1 for active treatment group and 0 for control) and all covariates listed below as independent variables through a logistic regression model, and then perform an inverse probability of treatment weighting (IPTW) analysis and calculate the Adjusted RR with 95% CI.[23,24] Covariates including age, baseline NIHSS score, pre-stroke mRS, baseline ASPECTS score, use of intravenous thrombolysis, time from onset to randomization, and occlusion location to randomization.

In addition, RD (risk difference) and OR will be estimated and presented using two GLMs (identity-binomial regression and logit-binomial regression) as supportive measurements of treatment effect.[25] IPTW method will be calculate the covariate adjusted RD and OR.

12.2.2 NIHSS score at 5-7 days or at early discharge (Continuous data)

The NIHSS is a standardized neurological examination score, with scores ranging from 0 to 42, with higher scores indicating increasing severity.

The mean difference and 95%CI of NIHSS score between two groups will be estimated using a GLM with treatment group as the sole predictor and the NIHSS score at 5-7 days or discharge as the dependent variable. Subjects with missing NIHSS score at 5-7

days or discharge will be excluded from the analysis. IPTW method will be employed in calculating the co-variates adjusted mean difference (Co-variates will be defined as Section 9.6). Normality and variance homogeneity of residuals will be accessed graphically. The win ratio method will be used if normality assumption is seriously violated.

12.2.3 European Quality Five-Dimension (EQ-5D) (Continuous data)

The EQ-5D is a tool for describing and assessing health-related quality of life 90±7 days after randomization. The tool is designed for self-completion.-The EQ-5D will be completed on the 90 (±7) days after randomization. Reasons for not performing the assessment of EQ-5D at Day 90 will be summarized.

The mean difference and 95%CI of EQ-5D score between two groups will be estimated using a GLM with treatment group as the sole predictor and the 90 days EQ-5D score as the dependent variable. IPTW method will be employed in calculating the co-variates adjusted mean difference and corresponding 95%CI (Co-variates will be defined as Section 9.6).

Subjects with missing EQ-5D score will be excluded from the analysis. Normality and variance homogeneity of residuals will be accessed graphically. The win ratio method will be used if normality assumption is seriously violated.

13. SAFETY ANALYSES

Safety analyses will be conducted on the Safety population only.

13.1 Mortality at 90 days (Binary data)

13.1.1 Defined of endpoint

Mortality due to any cause is the primary safety endpoint. The outcome is measured on a binary scale and is defined as positive in the presence of death of any cause, and as

negative otherwise. With the date of randomization at day 0, any death occurring on or before calendar day 90 will be counted as a death. Patients who are alive at day 90 will be censored at day 90. Mortality rates are defined as the number of deaths observed divided by the number of subjects observed over the 90-day study period.

13.1.2 Statistical Analysis

The modified Poisson regression with a treatment group as an independent variable and the cumulative rate death due to any cause at 90 days as the dependent variable. The treatment effect will be presented as RR with the corresponding 95%CI. IPTW will be used in calculating the adjusted RR and its 95%CI.

In addition, proportional hazards regression model will be employed to calculate the hazard ratio with 95%CI. IPTW method will be used to calculate the adjusted hazard ratio. In addition, the Kaplan-Meier method will be used to display the survival curves by treatment arm. The log-rank test will be applied to compare survival curves between the two treatment groups.

13.2 SICH rate within 48 hours (Binary data)

13.2.1 Defined of endpoint

Proportion of patients with symptomatic intracranial haemorrhage (SICH) within 48 hours post-treatment is considered another primary safety outcome. SICH will be evaluated according to the Heidelberg Bleeding Classification[26].

13.2.2 Statistical Analysis

Percentage of subjects with SICH within 48 hours after treatment will be presented for each definition by treatment group. Frequency counts and percentage of patients within each category will be provided for categorical data. Subject rates will be compared between treatment groups with Chi-square test or Fisher's exact test.

The modified Poisson Regression model will be used to estimates the RR and corresponding 95%CI with treatment group as an independent variable and the presence or absence of SICH as the dependent variable. The treatment effect will be presented as RR with the corresponding 95%CI. IPTW methods will be employed to calculate the adjusted RR and 95%CI.

13.3 Any ICH within 48 hours (Binary data)

The statistical analysis of differences in proportions of patients with any radiologic ICH within 48 hours between methylprednisolone sodium succinate and placebo groups will be performed as per the Section 13.2. SICH rate within 48 hours.

13.4 Proportion of patients with pneumonia (Binary data)

Number of patients and percentage of subjects with pulmonary infection within 48 hours after treatment will be presented by treatment group. The between group difference will be tested using the modified Poisson Model. And the Adjusted RR and corresponding 95% CI will be estimated using the IPTW method as other binary outcomes. Both unadjusted and adjusted results will be presented.

13.5 Proportion of patients with gastrointestinal bleeding within 7 days after EVT (Binary data)

Number of patients and percentage of subjects' gastrointestinal bleeding within 7 days after EVT will be presented by treatment group. The between group difference will be

tested using the modified Poisson Model. The Adjusted RR and corresponding 95%CI will be estimated using the IPTW method as other binary outcomes. Both unadjusted and adjusted results will be presented.

13.6. ADVERSE EVENTS

Adverse events (AEs) and diseases will recorded in this study.

13.6.1 Adverse Events

Any adverse change in health or the appearance of or worsening of any undesirable sign, symptom, or medical condition occurring after enrollment into the trial will be recorded as Adverse Event (AE) whether or not it is considered to be related to the study drug. An AE also includes a new illness; aggravated in severity of frequency from the baseline condition, abnormal results of diagnostic procedures, or a combination of the above. Pre-existing medical conditions are not to be reported as AEs.

13.6.2 Serious Adverse Events

A serious adverse event (SAE) is any untoward medical occurrence that at any dose: Result in death; Are life-threatening or fatal; Require or prolong hospitalization; Result in persistent or significant disability/incapacity, Constitutes a congenital anomaly or birth defect, or Significant medical event.

A SAE can also be an important medical event that may not result in death, be life-threatening, or require hospitalization, but may jeopardize the subject and may require medical or surgical intervention to prevent one of the outcomes listed in this definition. For example, any new diagnosis of cancer (made after study enrollment) is considered an important medical event. Because our primary safety outcomes for the trial are also SAEs, they will be reported dually as SAEs and as outcomes. SAEs should be managed according to the best current standard of care. All deaths occurring during the follow-up to Day 90 will be reported as an SAE. When reporting a death, the event or condition that caused or contributed to the fatal outcome should be reported as a single medical

concept.

References

1. CPMP/ICH/363/96. ICH E9 Statistical Principles for Clinical Trials.

2. Association AS. Ethical Guidelines for Statistical Practice. Prepared by the Committee on Professional Ethics.

3. Society RS. The Royal Statistical Society: Code of Conduct.

4. Zi W, Wang H, Yang D, et al. Clinical Effectiveness and Safety Outcomes of Endovascular Treatment for Acute Anterior Circulation Ischemic Stroke in China. Cerebrovasc Dis 2017;44:248-58.

5. Goyal M, Menon BK, van Zwam WH, et al. Endovascular thrombectomy after large-vessel ischaemic stroke: a meta-analysis of individual patient data from five randomised trials. LANCET 2016;387:1723-31.

6. Cranston JS, Kaplan BD, Saver JL. Minimal Clinically Important Difference for Safe and Simple Novel Acute Ischemic Stroke Therapies. Stroke 2017;48:2946-51.

7. Berkhemer OA, Fransen PSS, Beumer D, et al. A Randomized Trial of Intraarterial Treatment for Acute Ischemic Stroke. New England Journal of Medicine 2015;372:11-20.

8. White IR, Horton NJ, Carpenter J, Pocock SJ. Strategy for intention to treat analysis in randomised trials with missing outcome data. Bmj 2011;342:d40.

9. SAS version 9.4 software, SAS Institute Inc., Cary, NC,.

10. Rev1. CE. Guideline on Missing Data in confirmatory trials.

11. (EMA/CHMP/ICH/436221/2017). ICH E9 (R1) addendum on estimands and sensitivity analysis in clinical trials to the guideline on statistical principles for clinical trials. Draft.

12. Administration. UFaD. Conduct of clinical trials of medical products during the COVID-19 public health emergency guidance for industry, investigators, and institutional review boards. March, 2020, updated on Aug, 30, 2021. https://www.fda.gov/ media/136238/download (accessed Oct 8, 2022).

13. Agency. EM. Guidance on the management of clinical trials during the COVID-19 (coronavirus) pandemic. Version 5. https://health.ec.europa.eu/system/files/2022-02/ guidanceclinicaltrials_covid19_en_1.pdf (accessed Oct 8, 2022).

14. 国家药品监督管理局. NMPA. Guiding Principles for the Management of Drug Clinical Trials During the COVID-19 Pandemic.《新冠肺炎疫情期间药物临床试验管理指导原则（试行)》.

15. A. A. Generalized odds ratios for ordinal data. . Biometrics 1980;36:59–67.

16. Churilov L, Arnup S, Johns H, et al. An improved method for simple, assumption-free ordinal analysis of the modified Rankin Scale using generalized odds ratios. International Journal of Stroke 2014;9:999-1005.

17. Pocock SJ, Ariti CA, Collier TJ, Wang D. The win ratio: a new approach to the analysis of composite endpoints in clinical trials based on clinical priorities. Eur Heart J 2012;33:176-82.

18. Dong G, Li D, Ballerstedt S, Vandemeulebroecke M. A generalized analytic solution to the win ratio to analyze a composite endpoint considering the clinical importance order among components. Pharm Stat 2016;15:430-7.

19. Young-Saver DF, Gornbein J, Starkman S, Saver JL. Handling of Missing Outcome Data in Acute Stroke Trials: Advantages of Multiple Imputation Using Baseline and Postbaseline Variables. J Stroke Cerebrovasc Dis 2018;27:3662-9.

20. Schildcrout JS, Harrell FE, Jr., Heagerty PJ, et al. Model-assisted analyses of longitudinal, ordinal outcomes with absorbing states. Stat Med 2022;41:2497-512.

21. Desantis SM, Lazaridis C, Palesch Y, Ramakrishnan V. Regression analysis of ordinal stroke clinical trial outcomes: an application to the NINDS t-PA trial. Int J Stroke 2014;9:226-31.

22. Zou G. A modified poisson regression approach to prospective studies with binary data. Am J Epidemiol 2004;159:702-6.

23. Austin PC, Stuart EA. Moving towards best practice when using inverse probability of treatment weighting

(IPTW) using the propensity score to estimate causal treatment effects in observational studies. Stat Med 2015;34:3661-79.
24. Morris TP, Walker AS, Williamson EJ, White IR. Planning a method for covariate adjustment in individually randomised trials: a practical guide. Trials 2022;23:328.
25. Naimi AI, Whitcomb BW. Estimating Risk Ratios and Risk Differences Using Regression. Am J Epidemiol 2020;189:508-10.
26. von Kummer R, Broderick JP, Campbell BCV, et al. The Heidelberg Bleeding Classification Classification of Bleeding Events After Ischemic Stroke and Reperfusion Therapy. Stroke 2015;46:2981-6.
27. National Institute of Neurological D, Stroke rt PAS, Study G. Tissue plasminogen activator for acute ischemic stroke. The New England journal of medicine 1995;333:1581-7.
28. Hacke W, Kaste M, Fieschi C, et al. Randomised double-blind placebo-controlled trial of thrombolytic therapy with intravenous alteplase in acute ischaemic stroke (ECASS II). Second European-Australasian Acute Stroke Study Investigators. Lancet (London, England) 1998;352:1245-51.
29. Hacke W, Kaste M, Bluhmki E, et al. Thrombolysis with alteplase 3 to 4.5 hours after acute ischemic stroke. N Engl J Med 2008;359:1317-29.
30. Wahlgren N, Ahmed N, Eriksson N, et al. Multivariable analysis of outcome predictors and adjustment of main outcome results to baseline data profile in randomized controlled trials: Safe Implementation of Thrombolysis in Stroke-MOnitoring STudy (SITS-MOST). Stroke 2008;39:3316-22.

Summary of Changes - Statistical Analysis Plan MARVEL Version 1.0 to Version 2.0

Below is the table of changes. Deleted items are identified with ~~Strikethrough~~ font. Additional wording is in ***bold, italics, and underlining*** font

Section(s)	SAP Version 1.0<br>Change From:	SAP Version 2.0<br>Change To:	Rationale
5.2.1 Inclusion Criteria	Baseline Alberta Stroke Program Early CT Score (ASPECTS)>= 5;	***Added:***<br>Baseline Alberta Stroke Program Early CT Score (ASPECTS)>= ~~5~~***3***;	Addition<br>To be consistent with the Change of Inclusion Criteria of Protocol.
9.1 Analysis Software	All statistical analyses will be performed using SAS® Software version 9.4 in a Windows environment9 and R version 4.1.1.	All statistical analyses will be performed using SAS® Software version 9.4 in a Windows environment9 and R version ~~4.1.1.~~***4.3.0.***	Update the R version
9.2.2.2 Efficacy outcomes		***Added:***<br>About other missing pattern please refer to Section 12.2.4.	Added to refer other sensitivity analysis under different missing assumption.
Section 9.6 Planned Subgroups, Interactions, and Covariates	Age<br>Baseline NIHSS score<br>Pre-stroke modified Rankin Scale score<br>Baseline aspects score<br>Intravenous Thrombolysis<br>Time from stroke onset to randomization<br>Occlusion location (Categorical)	***Changed:***<br>Age ***(Continuous)***<br>Baseline NIHSS score ***(Continuous)***<br>Pre-stroke modified Rankin Scale score ***(Ordered)***<br>Baseline aspects score ***(Continuous)***<br>Intravenous Thrombolysis ***(Binary)***<br>Time from stroke onset to randomization ***(Continuous)***<br>Occlusion location ***(Categorical)***	Addition<br>To make the covariates more clear.

9.7 Planned Subgroups	A forest plot will be used to present the treatment effect of methylprednisolone for each of the individual subgroups. The plot will show the cOR described above with two-sided 95%CIs, corresponding to each level of these subgroups. Separately, the adjusted estimates for each of the patient characteristics defining a subgroup will be presented from the multivariate model.	A forest plot will be used to present the treatment effect of methylprednisolone for each of the individual subgroups. The plot will show the ~~cOR~~***GenOR*** described above with two-sided 95%CIs, corresponding to each level of these subgroups. Separately, the adjusted estimates for each of the patient characteristics defining a subgroup will be presented from the multivariate model.	Addition<br>To be consistent with the Change of analysis method of the primary analysis.
9.7 Planned Subgroups		***Consistent with regulatory12-14 to reduce the effect of the COVID-19 pandemic on the trial results, we prespecified the following subgroup analysis.***<br>***This subgroup included patients who completed 90-days follow up before 8th Jan 2023 when the Chinese Government officially downgraded the management of the disease from Class A to Class B in accordance with the country's law on prevention and treatment of infectious disease and remove it from quarantinable infectious disease management carried out in accordance with the Frontier Health and Quarantine Law of the People's Republic of China.***	As COVID-19 is an important variable that will influence the effect of corticosteroid, a sensitivity analysis was determined by the steering committee.
12.1 Primary Efficacy Endpoint	12.1 Primary Efficacy Endpoint	12.1 Primary Efficacy Endpoint **(*Ordinal data*)**	Addition,<br>To make the definition

			more clear.
12.1 Primary Efficacy Endpoint (Ordinal data)	As the primary endpoint, mRS score at Day 90 will be further explored on the range of the values (0 to 6). The distribution of full range of mRS scores at Day 90 will be summarized by treatment group. Both tabular displays and stacked bar charts will be prepared. Win ratio method will be used to calculate the win ratio statistic as a measurement of treatment effect in mRS score.12 Primary analysis of the primary outcome will be conducted in the complete case of the ITT population (in which patients with missing data will be deleted) using the proportional odds model.<br>12.1.1 Hypothesis<br>The null and alternative hypotheses to be tested appear below:<br>The null hypothesis for proportional odds logistic regression is that the common OR is equal to one, which needs to be disproved. The null hypothesis for WMW test is the equality of ranks when ties are split evenly. This can be rephrased as both probabilities Prob (YM > YP) + 0.5Prob (YM = YP)	~~As the primary endpoint, mRS score at Day 90 will be further explored on the range of the values (0 to 6). The distribution of full range of mRS scores at Day 90 will be summarized by treatment group. Both tabular displays and stacked bar charts will be prepared. Win ratio method will be used to calculate the win ratio statistic as a measurement of treatment effect in mRS score.12 Primary analysis of the primary outcome will be conducted in the complete case of the ITT population (in which patients with missing data will be deleted) using the proportional odds model.~~<br>~~12.1.1 Hypothesis~~<br>~~The null and alternative hypotheses to be tested appear below:~~<br>~~The null hypothesis for proportional odds logistic regression is that the common OR is equal to one, which needs to be disproved. The null hypothesis for WMW test is the equality of ranks when ties are split evenly. This can be rephrased as both probabilities Prob (YM > YP) + 0.5Prob (YM = YP) and Prob (YM < YP) + 0.5Prob (YM = YP) being equal to 0.5, resulting in WMW GenOR being equal to 1. Therefore, the null hypothesis for the WMW test states that there's an equal probability of treatment observation being worse or better than control observation (with equally splitting ties),~~	Changed the primary outcome analysis. The initial statistical method was the proportional odds regression adjusted for covariates using the Inverse probability treatment weighting method.<br>The analysis method was change with the concern that the proportional odds model assumption can rarely held after adjusting for covariates.<br>An assumption-free method was considered to be the primary analysis consisted with two previous studies published in the New England journal of medicine. (the ANGEL-ASPECTS study and the SELECT2 study). And the proportion odds assumption was

	and Prob (YM < YP) + 0.5Prob (YM = YP) being equal to 0.5, resulting in WMW GenOR being equal to 1. Therefore, the null hypothesis for the WMW test states that there's an equal probability of treatment observation being worse or better than control observation (with equally splitting ties), meaning that WMW GenOR isn't different from one.	~~meaning that WMW GenOR isn't different from one.~~<br>***For the analysis of the ordinal outcomes, Agresti proposed a generalized odds ratio (GenOR) for evaluating the difference between two groups.15Agresti's generalised odds ratio was defined as the ratio between the proportion of all pairs with better outcomes in the treatment group and the proportion of all pairs with better outcomes in the control group.16GenOR can be calculated using the following formula:***<br>$GenOR=(P(Y_T<Y_C))/(P(Y_T>Y_C))$<br>***Where YT and YC are the mRS scores in the treatment arm and control arm, respectively.*** $P(Y_T<Y_C)$ ***is the probability that among all possible pairwise comparisons between treatment and control a subject in treatment arm has a smaller mRS score than that in control arm, and*** $P(Y_T>Y_C)$ ***is the probability that among all possible pairwise comparisons between treatment and control a subject in treatment arm has a larger mRS score than that in control arm. 13***<br>***The GenOR is actually identical to the win ratio statistic proposed by Pocock et al in 2012.15,17 We will therefore use the WINS package in R to perform GenOR analysis, in which the variance of win ratio (GenOR) is estimated using a method proposed by Dong et al.18 In case of***	considered as an supportive analysis.

		binary outcome, genearalized odds ratio is actually the conventional odds ratio. Since the generalised odds ratio was proposed in 1980 by Agresti)15 well before win ratio statistic proposed in 2012 by Pocock et al17, we will use the term generalised odds ratio or genOR in the statistical reporting.<br>***The distribution of full range of primary endpoint, mRS scores at Day 90, will be summarized by treatment group using a summary table and stacked bar chart. The primary endpoint will also be descriptively summarised using proportion of wins in treatment arm over control arm ($P(Y_T<Y_C)$) and proportion of wins in control arm over treatment arm ($P(Y_T>Y_C)$). The treatment effect will be measured using genOR together with its 95%CI and P-value.***<br>***The primary analysis of the primary endpoint will be conducted based on the complete case of the ITT population (patients with missing outcome data will be excluded) Sensitivity analysis based on different missing pattern and statistical method will be employed to test to robustness of the results.***	
12.1.1 Crude analysis	The categorical shift in the distribution of mRS scores between the two	~~The categorical shift in the distribution of mRS scores between the two treatment groups will be~~	

	treatment groups will be analysed fitting a proportional-odds logistic regression model, assuming a common odds ratio across all cut points of the modified Rankin scale.<br>a) If the proportional odds assumptions are satisfied (both Brant test and Approximate likelihood-ratio test of proportionality of odds are not significant), common OR and 95%CI will be calculate from an GLM with multinomial distribution and cumlogit-link(ordinal logistic regression model) 13,14;<br>b) If the proportional odds assumptions are not satisfied for the crude analysis (significant Brant test or Approximate likelihood-ratio test of proportionality of odds), assumption-free method will be used.15	~~analysed fitting a proportional-odds logistic regression model, assuming a common odds ratio across all cut points of the modified Rankin scale.~~<br>~~a) If the proportional odds assumptions are satisfied (both Brant test and Approximate likelihood-ratio test of proportionality of odds are not significant), common OR and 95%CI will be calculate from an GLM with multinomial distribution and cumlogit-link(ordinal logistic regression model) 13,14;~~<br>~~b) If the proportional odds assumptions are not satisfied for the crude analysis (significant Brant test or Approximate likelihood-ratio test of proportionality of odds), assumption-free method will be used.15~~ ***The null and alternative hypotheses to be tested appear below:***<br>***The null hypothesis for the generalized OR (GenOR) is equal to one:***<br>***GenOR=1***<br>***The above hypothesis can be rephrased as the probability Prob (YT < YC) is equal to the probability Prob (YT > YC). Alternative hypothesis is***<br>***GenOR≠1 . The point estimate and 95%CI of the crude GenOR will be estimated using the method by Dong et al18 using the WINS package in R. Null hypothesis will be rejected if the 95%CI for the GenOR does not include 1.***	

12.1.2.2 Covariates Adjusted Analysis	a) If the proportional odds assumptions are satisfied for crude analysis, and still satisfied when adding the covariates in the ordinal logistic regression model, then the common odds ratio from an ordinal logistic regression model, IPTW method will be employed in the co-variate adjusted analysis. 13,14,16<br>b) If the proportional odds assumptions are not satisfied for the IPTW adjusted analysis (significant Brant test or Approximate likelihood-ratio test of proportionality of odds), assumption-free method will be used.15<br>Covariate adjusted analysis will be performed on both ITT and PP populations.<br>Main conclusion will be drawn from the adjusted ITT analysis of the primary endpoint.	~~a) If the proportional odds assumptions are satisfied for crude analysis, and still satisfied when adding the covariates in the ordinal logistic regression model, then the common odds ratio from an ordinal logistic regression model, IPTW method will be employed in the co-variate adjusted analysis. 13,14,16~~<br>~~b) If the proportional odds assumptions are not satisfied for the IPTW adjusted analysis (significant Brant test or Approximate likelihood-ratio test of proportionality of odds), assumption-free method will be used.15~~<br>~~Covariate adjusted analysis will be performed on both ITT and PP populations.~~<br>~~Main conclusion will be drawn from the adjusted ITT analysis of the primary endpoint.~~***12.1.3 Covariates Adjusted Analysis***<br>***a) If the proportional odds assumptions are satisfied for crude analysis, and still satisfied when adding the covariates in the ordinal logistic regression model, then the common odds ratio from an ordinal logistic regression model, IPTW method will be employed in the co-variate adjusted analysis. 13,14,16***<br>***b) If the proportional odds assumptions are not satisfied for the crude analysis (significant Brant test or Approximate likelihood-ratio test of proportionality of odds), assumption-free method will be used.15***	

		<u>Covariate adjusted analysis will be performed on both ITT and PP populations.</u><br>***<u>Main conclusion will be drawn from the adjusted ITT analysis of the primary endpoint.</u>***	
12.1.4 Sensitivity analysis	(3) Multiple Imputation<br>Missing mRS score will be imputed using multiple imputation method via SAS PROC MI. Imputation procedure will be performed under the missing-at-random (MAR) assumption. Fully conditional method (FCS) will be used to predict missing mRS score by fitting an ordered logistic regression model with following predictors: treatment group, age, baseline NIHSS score, pre-stroke mRS, baseline ASPECTS score, use of intravenous thrombolysis, time from onset to randomization, and occlusion location, and last observed NIHSS if available.[19] The seed will be 128. A total of 10 imputed datasets will be generated. cOR will be estimated for each dataset separately, and the pooled together to calculate the final pooled cOR and 95% confidence interval using the Robin's role.	Missing mRS score will be imputed using multiple imputation method via SAS PROC MI. Imputation procedure will be performed under the missing-at-random (MAR) assumption. Fully conditional method (FCS) will be used to predict missing mRS score by fitting an ordered logistic regression model with following predictors: treatment group, age, baseline NIHSS score, pre-stroke mRS, baseline ASPECTS score, use of intravenous thrombolysis, time from onset to randomization, and occlusion location, and last observed NIHSS if available.19 The seed will be 128. A total of 10 imputed datasets will be generated. ~~cOR~~***<u>GenOR</u>*** will be estimated for each dataset separately, and the pooled together to calculate the final pooled ~~cOR~~***<u>genOR</u>*** and 95% confidence interval using the Robin's role.	Change from cOR to genOR to be consistent to the change of the primary outcome.
12.1.4 Supplementary		***<u>Added: To access the robustness of the primary</u>***	Added the cOR method

analysis		***analysis, the following supportive analysis will be employed as complement to the primary analysis. The analysis will be conducted in the complete case population of the primary outcomes.***<br><br>***(2) Proportional odds model. A proportional odds model will be employed in the sensitivity analysis. Both the unadjusted and adjusted results will be presented. The adjusted model will include age, baseline NIHSS score, pre-stroke mRS, baseline ASPECTS score, the use of intravenous thrombolysis, time from onset to randomization, and occlusion location as covariates.***<br><br>***(2) Patial Proportional Odds Model. If the proportional odds assumption holds true for unadjusted analysis in the proportional odds model, but fails when adjusted for age, baseline NIHSS score, pre-stroke mRS, baseline ASPECTS score, the use of intravenous thrombolysis, time from onset to randomization, and occlusion location, then a partial proportional odds model will be fitted to estimate the common odds ratio of treatment.20,21***<br><br>***To access the influence of centre on the***	as supportive analysis method in consist to the change of the primary analysis method.

		treatment effect, the following methods will be used:<br>***(4) Proportional odds model with random effect: A proportional odds model with random effect will be fitted to estimate the common odds ratio between the methylprednisolone sodium succinate and placebo treatment with treatment as fixed effect, age, baseline NIHSS score, pre-stroke mRS, baseline ASPECTS score, use of intravenous thrombolysis, time from onset to randomization, and occlusion location as covariates, and the centre as a random effect.***	
12.1.5 Subgroup analysis	Assessment of the homogeneity of treatment effect by a subgroup variable will be conducted by a GLM with the treatment, subgroup variable, and their interaction term as predictors, adjusted for age, baseline NIHSS score, pre-stroke mRS, baseline ASPECTS score, use of intravenous thrombolysis, time from onset to randomization, and occlusion location, and the P-value from the interaction term will be used to assess exploratorily homogeneity of treatment effect by a subgroup	~~Assessment of the homogeneity of treatment effect by a subgroup variable will be conducted by a GLM with the treatment, subgroup variable, and their interaction term as predictors, adjusted for age, baseline NIHSS score, pre-stroke mRS, baseline ASPECTS score, use of intravenous thrombolysis, time from onset to randomization, and occlusion location, and the P-value from the interaction term will be used to assess exploratorily homogeneity of treatment effect by a subgroup variable.~~<br>***Subgroup analysis of the primary endpoint will be performed on the following subgroup***	Rephrase the sentence, to be consistent with the change of primary outcome. And currently there was no robust method to calculate formal p value for interaction for the generalized odds ratio,

	variable.	***variables, and the GenOR and its 95% CI will be estimated for each category of a subgroup variable and displayed in a forest plot to assess exploratorily homogeneity of treatment effect by a subgroup variable.***	
12.2.1 Dichotomized modified Rankin Scale score. (Binary data)	12.2.1 Dichotomized modified Rankin Scale score.	12.2.1 Dichotomized modified Rankin Scale score. ***(Binary data)***	Added the variable type to define the variable more clearly.
12.2.2 NIHSS score at 5-7 days or at early discharge	12.2.2 NIHSS score at 5-7 days or at early discharge	12.2.2 NIHSS score at 5-7 days or at early discharge ***(Continuous data)***	Added the variable type to define the variable more clearly.
12.2.3 European Quality Five-Dimension (EQ-5D) (Continuous data)	12.2.3 European Quality Five-Dimension (EQ-5D)	12.2.3 European Quality Five-Dimension (EQ-5D) ***(Continuous data)***	Added the variable type to define the variable more clearly.
13.1 Mortality at 90 days (Binary data)	13.1 Mortality at 90 days	13.1 Mortality at 90 days ***(Binary data)***	Added the variable type to define the variable more clearly.
13.2 SICH rate within 48 hours (Binary data)	13.2 SICH rate within 48 hours	13.2 SICH rate within 48 hours ***(Binary data)***	Added the variable type to define the variable more clearly.
