# Supplementary material for: Methylprednisolone as Adjunct to Thrombectomy for Acute Intracranial Internal Carotid Artery Occlusion Stroke: Post Hoc Secondary Analysis of the MARVEL Randomized Clinical Trial
Source: JAMA Netw Open. 2025 Feb 18;8(2):e2459945. doi: 10.1001/jamanetworkopen.2024.59945 (PMC11836765; doi:10.1001/jamanetworkopen.2024.59945)
Supplement: Supplement 2. — eMethods. Inverse Probability of Treatment Weighting eTable 1. Heterogeneity of the Treatment Effect of Methylprednisolone vs Placebo in Patients With or Without Intracranial Carotid Occlusion eTable 2. Efficacy and Safety Outcomes of Intravenous Methylprednisolone vs Placebo Before Endovascular Thrombectomy of Patients With Intracranial Internal Carotid Artery Occlusion [file jamanetwopen-e2459945-s002.pdf]

Supplementary Online Content

Zheng C, Li R, Shen C, et al. Methylprednisolone as adjunct to thrombectomy for acute intracranial internal carotid artery occlusion stroke: post hoc secondary analysis of the MARVEL randomized clinical trial. *JAMA Netw Open.* 2025;8(2):e2459945. doi:10.1001/jamanetworkopen.2024.59945

eMethods. Inverse Probability of Treatment Weighting
eTable 1. Heterogeneity of the Treatment Effect of Methylprednisolone vs Placebo in Patients With or Without Intracranial Carotid Occlusion
eTable 2. Efficacy and Safety Outcomes of Intravenous Methylprednisolone vs Placebo Before Endovascular Thrombectomy of Patients With Intracranial Internal Carotid Artery Occlusion

This supplementary material has been provided by the authors to give readers additional information about their work.

© 2025 Zheng C et al. *JAMA Network Open.*

eMethods. Inverse Probability of Treatment Weighting

We first calculated a propensity score with treatment as the dependent variable (1 for the methylprednisolone treatment group and 0 for the control) and covariates(6 prespecified co-variates except occlusion sites (age, baseline NIHSS score, pre-stroke mRS score, baseline ASPECTS, use of intravenous thrombolysis, and time from onset to randomization) plus Stroke etiology as suggested by Reviewer listed above as independent variables through a logistic regression model in patients with intracranial carotid occlusion, and then calculate the IPTW for each subject. We calculated the treatment effect with the IPTW method in the next step. The application of these weights to the study population creates a pseudopopulation in which confounders are equally distributed across treatment and control groups.

The Standard Mean Difference prior and after IPTW.

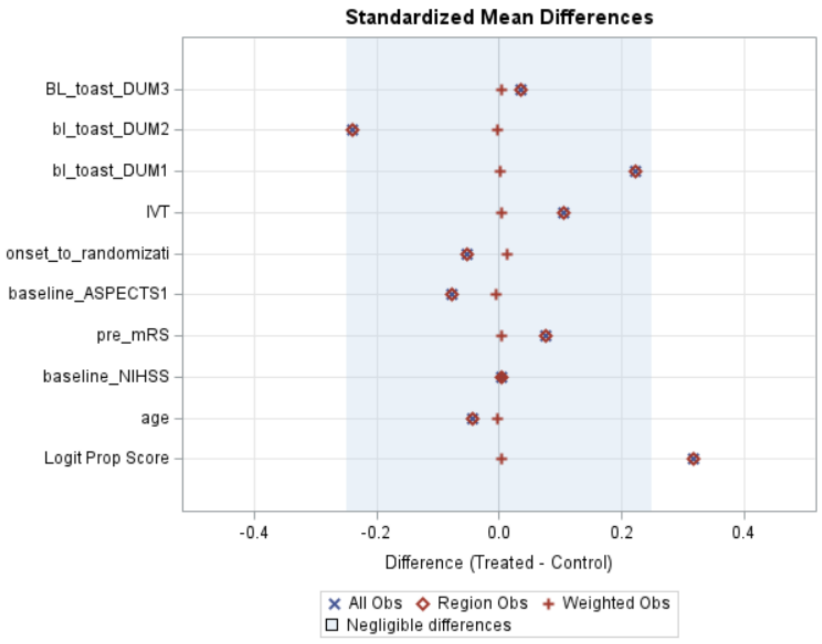

© 2025 Zheng C et al. *JAMA Network Open.*

eTable 1. Heterogeneity of the Treatment Effect of Methylprednisolone vs Placebo in Patients With or Without Intracranial Carotid Occlusion

	Unadjusted Treatment Effect	Unadjusted *P*-value for interaction[a]
Modified Rankin Scale score of 0 to 3 at 90 days		0.08
ICA	OR: 1.53 (1.10 to 2.12)	
Non-ICA	OR: 1.06 (0.83 to 1.35)	
Modified Rankin Scale score at 90 days.		0.36
ICA	cOR: 1.27 (0.95 to 1.70)	
Non-ICA	cOR: 1.09 (0.89 to 1.35)	

© 2025 Zheng C et al. *JAMA Network Open.*

eTable 2. Efficacy and Safety Outcomes of Intravenous Methylprednisolone vs Placebo Before Endovascular Thrombectomy of Patients With Intracranial Internal Carotid Artery Occlusion

**Outcome**	**Methylprednisolone (N=286)**	**Placebo (N=293)**	**Unadjusted value**	***P* value**	**IPTW model[a]**	***P* value**
**Primary outcome**						
mRS score of 0-3 at 90 d[b], no (%)	151/284 (53.2%)	125/293 (42.7%)	RR: 1.25 (1.05 to 1.48)	0.01	RR: 1.27 (1.07 to 1.52)	0.007
			OR: 1.53 (1.10 to 2.12)	0.01	OR: 1.59 (1.26 to 2.00)	0.000
			RD: 0.11 (0.02 to 0.19)	0.01	RD: 0.11 (0.06 to 0.17)	0.000
**Secondary outcomes[c]**						
mRS score at 90 d, median (IQR)	3.0 (2.0, 6.0)	4.0 (2.0, 6.0)	cOR :1.27 (0.95 to 1.70)	0.10	cOR[d]: 1.14 (0.98 to 1.32)	0.09
mRS score of 0-2 at 90 d, no (%)	107/284 (37.7%)	90 /293 (30.7%)	RR: 1.23 (0.98 to 1.54)	0.08	RR: 1.24 (0.98 to 1.56)	0.07
			OR: 1.36 (0.97 to 1.93)	0.08	OR: 1.39 (1.08 to 1.77)	0.009
			RD: 0.07 (-0.01 to 0.15)	0.08	RD: 0.07 (0.02 to 0.13)	0.009
mRS score of 0-1 at 90 d, no (%)	56/284 (19.7%)	58/293 (19.8%)	RR: 1.00 (0.72 to 1.38)	0.98	RR: 1.02 (0.73 to 1.42)	0.92
			OR: 1.00 (0.66 to 1.50)	0.98	OR: 1.02 (0.76 to 1.37)	0.88
			RD: 0 (-0.07 to 0.06)	0.98	RD: 0.00 (-0.04 to 0.05)	0.88

© 2025 Zheng C et al. *JAMA Network Open.*

Outcome	Methylprednisolone (N=286)	Placebo (N=293)	Unadjusted value	*P* value	IPTW model[a]	*P* value
NIHSS score at 5-7 d or earlier if discharged (IQR)[e]	13.0 (5.0, 32.0)	15.0 (6.0, 35.0)	Beta:-1.02 (-2.34 to 0.30)	0.13	Beta: -2.09 (-4.51 to 0.33)	0.09
EQ-5D-VAS score at 90 d(IQR)[f]	42.5 (0.0, 75.0)	30.0 (0.0, 70.0)	Beta: 3.32 (-0.22 to 6.86)	0.07	Beta: 7.18 (1.18 to 13.18)	0.02
**Safety outcomes, no (%)**						
Mortality	92/284 (32.4%)	111/293 (37.9%)	RR: 0.86 (0.68 to 1.07)	0.17	RR: 0.84 (0.67 to 1.05)	0.13
			OR: 0.79 (0.56 to 1.11)	0.17	OR: 0.77 (0.60 to 0.98)	0.03
			RD: -0.05 (-0.13 to 0.02)	0.17	RD: -0.06 (-0.12 to -0.01)	0.030
Symptomatic intracranial hemorrhage[g]	26/277 (9.4%)	45 /290(15.5%)	RR: 0.6 (0.38 to 0.95)	0.03	RR: 0.55 (0.35 to 0.87)	0.01
			OR: 0.56 (0.34 to 0.94)	0.03	OR: 0.51 (0.35 to 0.73)	0.000
			RD: -0.06 (-0.12 to -0.01)	0.03	RD: -0.07 (-0.11 to -0.03)	0.000
Any radiologic intracranialhemorrhage	113/277 (40.8%)	122 /290(42.1%)	RR: 0.97 (0.80 to 1.18)	0.76	RR: 0.94 (0.77 to 1.15)	0.55
			OR: 0.95 (0.68 to 1.33)	0.76	OR: 0.90 (0.71 to 1.14)	0.39
			RD: -0.01 (-0.09 to 0.07)	0.76	RD: -0.03 (-0.08 to 0.03)	0.39
Decompressive hemicraniectomy	16/286(5.6%)	29/293 (9.9%)	RR: 0.57 (0.31 to 1.02)	0.06	RR: 0.54 (0.3 to 0.98)	0.04
			OR: 0.54 (0.29 to 1.02)	0.06	OR: 0.52 (0.33 to 0.81)	0.004
			RD: -0.04 (-0.09 to 0.00)	0.05	RD: -0.05 (-0.08 to -0.02)	0.003
Pneumonia	139/286 (48.6%)	177/293 (60.4%)	RR: 0.8 (0.69 to 0.94)	0.005	RR: 0.80 (0.69 to 0.93)	0.004
			OR: 0.62 (0.45 to 0.86)	0.004	OR: 0.61 (0.48 to 0.77)	0.000
			RD: -0.12 (-0.20 to -0.04)	0.004	RD: -0.12 (-0.18 to -0.07)	0.000

© 2025 Zheng C et al. *JAMA Network Open.*

Outcome	Methylprednisolone (N=286)	Placebo (N=293)	Unadjusted value	*P* value	IPTW model[a]	*P* value
Gastrointestinal bleeding within 7 d after EVT	15/286 (5.2%)	20/293 (6.8%)	RR: 0.77 (0.40 to 1.47)	0.43	RR: 0.73 (0.38 to 1.42)	0.36
			OR: 0.76 (0.38 to 1.51)	0.43	OR: 0.72 (0.44 to 1.20)	0.21
			RD: -0.02 (-0.05 to 0.02)	0.42	RD: -0.02 (-0.04 to 0.01)	0.20

Abbreviations: EQ-5D-VAS, European Quality of Life 5-Dimension visual analog score; mRS, modified Rankin Scale; NIHSS, National Institutes of Health Stroke Scale; RR, risk ratio, OR: odds ratios, RD, risk difference; EVT, endovascular treatment.

[a] Adjusted values were adjusted for age, baseline NIHSS score, prestroke mRS score, baseline ASPECTS, use of intravenous thrombolysis, time from onset to randomization and stroke etiology, using the inverse probability of treatment weighting method. The RR and beta-coefficient was adjusted by the IPTW methods.

[b] The mRS of functional disability ranges from 0 (no symptoms) to 6 (death). Data were missing for 2 patients in the methylprednisolone group.

[c] The widths of the confidence intervals for the secondary outcomes were not adjusted for multiple comparisons.

[d] The cOR indicated the probability of mRS score was lower than the other group. Treatment estimation was adjusted for age, baseline NIHSS score, prestroke mRS, baseline ASPECTS, use of intravenous thrombolysis, time from onset to randomization, and stroke etiology.

[e] Scores on the NIHSS range from 0 to 42, with higher values reflecting more severe neurologic impairment.

[f] EQ-5D-VAS is a continuous scale measure of self-reported quality of life. Scores range from 0 to 100, with 0 indicating the worst possible quality of life and 100, the best possible quality of life.

[g] Symptomatic intracranial hemorrhage was defined according to the Heidelberg bleeding classification (an increase in the NIHSS score of 4 points or an increase in the score for a NIHSS subcategory of 2 points with any intracranial hemorrhage on imaging).

© 2025 Zheng C et al. *JAMA Network Open.*
