# Supplementary material for: Methylprednisolone as Adjunct to Thrombectomy for Acute Intracranial Internal Carotid Artery Occlusion Stroke: Post Hoc Secondary Analysis of the MARVEL Randomized Clinical Trial
Source: JAMA Netw Open. 2025 Feb 18;8(2):e2459945. doi: 10.1001/jamanetworkopen.2024.59945 (PMC11836765; doi:10.1001/jamanetworkopen.2024.59945)
Supplement: Supplement 3. — Data Sharing Statement [file jamanetwopen-e2459945-s003.pdf]

Data Sharing Statement

Zheng. Methylprednisolone as Adjunct to Thrombectomy for Acute Intracranial Internal Carotid Artery Occlusion Stroke. *JAMA Netw Open*. Published February 18, 2025. doi:10.1001/jamanetworkopen.2024.59945

Data
Additional Information: ChiCTR.org.cn Identifier: ChiCTR2100051729
Data available: Yes
Data types: Deidentified participant data
How to access data: The principal investigator (zhousakura@163.com) had full access to all the data in the study and takes responsibility for the integrity of the data and the accuracy of the data analysis.
When available: With publication

Supporting Documents
Document types: None

Additional Information
Who can access the data: The principal investigator (zhousakura@163.com) had full access to all the data in the study and takes responsibility for the integrity of the data and the accuracy of the data analysis.
Types of analyses: for review and meta-analysis
Mechanisms of data availability: after approval of a proposal
